# The economic burden of obesity in 4 south-eastern European countries associated with obesity-related co-morbidities

**DOI:** 10.1186/s12913-024-10840-4

**Published:** 2024-03-19

**Authors:** Kostas Athanasakis, Cornelia Bala, Alexander Kokkinos, Gabor Simonyi, Klaudia Hálová Karoliová, Amaury Basse, Miodrag Bogdanovic, Malvin Kang, Kaywei Low, Adrien Gras

**Affiliations:** 1 University of West Attica, Athens, Greece; 2 University of Medicine and Pharmacy Iuliu Hațieganu, Cluj-Napoca, Romania; 3 Medical School of the National, Kapodistrian University of Athens, Athens, Greece; 4 St. Imre University Teaching Hospital, Budapest, Hungary; 5 Department of Obesitology, Institute of Endocrinology, Prague, Czech Republic; 6 Novo Nordisk, Copenhagen, Denmark; 7 Novo Nordisk, Belgrade, Serbia; 8 Ipsos Pte Ltd, Singapore, Singapore

**Keywords:** Obesity, Financial costs, Cost analysis, Economic burden

## Abstract

**Objective:**

To provide an assessment of the cost burden of obesity across a spectrum of obesity-related comorbidities (ORCs) for four countries in South-Eastern Europe (SEE).

**Methods:**

A micro-costing analysis from the public payer perspective was conducted to estimate direct healthcare costs associated with ten obesity-related comorbidities (ORCs) in Czech Republic, Greece, Hungary, and Romania. A survey was administered to obtain healthcare resource use and unit cost data. Cost estimates were validated by local steering committees which comprised at least one public sector clinician and a panel of independent industry experts.

**Results:**

Chronic kidney disease and cardiovascular diseases were the costliest ORCs across all 4 countries, where annual cost burden per ORC exceeded 1,500 USD per patient per year. In general, costs were driven by the tertiary care resources allocated to address treatment-related adverse events, disease complications, and associated inpatient procedures.

**Conclusions:**

Our findings confirm that the high prevalence of obesity and its comorbidities result in substantial financial burden to all 4 SEE public payers. By quantifying the burden of obesity from a public healthcare perspective, our study aims to support policy efforts that promote health education and promotion in combating obesity in the region.

**Supplementary Information:**

The online version contains supplementary material available at 10.1186/s12913-024-10840-4.

## Introduction

Obesity is a chronic, relapsing and progressive disease affecting hundreds of millions globally [1]. The global obesity prevalence is expected to reach 16% and 21% in men and women respectively by 2025, affecting over 2 billion people by 2035 [1, 2]. While member states of the World Health Assembly have set voluntary targets to halt the rise of obesity, a World Obesity Federation report reveals that most countries have less than a 10% chance of meeting their targets [2]. Given that obesity is a prominent risk factor for disability and mortality associated with comorbidities, people with excess weight typically require healthcare services more often and for more complicated issues. The failure to address obesity targets thus places other key non-communicable disease (NCD) health outcomes at risk [3, 4]. 

Across Europe, overweight and obesity affect almost 60% of adults and nearly a third of children [5–7]. In Eastern Europe, about 8 in 10 of the population have overweight or obesity, and approximately one in three (32.1%) have obesity. This has translated to 25.2% of NCD-related disability-adjusted life years and 21.8% of NCD-related deaths due to high BMI (BMI > 30 kg/m^2^) [1, 8]. Type 2 diabetes (T2DM), hypertension and hyperlipidaemia are among the most critical and prevalent NCD health consequences related to obesity, among other direct effects on the cardiovascular system [9]. In Greece, a nationwide health survey observed that in 50.8% of those with hypertension and elevated cholesterol levels, obesity co-existed [10]. In Romania, the prevalence of hypertension, hyperlipidaemia and T2DM in one study were 72.8%, 64.7% and 12.6%, respectively. Further, the odds of participants with obesity to have hypertension were 2.9 times higher, and obesity increased the likelihood of developing cardiovascular disease (CVD) by 1.7 times [11]. The HAPIEE study, which investigated determinants of NCDs across 6 towns in the Czech Republic, reported hypertension, hypercholesterolemia and diabetes prevalence rates of 73%, 76.6% and 16.8%, respectively. A common risk factor for the three chronic conditions was obesity [12]. Prevalence estimates of raised blood pressure, raised cholesterol and diabetes in Hungary exceeds 30%, 50% and 7% across the general population, respectively [13]. The high prevalence of obesity-related comorbidities (ORCs) across these four health systems could lead to disastrous effects on the healthcare system if left unaddressed.

Beyond healthcare implications, obesity also presents with economic implications [14–18]. Health care cost and economic productivity estimates suggest that some countries in Eastern Europe are struggling to cope with the burden of obesity, with obesity-related disorders accounting for up to 6% of total health care expenditure [16]. In the Czech Republic, 2018 annual costs attributable to obesity accounted for 0.8% of the GDP, and direct costs totalled 600 million Euros which accounted for 3.5% of the country’s healthcare expenditure [17]. In Hungary, direct healthcare costs associated with overweight and obesity were estimated at 680 million Euros, which accounts for 11.7% of Hungarian’s healthcare budget and 0.7% of total GDP [14]. There is a pressing need for Eastern European countries to expediently address the obesity problem to avoid catastrophic economic consequences to the healthcare system, where costs are estimated to be 2.2% of GDP worldwide and even more substantial in South-Eastern Europe [19].

Across published cost burden of illness studies, no standardised methodology currently exists to guide the conduct of such studies and most of these evaluations were carried out at a macro level [14, 16, 17]. Due to these heterogeneities, transferability of cost of illness results are often limited across European countries. Further, there is limited comprehensive micro-economic evidence to guide decisions about resource allocation and appropriate interventions in the South-Eastern European (SEE) region relating to obesity and its comorbidities.

The objective of our study is thus to provide an assessment of the cost burden of obesity across a full spectrum of ORCs for four select countries in the SEE region (i.e., Czech Republic, Greece, Hungary, and Romania) with comparable GDP per capita and comparable obesity rates have surged past 20% [1, 6, 7]. In a call to action for European leaders to act urgently against obesity, our study employs a public payer perspective to contextualize our insights for European policy and decision makers.

## Methods

In this study, we sought to estimate all fixed and variable annual costs incurred by the average patient with obesity in four SEE countries (i.e., Czech Republic, Greece, Hungary, Romania) across the care continuum of 10 ORCs. The comorbidities included were aligned with those recently examined in a UK study assessing the effect of weight loss on obesity-related outcomes, and were selected for inclusion in the original study because they encompass a broad range of physiological systems, and provide reasonably comprehensive (but not exhaustive) quantification of the burden of obesity for our analysis [20]..

To overcome limitations in the availability of real-world data, a survey-based micro-costing approach was considered the most feasible and detailed method for estimating the cost of ORCs across these four countries. The approach utilizes healthcare resource use (HCRU) information provided by public sector specialists and unit cost data of every input consumed in the treatment of a particular patient to generate detailed estimates of economic costs [21]. While defined consensus standards and guidelines have yet to be developed for conducting micro-costing studies, the principles are well established in the literature.

Our study was conducted from the perspective of the public payer across the full spectrum of primary, secondary, and tertiary care settings. In the absence of a prospective data source for tracking cost and resource use across public care settings throughout the care continuum in these countries, we implemented a retrospective micro-costing analysis using a four-tiered approach [22, 23].

1. ‘Cost categories’ (i.e., HCRU and itemized resources within each HCRU category) were identified for each ORC.
2. The categories were then quantified, where a cost value was assigned to each cost item by weighting utilization against the corresponding unit prices or relevant quantities, as described by Drummond et al. [23]
3. Cost categories were subsequently aggregated to produce an overall annual cost incurred by an average patient with obesity with an ORC.
4. Aggregated costs were externally validated against published estimates in literature and by clinician academics and health economists of the respective local markets where applicable.

### Identification of cost categories

The cost categories included in our micro-costing exercise were informed by a methodical development process previously established in a study conducted in Saudi Arabia that examined the national clinical and economic burden of obesity [24]. Treatment pathways, which included screening, diagnosis, medications, and complications, were identified via a systematic literature review of clinical guidelines [24]. Keywords relating to each ORC and ‘guidelines’ or ‘systematic review’ were used to identify relevant references in PubMed and Google Scholar, which were then used to determine the types of healthcare cost parameters relevant to each ORC [24]. All cost categories was then locally validated by steering committees in the respective countries, comprising a local clinician academic and a panel of independent industry experts via focus group discussions (1 per country).

For each ORC, the following cost categories were included in the analysis: diagnostic tests per patient, scheduled outpatient visits per patient/ year, treatments received (including dose, frequency and duration), consumables and/or devices per patient/ year, monitoring tests per patient/ year, treatment-related adverse events and complications per patient/ year (which included inpatient hospitalisation, outpatient visits, intensive care unit (ICU) care, emergency room (ER) visits and inpatient procedures per patient/ year.

### Quantification of cost categories

Cost categories were consolidated in a standardized template and administered via interviewer-assisted online surveys to clinician specialists between April and July 2022 which is included in Supplementary 1. 300 clinical consultant-level specialists in the public health sector in these four countries were asked to provide HCRU estimates (Table 1). Purposive expert sampling was conducted for determining the number of survey participants, where the relevant specialists for each ORC were identified followed by the determination of sample sizes based on initial feasibility estimates for each country. As the intent of the study was to obtain costs from the public healthcare perspective, cost variations were expected not to be substantive and the sample sizes as set out in Table 1 were determined to be sufficient to result in data saturation. Survey respondents were recruited by third-party agents. All respondents were required to have been working in their specialist role for at least 3 years and be responsible for the care of at least 10 relevant patients each month. All respondents were recruited from public hospitals across a mix of geographic regions.

Unit costs for each cost item were derived from either targeted secondary research, for Czech Republic [25–27], or payer survey responses, for the other 3 SEE countries which is included in Supplementary 2. Payer respondents were either hospital administrators or hospital procurement personnel and were required to have been working in their current role for at least 3 years and be knowledgeable about hospital-related costs.

### Cost calculations

Costs were expressed in 2022 levels and foreign exchange rates described in Table 2 were used where cross-country comparisons were conducted.

The cost of each item within each cost category was calculated separately. For all items except treatments, the annual cost per patient per cost item was calculated as:

$$\begin{aligned} {\text{Annual}}\;{\text{cost}}\;{\text{per}}\;{\text{cost}}\;{\text{item}} & ={\text{Percentage (}}\% {\text{) of}}\;{\text{patients}}\\ &\quad {\text{utilizing}}\;{\text{the}}\;{\text{healthcare}}\;{\text{resource}} \\ & \times {\text{Number}}\;{\text{of}}\;{\text{healthcare}}\;{\text{resource}}\\&\quad{\text{units}}\;{\text{utilized}}\;{\text{per}}\;{\text{year}} \\ & \times {\text{Unit}}\;{\text{cost}} \\ \end{aligned} $$

The annual cost for each drug per patient was calculated as:

$$\begin{aligned} {\text{Annual}}\;{\text{cost}}\;{\text{for}}\;{\text{each}}\;{\text{drug}}\;{\text{treatment}} & ={\text{Average}}\;{\text{consumption}}\;{\text{per}}\;{\text{day}} \\ & \times 30{\text{ days}} \times 12{\text{ months}} \\ &\times {\text{Unit}}\;{\text{cost}}\;{\text{of}}\;{\text{treatment}} \\ & \times {\text{Percentage(}}\% {\text{)}}\,{\text{of}}\\&\quad{\text{patients}}\;{\text{receiving}}\;{\text{treatment}}. \\ \end{aligned} $$

Following calculation of unit costs for each ORC, the total annual cost per patient per cost categories was determined by summing all cost items. Finally, the total annual cost per patient per ORC was calculated by summing all cost categories. Cost data are presented as mean values in USD, or % of total costs.

### Data validation

Aggregated annual cost estimates were shared with respective expert steering committees in each country for final validation in focus group discussions (1 per country). In each country, a local expert steering committee was established which comprised of at least one public sector clinician academic and a panel of independent industry experts. Cost drivers and unit cost estimates were also validated by the steering committee. Where necessary, a targeted literature review was also conducted to corroborate cost data.

## Results

### Total annual costs for obesity-related comorbidities across the 4 countries

**Fig. 1:**
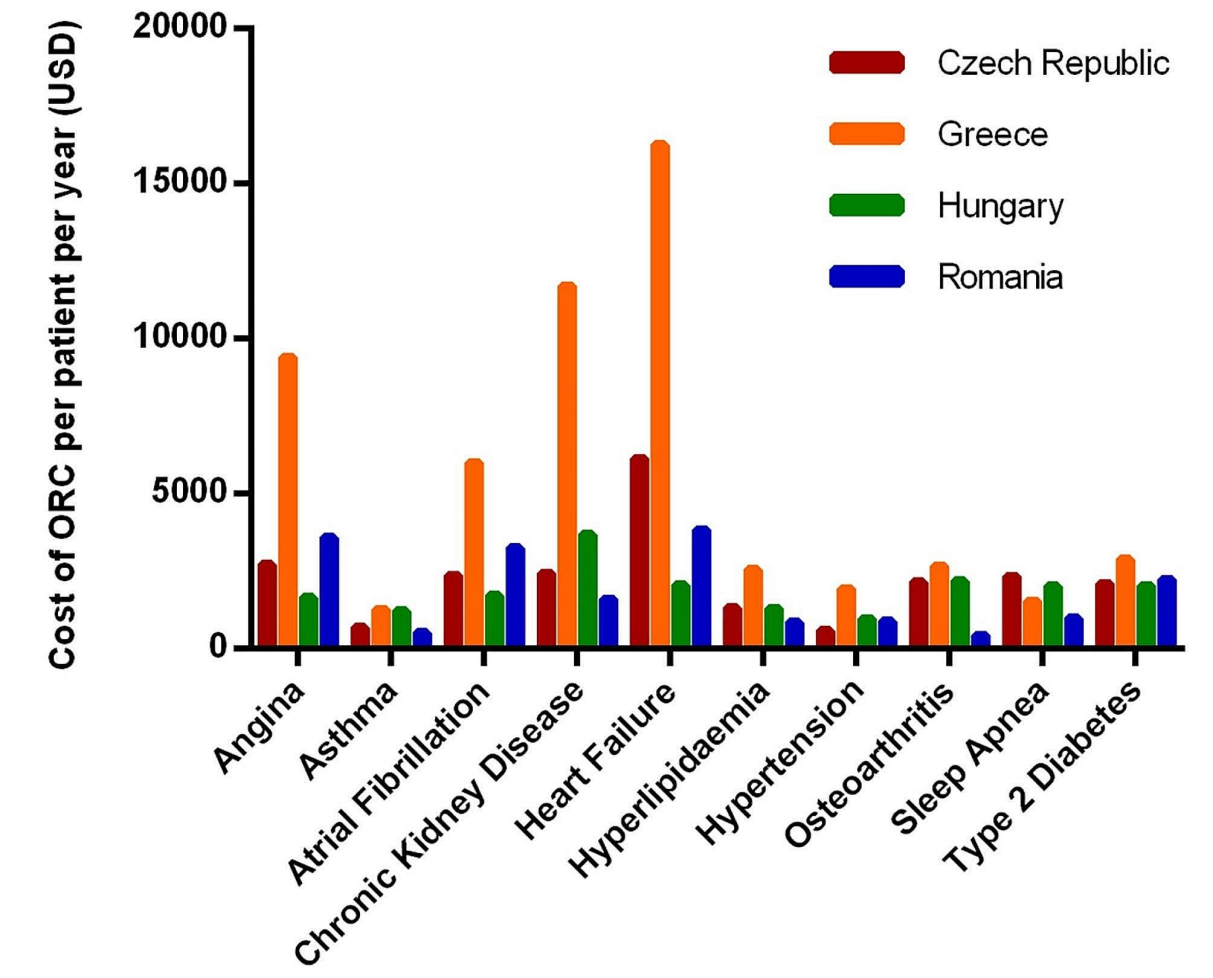
Graphical representation of estimated annual healthcare costs (USD; 2022) of ORCs in adults with obesity across the four countries

Individuals in these 4 European countries with any ORC incurred average annual healthcare costs ranging from 592 USD for hypertension in Czech Republic to 16,258 USD for heart failure in Greece (Fig. 1; Table 3). CKD and CVD comorbidities (i.e., heart failure, angina, and atrial fibrillation) were consistently the costliest comorbidities across all 4 countries, with heart failure observed to be the most expensive complication in Czech Republic, Greece, and Romania. This was driven primarily by the severity of the health states and its greater demands on relatively costly tertiary care resources in corollary. Tertiary care resources are labor- and technology-intensive which generates a greater cost burden to the public payer compared to outpatient care in the primary and community care settings.

In contrast, hypertension, asthma, and hyperlipidaemia were the least costly ORCs across all 4 countries, incurring approximately one-tenth to one-fifth of the costs of the most expensive comorbidities (Table 3). When contextualized to the disease spectrum, these ORCs are early, independent [28–31] and established risk factors of CVD and CKD. The cost impact of managing these standalone early risk factors is thus relatively cheaper in contrast to progressed disease due to simpler treatment algorithms, and fewer demands on costly tertiary care services that are inherent to disease management [32, 33]. It is worth noting that prevalence of hypertension and hyperlipidemia is high, exceeding 30% among the general population in Eastern Europe [9, 11, 12, 34, 35]. If left poorly or inadequately managed, the progression of hypertension, hyperlipidemia and asthma may lead to the development of CVD and CKD, wherein incremental costs are accrued to health system payers. Obesity prevention and comprehensive management of its complex spectrum of morbidities is thus critical to contain costs associated with its incremental and pervasive impact.

In the paragraphs that follow, we present the cost components for CKD, heart failure and T2DM, which are three key common ORCs that affect patients with obesity.

### Chronic kidney disease (CKD)

When analysing the cost of obesity-related CKD, it was observed that the cost in Greece is at least 3 times the cost estimated for Hungary, Romania and the Czech Republic. This can be attributed to variations in the overall cost of treatments, adverse events, and complications which are typically either mono- or bi-factorial in nature i.e., driven by high unit costs and/or healthcare resource utilisation (Table 4). In terms of expenditure on treatments, Greece physician respondents reported a higher percentage of CKD patients requiring haemodialysis (HD) (15%) and utilisation of erythropoietin stimulating agents (ESA) (15%) compared to the other three countries where utilisation rates were under 5%. This is corroborated by secondary literature confirming Greece has the highest incidence of HD utilization in the bundle of markets [36], suggesting a higher rate of later-stage CKD cases which also translates to higher ESA use. Unit costs for HD and ESA in Greece which contribute to a subtantive proportion of the CKD treatment costs were also substantively higher than in the other countries.

While the percentage of patients requiring tertiary healthcare services for adverse events and complications were comparable, suggesting a high degree of clinical validity across the four markets, unit costs for inpatient services were remarkably higher in Greece as compared to the other 3 countries (e.g., inpatient cost per day: Greece 481 USD vs. Czech Republic 69 USD vs. Hungary 35 USD vs. Romania 86 USD). Given the tertiary care centric nature of the healthcare system in Greece, the high utilization of tertiary care resources would translate to overall inflated ORC costs associated with incremental costs in the tertiary care sector.

### Heart failure

When analysing the cost of obesity-related heart failure, it was noted that the cost in Greece is at least 2.5 times the cost compared to the other 3 countries. Remarkable variations in costs were observed in treatments, complications and inpatient procedures which are typically either mono- or bi-factorial in nature: unit costs and/or healthcare resource utilisation (Table 5).

Greece had the highest treatment costs which was primarily due to survey-reported higher prescribing rates of guideline-directed medical therapy for heart failure compared to the other 3 countries. To illustrate, 86.3% of heart failure patients in Greece were prescribed beta blockers compared to 82.2% in Hungary, 68.8% in Czech Republic and 80% in Romania. Overall treatment costs were also driven by higher unit costs for pharmaceutical drugs in Greece, associated with the use of more expensive brand-name drugs as corroborated by experts in the steering committee. It is worth noting however, that Greece has implemented reforms to control over-prescription and a 15% threshold of total value of prescription for brand name drugs [37]. Notably, treatment costs for heart failure are also lower in the Czech Republic as sodium-glucose cotransporter-2 inhibitors (SGLT2is) were excluded from the original analysis as they were not reimbursed by the public payer at the point of survey conduct. It is noted that select SGLT2is are now a reimbursed standard of care treatment for heart failure patients in all 4 markets.

For costs of heart failure-related complications, the combination of substantially higher inpatient service unit costs in Greece (as discussed in Sect. 3.2), alongside higher resource utilization led to substantive differences. This is prominent for pulmonary oedema where over 65% of heart failure patients in Greece required inpatient stays to manage the disease complication, compared to less than 45% in the other 3 countries.

As for inpatient procedures, the higher costs observed in Greece are a consequence of a substantially higher survey-reported percentage of patients requiring inpatient procedures, such as heart valve replacement (11.5% in Greece vs. less than 6% for the other 3 countries). This observation was validated by the local steering committees. To further elaborate, the tertiary care centric nature of Greece’s healthcare system would suggest that the overall cost of care in Greece is expected to be high, in alignment with the demand for costly specialized services.

### Type 2 diabetes (T2DM)

When analysing the annual cost per patient of obesity related T2DM, it was noted that cost estimates closely align across 4 countries. Notably however, there were remarkable variations observed across 3 cost components i.e., treatments, adverse events, and inpatient procedures. These were attributed to unit cost variations in each country (Table 6). For the cost of T2DM treatments, Romania had the highest treatment costs which was primarily due to the higher unit cost of long-acting insulins and GLP-1 agonists. Greece had the highest reported adverse event cost which was contributed by a higher reported incidence of upper respiratory tract infection and higher unit costs for inpatient care. Interestingly, inpatient procedure costs in Hungary were observed to be the lowest across all 4 countries e.g., the cost of a diabetic foot amputation procedure in Hungary was USD 548 as compared to USD 1,337 in Romania, and USD 5,610 in Greece.

## Discussion

As the prevalence of obesity surges beyond 20% in Greece, Hungary, Romania and Czech Republic, there are expectations that this will place a strain on healthcare resources from both the financial and health system perspective. Our results provide an estimation on the burden of obesity on the health systems of 4 key SEE economies, highlighting areas of substantial healthcare spending across the ORC care continuum.

In general, ORCs associated with the highest annual cost per patient were found to be progressed health states such as CVD (e.g., heart failure and angina) and CKD, where the bulk of costs were contributed by pharmaceutical treatments, complications, treatment-related adverse events, and inpatient procedures, which typically require specialized tertiary care resources. Further, complications and treatment-related adverse events place substantial demand on costly hospital resources where inpatient, ICU and ER care is needed. In contrast, ORCs such as hypertension, hyperlipidemia, and asthma were associated with a lower cost per patient. It was observed that disease management required predominantly less costly outpatient care resources, with comparatively lesser specialist and tertiary care resource demands. These trends illustrate the correlation between disease severity and relative cost burden associated with the degree of tertiary care resources required. Notwithstanding, the high prevalence of hypertension and hyperlipidemia observed in all 4 markets [11, 34, 35] mean that overall costs accrued to the public health system continue to exert huge fiscal pressures.

Overall, healthcare cost estimates derived from our study vary slightly in comparison to published ORC cost estimates in other countries with similar GDP per capita and healthcare expenditure. The annual per-person healthcare cost for CKD in Czech Republic, Hungary and Romania were comparable to the costs of 2,535 USD reported in a study in Spain in 2019^38^. The Spanish study was similarly conducted from the public health perspective and representative of the average annual cost of CKD, across all-stages of severity [38]. The substantively higher CKD cost in Greece suggests that Greek patients with obesity and CKD typically have later-stage CKD which utilizes more healthcare resources especially in the tertiary care settings [36]. The cost for heart failure in the four countries were observed to be higher than the costs previously reported in Poland [39] (815 EUR in 2012; 994 EUR/ 1,035 USD in 2022) and Portugal [40] (1,159 EUR in 2014; 1,389 EUR/ 1,447 USD in 2022). Both Polish and Portuguese studies were similarly undertaken from the public health perspective and representative of chronic heart failure patients resource demands across the full spectrum of class I-IV. The higher cost of heart failure due to obesity aligns with the understanding that these patients require substantially higher healthcare resources compared to a regular heart failure patient, especially when disease complications and treatment-related adverse events occur.

While there have been studies available to quantify the burden of obesity in the European region [17, 18, 39], these studies are often limited in terms of understanding costs from a public payer perspective. Such studies are conducted on a top-down macro-level estimation basis which can include extensive cost and societal assumptions, as well as indirect costs associated with loss of workforce productivity. These studies do not accurately reflect the precise costs incurred by the public payer, which has a direct impact on healthcare spend and fiscal budgets. Our study’s bottom-up micro-costing approach from a public payer perspective provides the granularity of specific cost components that are practice and guideline-directed.

From a public health perspective, policies that target underlying risk factors such as obesity can prevent chronic diseases while potentially reducing healthcare costs in the long-term [41]. However, stretched healthcare budgets along with limited precedence in healthcare prevention hinder the ability for healthcare systems in these countries to explore health promotion or preventative care initiatives which oftentimes do not receive adequate attention. Currently, less than 3% of health spending in OECD countries is allocated to public health prevention activities which is far less than the cost burden incurred for treatment of preventable non-communicable diseases [42]. Significant policy and health system reforms to shift the concept of care from “sick” care to preventative care and health promotion will be required. As obesity is expected to be ever-increasing problem, this study would allow key stakeholders such as policymakers and healthcare professionals to understand at a micro-level on the differences in costs across the 10 ORCs and to prioritize and target resources for prevention and treatment that is commensurate with the level of cost burden. This is especially so for ORCs with higher cost burden that requires longer-term care such as chronic kidney disease where the public payer will incur more costs in the longer-term, which governments should pay greater attention and provide more resources in preventative health to reduce the incidence of ORCs of such nature. By quantifying the burden of obesity, we hope to strengthen the economic case of potential cost savings and for countries to invest in obesity-related prevention and interventions in the long-term.

### Limitations

The micro-costing approach used in this study and data sources leveraged to identify detailed healthcare costs for the different ORCs have been described as the preferred approach for settings were direct HCRU or cost data are unavailable [43, 44]. Micro-costing in the current study was based on an extensive data collection exercise, comprising nationwide surveys in each country with stringent inclusion criteria for participating healthcare providers and payers/hospital administrators. However, it should be noted that the costs derived are estimates provided by respondents instead of registry-derived costs across these countries where some specific inaccuracies are expected. In the absence of real-world data from these countries, these estimates provide a generalised understanding of the financial burden associated with obesity and its comorbidities.

Second, this study provides a comprehensive overview of the healthcare burden of obesity in these four countries but at an individual ORC level, which may lead to the assumption that a patient with obesity will only experience one ORC. A patient with obesity is likely to experience more than one ORC on average in their lifetime [45], where additional costs or care synergies may need to be considered to accurately reflect actual burden. Notwithstanding, analysing from an individual ORC angle provides a starting point to understand the variations in cost burden across the various ORCs explored in this study to further delve into potential ORC combinations when assessing burden in greater detail. This study could also form a useful resource for downstream economic evaluation exercises when assessing the value of new interventions.

Finally, it should be noted that the present study provides a conservative estimate, where it only considers the direct costs of ORCs from the public sector perspective that is reimbursed and does not include out-of-pocket costs, or indirect costs that arise from lost productivity and/or early retirement and other non-clinical components.

## Conclusion

Our results confirm that obesity and its comorbidities result in substantial financial burden to the health systems in these four countries, with potential cost savings that can be realised by preventing or delaying ORC occurrence. The study’s bottom-up micro-costing approach provides an accurate and granular perspective to the financial burden of illness from a public payer perspective due to its direct impact towards healthcare budgets.

Overall, the approach used in this study provides cost of illness estimates that could not otherwise be obtained at time of writing, demonstrating costs and HCRU across the full care continuum in a consistent and comprehensive manner. This is highly relevant for individuals, society, and policy- or decision-makers, and can be harnessed to inform translational research as well as targeted interventions against obesity and its comorbidities. Further, learnings from our study’s micro-costing approach should also be leveraged to drive efforts to optimise health information platforms that overcome current limitations around real-world patient data. A robust and reliable patient registry will facilitate data-driven decisions and enable better integrated care plans for the disease and its comorbidities. By quantifying the burden of obesity on health from a public healthcare perspective, our study aims to support policy efforts towards health education and promotion in combating obesity in the region.

Future research could consider a broader scope on assessing burden of illness and undertake a public health perspective on preventative health. Specific factors contributing to the burden of obesity and ORCs can be explored to allow policymakers and relevant stakeholders to devise targeted policies and programmes. Given the limitations of relying on survey-reported costs, the ability to access cost and HCRU data becomes crucial to better reflect the realities of cost burden from the public payer perspective. Additionally, further studies can include from various perspectives such as the patient and socioeconomic perspective to better reflect the realities of the impact of obesity has overall to a particular country and region.

**Table 1:** Specialist clinicians who contributed HCRU estimates to the micro-costing analysis

Obesity-related complication	Specialist field(s)	Czech Republic	Greece	Hungary	Romania
Type 2 Diabetes	Endocrinologists	10	10	10	10
	Internal Medicine	10	10	10	10
Asthma	Pulmonologists	10	10	10	10
Sleep apnoea	Otolaryngologists	10	10	10	10
Osteoarthritis	Orthopaedists	5	10	10	5
Chronic Kidney Disease	Nephrologists	10	10	10	10
Angina	Cardiologists	15	20	20	15
Atrial fibrillation					
Hyperlipidaemia					
Heart failure					
Hypertension					

*Abbreviations*. HCRU: healthcare resource utilisation

**Table 2:** List of currency and 2022 average foreign exchange rates

Country	Currency	Foreign Exchange Rate (1 USD)
Czech Republic	Czech Koruna (CZK)	0.043
Greece	Euro (EUR)	1.04
Hungary	Hungarian Forint (HUF)	0.0027
Romania	Romanian Leu (RON)	0.21

**Table 3:** Estimated annual healthcare costs (USD; 2022) of ORCs in adults with obesity across the four countries

ORCs	Cost of ORC per patient per year (USD)			
	Czech Republic	Greece	Hungary	Romania
Angina	2,717	9,410	1,666	3,597
Asthma	691	1,272	1,219	527
Atrial Fibrillation	2,370	6,004	1,723	3,258
Chronic Kidney Disease	2,438	11,708	3,684	1,616
Heart Failure	6,144	16,258	2,051	3,848
Hyperlipidaemia	1,318	2,558	1,311	859
Hypertension	592	1,946	979	897
Osteoarthritis	2,155	2,681	2,188	449
Sleep Apnea	2,342	1,540	2,032	987
Type 2 Diabetes	2,105	2,888	2,042	2,248

*Abbreviations*. ORC: Obesity-related comorbidity

**Table 4:** Cost breakdown by components for chronic kidney disease (CKD) across the 4 countries

	Czech Republic	Greece	Hungary	Romania
Diagnostics	45	265	140	59
Outpatient visits	65	119	401	101
Treatments	787	4,601	1,415	280
Consumables/ Devices	0	53	11	0
Monitoring tests	87	329	214	81
Adverse events	506	1,973	562	131
Complications	502	4,187	760	285
Inpatient procedures	448	385	181	678
**Total (USD)**	**2,438**	**11,708**	**3,684**	**1,616**

**Table 5:** Cost breakdown by components for heart failure across the 4 countries

	Czech Republic	Greece	Hungary	Romania
Diagnostics	68	321	251	116
Outpatient visits	76	75	246	122
Treatments	60	517	106	310
Monitoring tests	65	518	258	126
Adverse events	304	665	178	371
Complications	850	2,337	191	701
Inpatient procedures	4,722	11,825	821	2,102
**Total (USD)**	**6,144**	**16,258**	**2,051**	**3,848**

**Table 6:** Cost breakdown by components for T2DM across the 4 countries

	Czech Republic	Greece	Hungary	Romania
Diagnostics	11	47	40	21
Outpatient visits	61	88	312	109
Treatments	124	358	568	589
Monitoring tests	23	253	0	48
Adverse events	195	547	60	125
Complications	308	598	337	379
Inpatient procedures	1,234	793	128	911
**Total (USD)**	**2,105**	**2,888**	**2,042**	**2,248**

### Electronic supplementary material

Below is the link to the electronic supplementary material.

Supplementary Material 1

Supplementary Material 2

## Data Availability

The datasets used and analysed during the current study are available from the corresponding author on reasonable request.
