# Supplementary material for: The economic burden of obesity in 4 south-eastern European countries associated with obesity-related co-morbidities
Source: BMC Health Serv Res. 2024 Mar 19;24:354. doi: 10.1186/s12913-024-10840-4 (PMC10953276; doi:10.1186/s12913-024-10840-4)
Supplement: Supplementary file 1 — Supplementary Material 1 [file 12913_2024_10840_MOESM1_ESM.docx]

**Additional File 1 – HCP Questionnaire**

| **A: SCREENER** |
| --- |

| S0 | **ASK ALL, SINGLE CHOICE (S/C)**  Please indicate the country that you practice in.  **Please select one only**   \| Hungary (HUN) \| **1** \| **CONTINUE** \| \| --- \| --- \| --- \| \| Romania (ROM) \| **2** \| \| Czech Republic (CZ) \| **3** \| \| Greece (GR) \| **4** \| \| Others \| **5** \| **TERMINATE** \| |
| --- | --- | --- | --- | --- | --- | --- | --- | --- | --- | --- | --- | --- | --- |
| S1 | **ASK ALL, S/C**  Please indicate your current primary specialty.  **Please select one only**   \| Diabetologist **Internist [FOR GR only]** \| **1** \| **CONTINUE** \| \| --- \| --- \| --- \| \| Internal Medicine specialist **[FOR CZ only]** \| **2** \| \| Endocrinologist **[N/A for CZ]** \| **3** \| \| Pulmonologist \| **4** \| \| Otolaryngologist **[N/A for GR]** \| **5** \| \| Orthopedist/Orthopedic \| **6** \| \| Cardiologist \| **7** \| \| Nephrologist \| **8** \| \| Primary care physician (PCP) / General Practitioner (GPS) **[CONTINUE FOR CZ and GR only]** \| **9** \| **TERMINATE** \| \| Other, please specify \| **99** \| |
| S2 | **ASK ALL, RANGE 0-99**  How many years have you been qualified in your current specialty?  **Please enter a number**   \| __________ Years \| **1** \| **TERMINATE IF <3, OR >30 [N/A FOR HUN]**  **TERMINATE IF <3, OR >40 [FOR HUN ONLY]** \| \| --- \| --- \| --- \| |
| S3 | **ASK ALL, RANGE 1-9999**  How many patients in total (all indications) do you have under your care in a typical month?  **Please enter a number**   \| __________ Patients \| **1** \| **CONTINUE** \| \| --- \| --- \| --- \| |
| S4 | **ASK ALL, RANGE 0-9999**  **TOTAL SUM SHOULD BE = OR < S3 [N/A FOR RESPONDENTS WHO ARE ANSWERING MULTIPLE COMORBIDITIES]**  Of all the patients you have under your care in a typical month, how many have been diagnosed with each of the following conditions?  **Please enter a number for each**   \| Type 2 diabetes **[SHOW FOR DIABETOLOGISTS S1 CODE 1, INTERNAL MEDICINE SPECIALISTS S1 CODE 2, ENDOCRINOLOGISTS S1 CODE 3 AND PCP/GPS S1 CODE 9 ONLY]** \| ______ patients \| **A** \| **TERMINATE IF DIABETOLOGIST OR INTERNAL MEDICINE SPECIALIST OR ENDOCRINOLOGIST OR PCP/GPS AND <10** \| \| --- \| --- \| --- \| --- \| \| Asthma **[SHOW FOR PULMONOLOGISTS S1 CODE 4 ONLY]** \| ______ patients \| **B** \| **TERMINATE IF PULMONOLOGIST AND <10** \| \| Sleep apnea **[SHOW FOR PULMONOLOGISTS S1 CODE 4 [FOR GR ONLY], OTOLARYNGOGISTS S1 CODE 5 ONLY]** \| ______ patients \| **C** \| **TERMINATE IF PULMONOLOGIST/ Otolaryngologist AND <10** \| \| Osteoarthritis **[SHOW FOR ORTHOPEDISTS S1 CODE 6 ONLY]** \| ______ patients \| **D** \| **TERMINATE IF ORTHOPEDIST AND <10** \| \| Heart failure **[SHOW FOR CARDIOLOGISTS S1 CODE 7 ONLY]** \| ______ patients \| **E** \| **TERMINATE IF CARDIOLOGISTS AND <10** \| \| Hypertension **[SHOW FOR INTERNAL MEDICINE SPECIALISTS S1 CODE 2 (IN CZ ONLY), CARDIOLOGISTS S1 CODE 7 AND PCP/GPS S1 CODE 9 ONLY]** \| ______ patients \| **F** \| **TERMINATE IF CARDIOLOGISTS OR PCP/GPS AND <10** \| \| Dyslipidemia **[SHOW FOR INTERNAL MEDICINE SPECIALISTS S1 CODE 2 (IN CZ ONLY), CARDIOLOGISTS S1 CODE 7 AND PCP/GPS S1 CODE 9 ONLY]** \| ______ patients \| **G** \| **TERMINATE IF CARDIOLOGISTS OR PCP/GPS AND <10** \| \| Atrial fibrillation **[SHOW FOR CARDIOLOGISTS S1 CODE 7 ONLY]** \| ______ patients \| **H** \| **TERMINATE IF CARDIOLOGISTS AND <10** \| \| Angina **[SHOW FOR CARDIOLOGISTS S1 CODE 7 ONLY]** \| ______ patients \| **I** \| **TERMINATE IF CARDIOLOGISTS AND <10** \| \| Chronic kidney disease **[SHOW FOR NEPHROLOGISTS S1 CODE 8 ONLY]** \| ______ patients \| **J** \| **TERMINATE IF NEPHROLOGIST AND <10** \| |
| S5 | In which of the following care settings do you spend most of your professional time managing and treating patients?  **Please select one only**  **ASK FOR HUNGARY, SC**   \| Clinical centres \| **A** \| **TERMINATE IF SELECT CODE H or I** \| \| --- \| --- \| --- \| \| Municipal (city) hospital \| **B** \| \| County hospital \| **C** \| \| National specialist institute \| **D** \| \| Specialist hospital/clinics \| **E** \| \| University/teaching hospital \| **F** \| \| Military/homeland defence hospital \| **G** \| \| Private hospital/clinic \| **H** \| \| Other, please specify \| **I** \|   **ASK FOR CZECH REPUBLIC, SC**   \| Public general hospital \| **A** \| **TERMINATE IF SELECT CODE G or CODE H**  **CODE G_1 and G_2 APPLICABLE FOR GPs/PCP only** \| \| --- \| --- \| --- \| \| Public general faculty hospital/Public university hospital \| **B** \| \| Public regional hospital \| **C** \| \| Public university hospital \| **D** \| \| Public specialty hospital \| **E** \| \| Public military hospital \| **F** \| \| Private hospital \| **G** \| \| Regional public clinics **[FOR GPs/PCP only]** \| **G_1** \| \| Private clinic/Private practice **[FOR GPs/PCP only]** \| **G_2** \| \| Other, please specify \| **H** \|   **ASK FOR ROMANIA, SC**   \| Public Municipal Hospital \| **A** \| **TERMINATE IF SELECT CODE D or E** \| \| --- \| --- \| --- \| \| Public County Emergency Hospital \| **B** \| \| Public Town Hospital \| **C** \| \| Private hospital \| **D** \| \| Other, please specify \| **E** \|   **ASK FOR GREECE, SC**   \| Hospital setting \| **A** \| **FOR ALL SPECIALTIES OTHER THAN GPS: 50% OF SAMPLE FROM HOSPITAL SETTING AND 50% FROM PRIVATE PRACTICE SETTING**  **For GPs: 100% IN PRIVATE PRACTICE SETTING AND <10% OF SAMPLE FROM ATHENS**  **TERMINATE IF SELECT CODE C** \| \| --- \| --- \| --- \| \| Private practice HCPs \| **B** \| \| Other, please specify \| **C** \| |

| **B: OUT-PATIENT HCRU** |
| --- |

This survey is focused on [insert comorbidity shown at S4] in the main care setting in which you work [insert code selected at S6]**.** Please only consider these patients when answering the survey questions.

| Q1 | **PIPE IN SELECTION FROM S1**  **SHOW GRID TABLE WITH PRE-FILLED RANGES, ALLOW RESPONDENTS TO OVERWRITE ON CELLS**  **SHOW PROMPT IF INPUT IS OUT OF RANGE SPECIFIED**  **DO NOT ALLOW PART B TO BE ENTERED IF PART A WAS CODED 0 for “% patients”**  **TOTAL SUM FOR Q1a GREATER THAN OR = 100**   1. Which of these tests do you use for diagnosing your patients with [insert comorbidity shown at S4 [Note: This is solely for initial diagnosis to confirm a medical condition and not for ongoing monitoring, which will be covered in a later section]?   **Please enter the percentage of patients who undergo each test within the stated range.**   1. On average, how many times will each test be undertaken to confirm an initial diagnosis of [insert comorbidity]? [Note: This is solely for initial diagnosis to confirm a medical condition and not for ongoing monitoring, which will be covered in a later section]   **Please enter a number for each within the stated range**   \|  \| **Initial diagnostic tests** \| \| --- \| --- \| \|  \|  \| **% Patients**  **(range in brackets)** \| **Number of tests per patient (range in brackets)** \| \|  \| **T2D** \|  \|  \| \| **1** \| Fasting Plasma Glucose \| (Range: 0 – 100) \| (Range: 1 – 10) \| \| **2** \| Random Plasma Glucose \| (Range: 0 – 100) \| (Range: 1 – 10) \| \| **3** \| Two-hour Oral Glucose Tolerance Test \| (Range: 0 – 100) \| (Range: 1 – 10) \| \| **4** \| Glycated hemoglobin (HbA1C) \| (Range: 0 – 100) \| (Range: 1 – 10) \| \| **5** \| Renal function test **[FOR CZ ONLY]** \| (Range: 0 – 100) \| (Range: 1 – 10) \| \| **6** \| Urinalysis **[FOR CZ ONLY]** \| (Range: 0 – 100) \| (Range: 1 – 10) \| \|  \| **Asthma** \|  \| (Range: 1 – 10) \| \| **7** \| Spirometry \| (Range: 0 – 100) \| (Range: 1 – 10) \| \| **8** \| Chest X-ray \| (Range: 0 – 100) \| (Range: 1 – 10) \| \| **9** \| Peripheral eosinophilia and elevated IgE level \| (Range: 0 – 100) \| (Range: 1 – 10) \| \| **10** \| Exhaled nitric oxide **[N/A FOR HUN]** \| (Range: 0 – 100) \| (Range: 1 – 10) \| \| **11** \| Skin prick and radioallergosorbent test (RAST) \| (Range: 0 – 100) \| (Range: 1 – 10) \| \|  \| **Sleep apnea** \|  \|  \| \| **12** \| Attended, in-laboratory polysomnography \| (Range: 0 – 100) \| (Range: 1 – 10) \| \| **13** \| Portable monitoring (1 day) \| (Range: 0 – 100) \| (Range: 1 – 10) \| \| **14** \| Overnight pulse oximetry (1 night) \| (Range: 0 – 100) \| (Range: 1 – 10) \| \| **15** \| Portable monitoring of cardiopulmonary channels (1 day) \| (Range: 0 – 100) \| (Range: 1 – 10) \| \|  \| **Osteoarthritis** \|  \|  \| \| **16** \| X-ray \| (Range: 0 – 100) \| (Range: 1 – 10) \| \| **17** \| MRI / Magnetic resonance imaging \| (Range: 0 – 100) \| (Range: 1 – 10) \| \| **18** \| Complete blood count \| (Range: 0 – 100) \| (Range: 1 – 10) \| \| **19** \| Erythrocyte sedimentation rate \| (Range: 0 – 100) \| (Range: 1 – 10) \| \| **20** \| Rheumatoid factor \| (Range: 0 – 100) \| (Range: 1 – 10) \| \| **21** \| ANA/ Antinuclear antibodies \| (Range: 0 – 100) \| (Range: 1 – 10) \| \| **22** \| Synovial fluid analysis \| (Range: 0 – 100) \| (Range: 1 – 10) \| \|  \| **Heart failure** \|  \|  \| \| **23** \| ECG / Electrocardiogram \| (Range: 0 – 100) \| (Range: 1 – 10) \| \| **24** \| Bedside echocardiogram \| (Range: 0 – 100) \| (Range: 1 – 10) \| \| **25** \| Chest X-ray \| (Range: 0 – 100) \| (Range: 1 – 10) \| \| **26** \| Complete blood count \| (Range: 0 – 100) \| (Range: 1 – 10) \| \| **27** \| Serum electrolytes \| (Range: 0 – 100) \| (Range: 1 – 10) \| \| **28** \| Renal function test \| (Range: 0 – 100) \| (Range: 1 – 10) \| \| **29** \| Liver function test \| (Range: 0 – 100) \| (Range: 1 – 10) \| \| **30** \| Urinalysis **[N/A FOR CZ]** \| (Range: 0 – 100) \| (Range: 1 – 10) \| \| **31** \| Fasting plasma glucose test **[N/A FOR CZ]** \| (Range: 0 – 100) \| (Range: 1 – 10) \| \| **32** \| Oral glucose tolerance test **[N/A FOR CZ]** \| (Range: 0 – 100) \| (Range: 1 – 10) \| \| **33** \| Lipid panel \| (Range: 0 – 100) \| (Range: 1 – 10) \| \| **34** \| Thyroid hormone determination \| (Range: 0 – 100) \| (Range: 1 – 10) \| \| **35** \| B-type natriuretic peptide (BNP)/ N-terminal pro B-type natriuretic peptide (NT-proBNP) \| (Range: 0 – 100) \| (Range: 1 – 10) \| \| **36** \| HbA1c **[FOR ROM only]** \| (Range: 0 – 100) \| (Range: 1 – 10) \| \|  \| **Hypertension** \|  \|  \| \| **37** \| Fasting plasma glucose **[N/A FOR CZ]** \| (Range: 0 – 100) \| (Range: 1 – 10) \| \| **38** \| Serum total cholesterol, low-density lipoprotein cholesterol, high-density lipoprotein cholesterol \| (Range: 0 – 100) \| (Range: 1 – 10) \| \| **39** \| Fasting serum triglycerides \| (Range: 0 – 100) \| (Range: 1 – 10) \| \| **40** \| Serum potassium and sodium \| (Range: 0 – 100) \| (Range: 1 – 10) \| \| **41** \| Serum uric acid \| (Range: 0 – 100) \| (Range: 1 – 10) \| \| **42** \| Serum creatinine \| (Range: 0 – 100) \| (Range: 1 – 10) \| \| **43** \| Estimated glomerular filtration rate (eGFR) \| (Range: 0 – 100) \| (Range: 1 – 10) \| \| **44** \| Urine analysis including a test for microalbuminuria \| (Range: 0 – 100) \| (Range: 1 – 10) \| \| **45** \| 12-lead ECG \| (Range: 0 – 100) \| (Range: 1 – 10) \| \| **45.1** \| UACR test **[FOR GR ONLY]** \| (Range: 0 – 100) \| (Range: 1 – 10) \| \|  \| **Dyslipidemia** \|  \|  \| \| **46** \| Lipid panel \| (Range: 0 – 100) \| (Range: 1 – 10) \| \| **47** \| Fasting plasma glucose \| (Range: 0 – 100) \| (Range: 1 – 10) \| \| **48** \| HbA1C \| (Range: 0 – 100) \| (Range: 1 – 10) \| \| **49** \| Liver enzymes \| (Range: 0 – 100) \| (Range: 1 – 10) \| \| **50** \| BUN, Creatinine, GFR \| (Range: 0 – 100) \| (Range: 1 – 10) \| \| **51** \| Creatine phosphokinase \| (Range: 0 – 100) \| (Range: 1 – 10) \| \| **52** \| ECG / Electrocardiogram \| (Range: 0 – 100) \| (Range: 1 – 10) \| \|  \| **Atrial Fibrillation** \|  \|  \| \| **53** \| 12-L ECG \| (Range: 0 – 100) \| (Range: 1 – 10) \| \| **54** \| Complete blood count \| (Range: 0 – 100) \| (Range: 1 – 10) \| \| **55** \| Metabolic panel \| (Range: 0 – 100) \| (Range: 1 – 10) \| \| **56** \| Thyroid function test \| (Range: 0 – 100) \| (Range: 1 – 10) \| \| **57** \| Chest X-ray \| (Range: 0 – 100) \| (Range: 1 – 10) \| \| **58** \| Spiral CT scan **[N/A FOR HUN & ROM]** \| (Range: 0 – 100) \| (Range: 1 – 10) \| \| **59** \| Transesophageal echocardiogram \| (Range: 0 – 100) \| (Range: 1 – 10) \| \| **60** \| Transthoracic echocardiogram \| (Range: 0 – 100) \| (Range: 1 – 10) \| \| **61** \| Cardiac catheterization **[N/A FOR HUN]** \| (Range: 0 – 100) \| (Range: 1 – 10) \| \|  \| **Angina** \|  \|  \| \| **62** \| Complete blood count \| (Range: 0 – 100) \| (Range: 1 – 10) \| \| **63** \| Metabolic panel \| (Range: 0 – 100) \| (Range: 1 – 10) \| \| **64** \| Urinalysis \| (Range: 0 – 100) \| (Range: 1 – 10) \| \| **65** \| Chest X-ray \| (Range: 0 – 100) \| (Range: 1 – 10) \| \| **66** \| 12-L ECG \| (Range: 0 – 100) \| (Range: 1 – 10) \| \| **67** \| Cardiac biomarker test \| (Range: 0 – 100) \| (Range: 1 – 10) \| \| **68** \| Coronary angiography \| (Range: 0 – 100) \| (Range: 1 – 10) \| \| **68.1** \| HbA1c **[FOR ROM only]** \| (Range: 0 – 100) \| (Range: 1 – 10) \| \|  \| **Chronic Kidney Disease** \|  \|  \| \| **69** \| Complete blood count \| (Range: 0 – 100) \| (Range: 1 – 10) \| \| **70** \| Metabolic panel \| (Range: 0 – 100) \| (Range: 1 – 10) \| \| **71** \| Lipid panel \| (Range: 0 – 100) \| (Range: 1 – 10) \| \| **72** \| Urinalysis \| (Range: 0 – 100) \| (Range: 1 – 10) \| \| **73** \| Kidney biopsy \| (Range: 0 – 100) \| (Range: 1 – 10) \| \| **74** \| Renal ultrasound \| (Range: 0 – 100) \| (Range: 1 – 10) \| \| **75** \| Serum calcium \| (Range: 0 – 100) \| (Range: 1 – 10) \| \| **76** \| UACR test **[FOR GR ONLY]** \| (Range: 0 – 100) \| (Range: 1 – 10) \| |
| --- | --- | --- | --- | --- | --- | --- | --- | --- | --- | --- | --- | --- | --- | --- | --- | --- | --- | --- | --- | --- | --- | --- | --- | --- | --- | --- | --- | --- | --- | --- | --- | --- | --- | --- | --- | --- | --- | --- | --- | --- | --- | --- | --- | --- | --- | --- | --- | --- | --- | --- | --- | --- | --- | --- | --- | --- | --- | --- | --- | --- | --- | --- | --- | --- | --- | --- | --- | --- | --- | --- | --- | --- | --- | --- | --- | --- | --- | --- | --- | --- | --- | --- | --- | --- | --- | --- | --- | --- | --- | --- | --- | --- | --- | --- | --- | --- | --- | --- | --- | --- | --- | --- | --- | --- | --- | --- | --- | --- | --- | --- | --- | --- | --- | --- | --- | --- | --- | --- | --- | --- | --- | --- | --- | --- | --- | --- | --- | --- | --- | --- | --- | --- | --- | --- | --- | --- | --- | --- | --- | --- | --- | --- | --- | --- | --- | --- | --- | --- | --- | --- | --- | --- | --- | --- | --- | --- | --- | --- | --- | --- | --- | --- | --- | --- | --- | --- | --- | --- | --- | --- | --- | --- | --- | --- | --- | --- | --- | --- | --- | --- | --- | --- | --- | --- | --- | --- | --- | --- | --- | --- | --- | --- | --- | --- | --- | --- | --- | --- | --- | --- | --- | --- | --- | --- | --- | --- | --- | --- | --- | --- | --- | --- | --- | --- | --- | --- | --- | --- | --- | --- | --- | --- | --- | --- | --- | --- | --- | --- | --- | --- | --- | --- | --- | --- | --- | --- | --- | --- | --- | --- | --- | --- | --- | --- | --- | --- | --- | --- | --- | --- | --- | --- | --- | --- | --- | --- | --- | --- | --- | --- | --- | --- | --- | --- | --- | --- | --- | --- | --- | --- | --- | --- | --- | --- | --- | --- | --- | --- | --- | --- | --- | --- | --- | --- | --- | --- | --- | --- | --- | --- | --- | --- | --- | --- | --- | --- | --- | --- | --- | --- | --- | --- | --- | --- | --- | --- | --- | --- | --- | --- | --- | --- | --- | --- | --- | --- | --- | --- | --- | --- | --- | --- | --- | --- | --- | --- | --- | --- | --- | --- | --- | --- | --- | --- | --- | --- | --- | --- | --- | --- | --- | --- | --- | --- | --- | --- | --- | --- | --- | --- | --- | --- | --- | --- | --- | --- | --- | --- | --- |
| Q2 | **ASK ALL**  **SHOW GRID TABLE WITH PRE-FILLED RANGES, ALLOW RESPONDENTS TO OVERWRITE ON CELLS**  **SHOW PROMPT IF INPUT IS OUT OF RANGE SPECIFIED**  **MINIMUM SUM OF TOTAL COLUMN = 1**  **PIPE IN SELECTION FROM S1**  In an average year, how many routine/scheduled out-patient visits (excluding off-schedule/emergency visits) does a patient have in relation to their [insert comorbidity from S4] with each of the following Specialties?  **Please enter a number for each setting within the stated range**   \|  \| **Out-patient visits** \| **Number of outpatient visits per year**  **(range in brackets)** \| \| --- \| --- \| --- \| \|  \| **T2D** \|  \| \| **1** \| Diabetologist visits \| (Range: 0 - 52) \| \| **2** \| Endocrinologist visits \| (Range: 0 - 52) \| \| **3** \| GP visits \| (Range: 0 - 52) \| \| **4** \| Community nurse visits **[N/A FOR GR & CZ]** \| (Range: 0 - 52) \| \|  \| **Asthma** \|  \| \| **5** \| Internist visits \| (Range: 0 - 52) \| \| **6** \| Pulmonologist visits \| (Range: 0 - 52) \| \| **7** \| GP visits \| (Range: 0 - 52) \| \| **8** \| Community nurse visits **[N/A FOR GR & CZ]** \| (Range: 0 - 52) \| \|  \| **Sleep apnea** \|  \| \| **9** \| ENT/Otolaryngologist visit \| (Range: 0 - 52) \| \| **10** \| GP visits \| (Range: 0 - 52) \| \| **10.1** \| Pulmonologist visits **[FOR GR ONLY]** \| (Range: 0 - 52) \| \| **11** \| Community nurse visits **[N/A FOR GR & CZ]** \| (Range: 0 - 52) \| \|  \| **Osteoarthritis** \|  \| \| **12** \| Physiotherapist visits \| (Range: 0 - 52) \| \| **13** \| Chiropractor visits **[N/A FOR GR]** \| (Range: 0 - 52) \| \| **14** \| Osteopath visits **[N/A FOR GR]** \| (Range: 0 - 52) \| \| **15** \| GP visits \| (Range: 0 - 52) \| \| **15.1** \| Orthopedic specialist visits **[FOR GR ONLY]** \| (Range: 0 - 52) \| \| **15.2** \| Rheumatologists visits **[FOR GR ONLY]** \| (Range: 0 - 52) \| \| **16** \| Community nurse visits **[N/A FOR GR & CZ]** \| (Range: 0 - 52) \| \|  \| **Heart failure** \|  \| \| **17** \| Cardiologist/internist visits **[ADD INTERNIST FOR GR ONLY]** \| (Range: 0 - 52) \| \| **18** \| GP visits \| (Range: 0 - 52) \| \| **19** \| Community nurse visits **[N/A FOR GR & CZ]** \| (Range: 0 - 52) \| \|  \| **Hypertension** \|  \| \| **20** \| Cardiologist/internist visits **[ADD INTERNIST FOR GR ONLY]** \| (Range: 0 - 52) \| \| **21** \| GP visits \| (Range: 0 - 52) \| \| **22** \| Community nurse visits **[N/A FOR GR & CZ]** \| (Range: 0 - 52) \| \|  \| **Atrial fibrillation** \|  \| \| **23** \| Cardiologist/internist visits **[ADD INTERNIST FOR GR ONLY]** \| (Range: 0 - 52) \| \| **24** \| GP visits \| (Range: 0 - 52) \| \| **25** \| Community nurse visits **[N/A FOR GR & CZ]** \| (Range: 0 - 52) \| \|  \| **Angina** \|  \| \| **26** \| Cardiologist/internist visits **[ADD INTERNIST FOR GR ONLY]** \| (Range: 0 - 52) \| \| **27** \| GP visits \| (Range: 0 - 52) \| \| **28** \| Community nurse visits **[N/A FOR GR & CZ]** \| (Range: 0 - 52) \| \|  \| **Dyslipidemia** \|  \| \| **29** \| Cardiologist/internist visits **[ADD INTERNIST FOR GR ONLY]** \| (Range: 0 - 52) \| \| **30** \| Endocrinologist visits \| (Range: 0 - 52) \| \| **31** \| GP visits \| (Range: 0 - 52) \| \| **32** \| Community nurse visits **[N/A FOR GR & CZ]** \| (Range: 0 - 52) \| \|  \| **Chronic Kidney Disease** \|  \| \| **33** \| Nephrologist visits \| (Range: 0 - 52) \| \| **34** \| GP visits \| (Range: 0 - 52) \| \| **34.1** \| Internist visits **[FOR GR ONLY]** \| (Range: 0 - 52) \| \| **34.2** \| Diabetologist visits **[FOR GR ONLY]** \| (Range: 0 - 52) \| \| **35** \| Community nurse visits **[N/A FOR GR & CZ]** \| (Range: 0 - 52) \| |
| **Q2.1** | **ASK ALL, SC**  **PIPE IN SELECTION FROM S1**  **SHOW GRID TABLE**  **NOTE TO SCRIPTING TEAM: NO RESTRICTION ON SUM IN COLUMN A**   1. What proportion of your patients diagnosed with [insert comorbidity from S4], receive each of the following treatments for their condition in an average year?   **Please enter a percentage for each drug class**   \|  \| **Treatments** \| **A**  **% patients receiving drug in a year**  **(range in brackets)** \| \| --- \| --- \| --- \| \| **T2D** \| \| \| \| **1** \| Biguanides (e.g. Metformin) \| (Range: 0 - 100) \| \| **2** \| Dipeptidyl peptidase 4 (DPPIV Inhibitors) \| (Range: 0 - 100) \| \| **3** \| Sodium-glucose cotransporter inhibitors \| (Range: 0 - 100) \| \| **4** \| Neutral Protamine Hagedorn insulins \| (Range: 0 - 100) \| \| **5** \| Short-acting insulins \| (Range: 0 - 100) \| \| **6** \| Long-acting insulins \| (Range: 0 - 100) \| \| **7** \| Pre-Mixed Insulin \| (Range: 0 - 100) \| \| **8** \| GLP-1 agonists \| (Range: 0 - 100) \| \| **9** \| Basal and GLP-1 fixed ratio combinations \| (Range: 0 - 100) \| \| **10** \| Sulfonylureas \| (Range: 0 - 100) \| \| **11** \| Thiazolidinedione \| (Range: 0 - 100) \| \| **12** \| Alpha-glucosidase enzyme inhibitors **[FOR CZ ONLY]** \| (Range: 0 - 100) \| \| **12.1** \| Meglitinides **[FOR GR ONLY]** \| (Range: 0 - 100) \| \| **Asthma** \| \| \| \| **13** \| B-selective agonist (short-acting) \| (Range: 0 - 100) \| \| **14** \| B-selective agonist (long-acting) \| (Range: 0 - 100) \| \| **15** \| Muscarinic receptor agonist \| (Range: 0 - 100) \| \| **16** \| Methylxanthine \| (Range: 0 - 100) \| \| **17** \| Mast cell stabilizers \| (Range: 0 - 100) \| \| **18** \| Corticosteroids \| (Range: 0 - 100) \| \| **19** \| Leukotriene synthesis inhibitors \| (Range: 0 - 100) \| \| **20** \| Leukotriene receptor antagonist \| (Range: 0 - 100) \| \| **21** \| Biologic agents \| (Range: 0 - 100) \| \| **Osteoarthritis** \| \| \| \| **25** \| Acetaminophen/Paracetamol \| (Range: 0 - 100) \| \| **26** \| Nonsteroidal Anti-inflammatory Drugs (NSAIDS) \| (Range: 0 - 100) \| \| **27** \| Glucocorticoids \| (Range: 0 - 100) \| \| **28** \| Hyaluronic acid \| (Range: 0 - 100) \| \| **29** \| Opioids \| (Range: 0 - 100) \| \| **30** \| Duloxetine \| (Range: 0 - 100) \| \| **Heart failure** \| \| \| \| **31** \| Diuretics \| (Range: 0 - 100) \| \| **32** \| Angiotensin converting enzyme inhibitors \| (Range: 0 - 100) \| \| **33** \| Beta blockers \| (Range: 0 - 100) \| \| **34** \| Aldosterone antagonist \| (Range: 0 - 100) \| \| **35** \| Angiotensin receptor blockers \| (Range: 0 - 100) \| \| **35.1** \| Sodium-glucose cotransporter inhibitors **[FOR GR ONLY]** \| (Range: 0 - 100) \| \| **Hypertension** \| \| \| \| **36** \| Alpha blockers \| (Range: 0 - 100) \| \| **37** \| Angiotensin converting enzyme inhibitors \| (Range: 0 - 100) \| \| **38** \| Angiotensin receptor blockers \| (Range: 0 - 100) \| \| **39** \| Beta blockers \| (Range: 0 - 100) \| \| **40** \| Calcium channel blockers \| (Range: 0 - 100) \| \| **41** \| Diuretics **[FOR HUN, CZ AND GR ONLY]** \| (Range: 0 - 100) \| \| **42** \| Imidazoline-I receptor agonists **[FOR HUN AND CZ ONLY]** \| (Range: 0 - 100) \| \| **43** \| Sodium-glucose cotransporter inhibitors **[FOR ROM ONLY]** \| (Range: 0 - 100) \| \| **Dyslipidemia** \| \| \| \| **44** \| Statins \| (Range: 0 - 100) \| \| **45** \| PCSK9 inhibitors \| (Range: 0 - 100) \| \| **46** \| Bile acid binding resins \| (Range: 0 - 100) \| \| **47** \| Cholesterol absorption blockers \| (Range: 0 - 100) \| \| **48** \| Fibric acid derivatives \| (Range: 0 - 100) \| \| **49** \| Probucol (Lorelco) **[N/A FOR HUN AND CZ]** \| (Range: 0 - 100) \| \| **Atrial fibrillation** \| \| \| \| **50** \| Acetylsalicylic acid (Aspirin) \| (Range: 0 - 100) \| \| **51** \| Warfarin \| (Range: 0 - 100) \| \| **52** \| Heparin \| (Range: 0 - 100) \| \| **53** \| Low Molecular Weight Heparin (LMWH) \| (Range: 0 - 100) \| \| **54** \| NOACs/DOACs \| (Range: 0 - 100) \| \| **55** \| Antiarrhythmics \| (Range: 0 - 100) \| \| **Angina** \| \| \| \| **56** \| Aspirin \| (Range: 0 - 100) \| \| **57** \| Clopidogrel \| (Range: 0 - 100) \| \| **58** \| Ticagrelor \| (Range: 0 - 100) \| \| **59** \| Beta blockers \| (Range: 0 - 100) \| \| **60** \| Angiotensin receptor blocker \| (Range: 0 - 100) \| \| **61** \| Statins \| (Range: 0 - 100) \| \| **61.1** \| Angiotensin-converting enzyme inhibitor **[FOR GR ONLY]** \| (Range: 0 - 100) \| \| **62** \| Trimetazidine **[FOR HUN ONLY]** \| (Range: 0 - 100) \| \| **63** \| Ivabradine **[FOR HUN ONLY]** \| (Range: 0 - 100) \| \| **Chronic Kidney Disease** \| \| \| \| **64** \| Angiotensin-converting enzyme inhibitor \| (Range: 0 - 100) \| \| **65** \| Angiotensin receptor blocker \| (Range: 0 - 100) \| \| **66** \| Hypoglycemic agents for glycemic control \| (Range: 0 - 100) \| \| **67** \| Statins \| (Range: 0 - 100) \| \| **68** \| Erythropoietin/Erythropoietin stimulating agents (ESA) \| (Range: 0 - 100) \| \| **69** \| Phosphate binders \| (Range: 0 - 100) \| |
| Q3 | **ASK ALL, SC**  **PIPE IN SELECTION FROM S1**  **SHOW GRID TABLE**  **DISPLAY UNIT FOR DOSES ARE FIXED**  **DO NOT ALLOW RESPONDENT TO PROCEED IF VALUE IN COLUMN D IS NOT WITHIN RANGE INDICATED, SHOW PROMPT**  **ALLOW INPUT IN COL A, BUT DISABLE INPUT IN COL B-D IF THERE IS N/A**  **HIDE COLUMN D FOR RESPONDENTS**  **TOTAL SUM FOR COLUMN A => 100 or more**  **DO NOT ALLOW PERCENTAGE FOR INDIVIDUAL DRUG CLASSESS TO EXCEED 100%**   1. What proportion of your patients diagnosed with [insert comorbidity from S4], receive each of the following treatments for their condition in an average year?   **Please enter a percentage for each treatment/drug [NOTE FOR SCRIPTING: WHILE THE PERCENTAGE FOR EACH COMORBIDITY CAN EXCEED 100%, THE PERCENTAGE FOR EACH DRUG CLASS SHOULD BE CAPPED AT 100%]**  **DO NOT ALLOW part B/C to be entered if part A was CODED 0 for “% patients”.**   1. What is the average dose (i.e. dose of drug per application)?   **FOR section 8 - show given drop down menu for part B**  **FOR section 9, apply additional range restrictions.**  The WHO’s daily defined dose (DDD) has been added as a reference for each drug. The DDD is the assumed average maintenance dose per day for a drug used for its main indication in adults. The DDD does not necessarily reflect the recommended or Prescribed Daily Dose. Therapeutic doses for individual patients and patient groups will often differ from the DDD as they will be based on individual characteristics (such as age, weight, ethnic differences, type, and severity of disease) and pharmacokinetic considerations.  **Please enter a number for the average dose for each drug within the stated range**   1. What is the typical frequency of the treatment regimen? **[DO NOT DISPLAY DOSE MULTIPLIERS, FOR SCRIPTING PURPOSES ONLY]**    1. Three times daily **[Dose multiplier: 3]**    2. Twice daily **[Dose multiplier: 2]**    3. Once daily **[Dose multiplier: 1]**    4. Three times a week **[Dose multiplier: 0.427]**    5. Two times a week **[Dose multiplier: 0.285]**    6. Once a week **[Dose multiplier: 0.142]**    7. Every other week **[Dose multiplier: 0.0712]**    8. Monthly **[Dose multiplier: 0.0329]**   **Please select one only**  **[NOTE: PLEASE SHOW OPTIONS AS A DROP-DOWN MENU]**   \| **Treatments** \| \| \| \| --- \| --- \| --- \| \|  \|  \| **A**  **% patients receiving drug in a year** \| \| **WHO DDD** \| **B**  **Individual Dose Strength per application** \| **Display**  **unit** \| **C**  **Dose Frequency** \| **D**  **Dynamically calculated DDD**  **(Dose strength X corresponding dose multiplier based on dose frequency)** \| \|  \| **T2D** \|  \| \|  \|  \|  \|  \|  \| \| **1** \| **Metformin/biguanides** \|  \| \|  \|  \|  \|  \|  \| \| **i** \| Metformin \|  \| \| 2g \|  \| g \|  \|  \| \| **2** \| **Dipeptidyl peptidase 4 (DPPIV Inhibitors)** \|  \| \|  \|  \|  \|  \|  \| \| **i** \| Sitagliptin (Januvia) \|  \| \| 0.1g \|  \| g \|  \|  \| \| **ii** \| Saxagliptin (Onglyza) \|  \| \| 5mg \|  \| mg \|  \|  \| \| **iii** \| Linagliptin (Tradjenta) \|  \| \| 5mg \|  \| mg \|  \|  \| \| **iv** \| Alogliptin (Nesina/Vipidia) \|  \| \| 25mg \|  \| mg \|  \|  \| \| **v** \| Vildagliptin (Galvus) \|  \| \| 0.1g \|  \| g \|  \|  \| \| **3** \| **Sodium-glucose cotransporter inhibitors** \|  \| \|  \|  \|  \|  \|  \| \| **i** \| Canagliflozin (Invokana) \|  \| \| 0.2g \|  \| g \|  \|  \| \| **ii** \| Dapagliflozin (Forxiga) \|  \| \| 10mg \|  \| mg \|  \|  \| \| **iii** \| Empagliflozin (Jardiance) \|  \| \| 17.5mg \|  \| mg \|  \|  \| \| **4** \| **Neutral Protamine Hagedorn insulins** \|  \| \|  \|  \|  \|  \|  \| \| **i** \| Isophane \|  \| \| 40 U \|  \| U \|  \|  \| \| **5** \| **Short-acting insulins** \|  \| \|  \|  \|  \|  \|  \| \| **i** \| Aspart (NovoRapid/Novolog/Fiasp) \|  \| \| 40 U \|  \| U \|  \|  \| \| **ii** \| Lispro (Humalog) \|  \| \| 40 U \|  \| U \|  \|  \| \| **6** \| **Long-acting insulins** \|  \| \|  \|  \|  \|  \|  \| \| **i** \| Glargine (Lantus, Semglee, Basaglar) \|  \| \| 40 U \|  \| U \|  \|  \| \| **ii** \| Glargine (Toujeo) \|  \| \| 40 U \|  \| U \|  \|  \| \| **iii** \| Detemir (Levemir) \|  \| \| 40 U \|  \| U \|  \|  \| \| **iv** \| Degludec (Tresiba) \|  \| \| 40 U \|  \| U \|  \|  \| \| **7** \| **Pre-Mixed Insulin** \|  \| \|  \|  \|  \|  \|  \| \| **i** \| Aspart & Protamine (Novomix) \|  \| \| 40 U \|  \| U \|  \|  \| \| **ii** \| Degludec & Aspart (Ryzodeg) **[N/A FOR GR]** \|  \| \| 40 U \|  \| U \|  \|  \| \| **iii** \| 25% Lispro & 75% Lispro protamine (Humalog Mix-25) **[FOR GR ONLY]** \|  \| \| 40 U \| (Range: 1 – 60) \| U \|  \|  \| \| **iv** \| 50% Lispro & 50% Lispro protamine (Humalog Mix-50) **[FOR GR ONLY]** \|  \| \| 40 U \| (Range: 1 – 60) \| U \|  \|  \| \| **v** \| Biphasic isophane insulin (Humulin M3) 100 IU/ml **[FOR GR ONLY]** \|  \| \| 40 U \| (Range: 1 – 60) \| U \|  \|  \| \| **8** \| **GLP-1 agonists** \|  \| \|  \|  \|  \|  \|  \| \| **i** \| Dulaglutide (Trulicity) \|  \| \| 0.16mg \| 0.75 mg  1.5 mg  3.0 mg  4.5 mg \| mg \|  \|  \| \| **ii** \| Exenatide (Byetta/Bydureon) \|  \| \| 15mcg \| 5 mcg  10 mcg \| mcg \|  \|  \| \| **iii** \| Semaglutide (Ozempic) \|  \| \| 0.11mg \| 0.5 mg  1 mg \| mg \|  \|  \| \| **iv** \| Liraglutide (Victoza) \|  \| \| 1.5mg \| 1.2 mg  1.8 mg \| mg \|  \|  \| \| **v** \| Lixisenatide (Lyxumia) \|  \| \| 20mcg \| 10 mcg  20 mcg \| mcg \|  \|  \| \| **9** \| **Basal and GLP-1 fixed ratio combinations** \|  \| \|  \|  \|  \|  \|  \| \| **i** \| Degludec & Liraglutide (Xultophy)  100 units/ml, 3.6mg/ml \|  \| \| 40 U \| (Range: 10 – 50) \| U \|  \|  \| \| **ii** \| Glargine & Lixisenatide (Soliqua)  100 units/ml, 33mcg/ml **[N/A FOR GR]** \|  \| \| 40 U \| (Range: 30 – 60) \| U \|  \|  \| \| **iii** \| Glargine & Lixisenatide (Soliqua)  100 units/ml, 50mcg/ml **[N/A FOR GR]** \|  \| \| 40 U \| (Range:  10 – 40) \| U \|  \|  \| \| **10** \| **Sulfonylureas** \|  \| \|  \|  \|  \|  \|  \| \| **I** \| Gliclazide \|  \| \| 60mg \|  \| mg \|  \|  \| \| **Ii** \| Glimepiride \|  \| \| 2mg \|  \| mg \|  \|  \| \| **Iii** \| Glipizide \|  \| \| 10mg \|  \| mg \|  \|  \| \| **Iv** \| Tolbutamide **[N/A FOR GR]** \|  \| \| 1.5g \|  \| g \|  \|  \| \| **11** \| **Thiazolidinedione** \|  \| \|  \|  \|  \|  \|  \| \| **I** \| Pioglitazone \|  \| \| 30mg \|  \| mg \|  \|  \| \| **ii** \| Rosiglitazone **[N/A FOR GR]** \|  \| \| 6mg \|  \| mg \|  \|  \| \| **12** \| **Alpha-glucosidase enzyme inhibitors [FOR CZ ONLY]** \|  \| \|  \|  \|  \|  \|  \| \| **i** \| Acarbose \|  \| \| 0.3g \|  \| g \|  \|  \| \| **12.1** \| **Meglitinides [FOR GR ONLY]** \|  \| \|  \|  \|  \|  \|  \| \| **i** \| Repaglinide (NovoNorm) \|  \| \| 4mg \|  \| mg \|  \|  \| \| **ii** \| Nateglinide (Starlix) \|  \| \| 360mg \|  \| mg \|  \|  \| \|  \| **Asthma** \|  \| \|  \|  \|  \|  \|  \| \| **13** \| **B-selective agonist (short-acting)** \|  \| \|  \|  \|  \|  \|  \| \| **I** \| Albuterol/Salbutamol \|  \| \| 0.8mg \|  \| mg \|  \|  \| \| **Ii** \| Levalbuterol/Levosalbutamol \|  \| \| 0.8mg \|  \| mg \|  \|  \| \| **14** \| **B-selective agonist (long-acting)** \|  \| \|  \|  \|  \|  \|  \| \| **I** \| Salmeterol \|  \| \| 0.1mg \|  \| mg \|  \|  \| \| **Ii** \| Formoterol \|  \| \| 24mcg \|  \| mcg \|  \|  \| \| **15** \| **Muscarinic receptor agonist** \|  \| \|  \|  \|  \|  \|  \| \| **i** \| Acetylcholine \|  \| \| 120mg \|  \| mg \|  \|  \| \| **16** \| **Methylxanthine** \|  \| \|  \|  \|  \|  \|  \| \| **I** \| Teofillin/Theophylline \|  \| \| 0.4g \|  \| g \|  \|  \| \| **17** \| **Mast cell stabilizers** \|  \| \|  \|  \|  \|  \|  \| \| **I** \| Cromolyn sodium \|  \| \| 80mg \|  \| mg \|  \|  \| \| **18** \| **Corticosteroids** \|  \| \|  \|  \|  \|  \|  \| \| **I** \| Beclomethasone \|  \| \| 0.8mg \|  \| mg \|  \|  \| \| **Ii** \| Budesonide \|  \| \| 9mg \|  \| mg \|  \|  \| \| **Iii** \| Ciclesonide \|  \| \| 0.2mg \|  \| mg \|  \|  \| \| **Iv** \| Fluticasone \|  \| \| 0.6mg \|  \| mg \|  \|  \| \| **V** \| Mometasone \|  \| \| 0.2mg \|  \| mg \|  \|  \| \| **19** \| **Leukotriene synthesis inhibitors** \|  \| \|  \|  \|  \|  \|  \| \| **I** \| Zileuton (Zyflo) \|  \| \| 2400mg \|  \| mg \|  \|  \| \| **20** \| **Leukotriene receptor antagonist** \|  \| \|  \|  \|  \|  \|  \| \| **I** \| Zafirlukast \|  \| \| 40mg \|  \| mg \|  \|  \| \| **Ii** \| Montelukast \|  \| \| 10mg \|  \| mg \|  \|  \| \| **21** \| **Biologic agents** \|  \| \|  \|  \|  \|  \|  \| \| **i** \| Omalizumab (Xolair) \|  \| \| 16mg \|  \| mg \|  \|  \| \| **ii** \| Mepolizumab (Nucala) \|  \| \| 3.6mg \|  \| mg \|  \|  \| \| **iii** \| Reslizumab (Cinqair) \|  \| \| 7.1mg \|  \| mg \|  \|  \| \| **iv** \| Benralizumab (Fasenra) \|  \| \| 0.54mg \|  \| mg \|  \|  \| \| **v** \| Dupilumab (Dupixent) \|  \| \| 21.4mg \|  \| mg \|  \|  \| \|  \| **Sleep apnea** \|  \| \|  \|  \|  \|  \|  \| \| **22** \| Continuous positive airway pressure at fixed pressure \|  \| \| N/A \|  \| N/A \| N/A \| N/A \| \| **23** \| Bi-level positive airway pressure \|  \| \| N/A \|  \| N/A \| N/A \| N/A \| \| **24** \| Oral or dental appliances \|  \| \| N/A \|  \| N/A \| N/A \| N/A \| \|  \| **Osteoarthritis** \|  \| \|  \|  \|  \|  \|  \| \| **25** \| Acetaminophen/Paracetamol \|  \| \| 3g \|  \| g \|  \|  \| \| **26** \| **Nonsteroidal Anti-inflammatory Drugs (NSAIDS)** \|  \| \|  \|  \|  \|  \|  \| \| **i** \| Naproxen \|  \| \| 0.5g \|  \| g \|  \|  \| \| **ii** \| Diclofenac \|  \| \| 0.1g \|  \| g \|  \|  \| \| **iii** \| Celecoxib \|  \| \| 0.2g \|  \| g \|  \|  \| \| **iv** \| Mefenamic acid \|  \| \| 1g \|  \| g \|  \|  \| \| **v** \| Etoricoxib \|  \| \| 60mg \|  \| mg \|  \|  \| \| **vi** \| Indomethacin \|  \| \| 0.1g \|  \| g \|  \|  \| \| **vii** \| Rofecoxib \|  \| \| 25mg \|  \| mg \|  \|  \| \| **viii** \| Parecoxib \|  \| \| 40mg \|  \| mg \|  \|  \| \| **ix** \| Ibuprofen \|  \| \| 30mg \|  \| mg \|  \|  \| \| **x** \| Acetylsalicylic acid (Aspirin) \|  \| \| 3g \|  \| g \|  \|  \| \| **27** \| **Glucocorticoids** \|  \| \|  \|  \|  \|  \|  \| \| **I** \| Prednisolone \|  \| \| 10mg \|  \| mg \|  \|  \| \| **Ii** \| Methylprednisolone \|  \| \| 7.5mg \|  \| mg \|  \|  \| \| **Iii** \| Hydrocortisone \|  \| \| 30mg \|  \| mg \|  \|  \| \| **Iv** \| Betamethasone \|  \| \| 1.5mg \|  \| mg \|  \|  \| \| **V** \| Dexamethasone \|  \| \| 1.5mg \|  \| mg \|  \|  \| \| **Vi** \| Deflasacorte \|  \| \| 15mg \|  \| mg \|  \|  \| \| **Vii** \| Fluocortolon \|  \| \| 10mg \|  \| mg \|  \|  \| \| **28** \| Hyaluronic acid \|  \| \| 3.6mg \|  \| mg \|  \|  \| \| **29** \| **Opioids** \|  \| \|  \|  \|  \|  \|  \| \| **i** \| Codeine \|  \| \| 0.1g \|  \| g \|  \|  \| \| **ii** \| Hydromorphone \|  \| \| 20mg \|  \| mg \|  \|  \| \| **iii** \| Oxycodone \|  \| \| 75mg \|  \| mg \|  \|  \| \| **iv** \| Morphine \|  \| \| 0.1g \|  \| g \|  \|  \| \| **v** \| Meperidine/Pethidine \|  \| \| 0.4g \|  \| g \|  \|  \| \| **vi** \| Tramadol \|  \| \| 0.3g \|  \| g \|  \|  \| \| **30** \| Duloxetine \|  \| \| 60mg \|  \| mg \|  \|  \| \|  \| **Heart failure** \|  \| \|  \|  \|  \|  \|  \| \| **31** \| **Diuretics** \|  \| \|  \|  \|  \|  \|  \| \| **i** \| Hydrochlorothiazide \|  \| \| 25mg \|  \| mg \|  \|  \| \| **ii** \| Acetazolamide \|  \| \| 0.75g \|  \| g \|  \|  \| \| **iii** \| Methazolamide \|  \| \| 0.2g \|  \| g \|  \|  \| \| **iv** \| Spironolactone \|  \| \| 75mg \|  \| mg \|  \|  \| \| **v** \| Amiloride \|  \| \| 10mg \|  \| mg \|  \|  \| \| **vi** \| Triamterene \|  \| \| 0.1g \|  \| g \|  \|  \| \| **vii** \| Mannitol \|  \| \| 0.8g \|  \| g \|  \|  \| \| **viii** \| Eplerenone \|  \| \| 50mg \|  \| mg \|  \|  \| \| **ix** \| Indapamide \|  \| \| 2.5mg \|  \| mg \|  \|  \| \| **x** \| Furosemide \|  \| \| 40mg \|  \| mg \|  \|  \| \| **32** \| **Angiotensin converting enzyme inhibitors** \|  \| \|  \|  \|  \|  \|  \| \| **i** \| Captopril \|  \| \| 50mg \|  \| mg \|  \|  \| \| **ii** \| Kinapril \|  \| \| 15mg \|  \| mg \|  \|  \| \| **iii** \| Enalapril \|  \| \| 10mg \|  \| mg \|  \|  \| \| **iv** \| Benazepril \|  \| \| 7.5mg \|  \| mg \|  \|  \| \| **v** \| Zofenopril \|  \| \| 30mg \|  \| mg \|  \|  \| \| **vi** \| Fosinopril \|  \| \| 15mg \|  \| mg \|  \|  \| \| **vii** \| Trandolapril \|  \| \| 2mg \|  \| mg \|  \|  \| \| **viii** \| Cilazapril \|  \| \| 2.5mg \|  \| mg \|  \|  \| \| **ix** \| Lisinopril \|  \| \| 10mg \|  \| mg \|  \|  \| \| **x** \| Perindopril \|  \| \| 4mg \|  \| mg \|  \|  \| \| **xi** \| Ramipril \|  \| \| 2.5mg \|  \| mg \|  \|  \| \| **33** \| **Beta blockers** \|  \| \|  \|  \|  \|  \|  \| \| **i** \| Metoprolol \|  \| \| 0.15g \|  \| g \|  \|  \| \| **ii** \| Karvedilol \|  \| \| 37.5mg \|  \| mg \|  \|  \| \| **iii** \| Nebivolol \|  \| \| 5mg \|  \| mg \|  \|  \| \| **iv** \| Bisoprolol \|  \| \| 10mg \|  \| mg \|  \|  \| \| **v** \| Atenolol \|  \| \| 75mg \|  \| mg \|  \|  \| \| **vi** \| Pindolol \|  \| \| 15mg \|  \| mg \|  \|  \| \| **vii** \| Acebutolol \|  \| \| 0.4g \|  \| g \|  \|  \| \| **viii** \| Propranolol \|  \| \| 0.16g \|  \| g \|  \|  \| \| **34** \| **Aldosterone antagonist** \|  \| \|  \|  \|  \|  \|  \| \| **i** \| Spironolactone \|  \| \| 75mg \|  \| mg \|  \|  \| \| **ii** \| Eplerenone \|  \| \| 50mg \|  \| mg \|  \|  \| \| **35** \| **Angiotensin receptor blockers** \|  \| \|  \|  \|  \|  \|  \| \| **i** \| Valsartan \|  \| \| 80mg \|  \| mg \|  \|  \| \| **ii** \| Losartan \|  \| \| 50mg \|  \| mg \|  \|  \| \| **iii** \| Candesartan \|  \| \| 8mg \|  \| mg \|  \|  \| \| **iv** \| Irbesartan \|  \| \| 0.15g \|  \| g \|  \|  \| \| **v** \| Telmisartan \|  \| \| 40mg \|  \| mg \|  \|  \| \| **vi** \| Olmesartan \|  \| \| 20mg \|  \| mg \|  \|  \| \| **vii** \| Eprosartan \|  \| \| 0.6g \|  \| g \|  \|  \| \| **35.1** \| **Sodium-glucose cotransporter inhibitors [FOR GR ONLY]** \|  \| \|  \|  \|  \|  \|  \| \| **i** \| Canagliflozin (Invokana) \|  \| \| 0.2g \|  \| g \|  \|  \| \| **ii** \| Dapagliflozin (Forxiga) \|  \| \| 10mg \|  \| mg \|  \|  \| \| **iii** \| Empagliflozin (Jardiance) \|  \| \| 17.5mg \|  \| mg \|  \|  \| \|  \| **Hypertension** \|  \| \|  \|  \|  \|  \|  \| \| **36** \| **Alpha blockers** \|  \| \|  \|  \|  \|  \|  \| \| **I** \| Doxazosin \|  \| \| 4mg \|  \| mg \|  \|  \| \| **Ii** \| Prazosin \|  \| \| 5mg \|  \| mg \|  \|  \| \| **Iii** \| Terazosin \|  \| \| 5mg \|  \| mg \|  \|  \| \| **37** \| **Angiotensin converting enzyme inhibitors** \|  \| \|  \|  \|  \|  \|  \| \| **i** \| Captopril \|  \| \| 50mg \|  \| mg \|  \|  \| \| **ii** \| Kinapril \|  \| \| 15mg \|  \| mg \|  \|  \| \| **iii** \| Enalapril \|  \| \| 10mg \|  \| mg \|  \|  \| \| **iv** \| Benazepril \|  \| \| 7.5mg \|  \| mg \|  \|  \| \| **v** \| Zofenopril \|  \| \| 30mg \|  \| mg \|  \|  \| \| **vi** \| Fosinopril \|  \| \| 15mg \|  \| mg \|  \|  \| \| **vii** \| Trandolapril \|  \| \| 2mg \|  \| mg \|  \|  \| \| **viii** \| Cilazapril \|  \| \| 2.5mg \|  \| mg \|  \|  \| \| **ix** \| Lisinopril \|  \| \| 10mg \|  \| mg \|  \|  \| \| **x** \| Perindopril \|  \| \| 4mg \|  \| mg \|  \|  \| \| **xi** \| Ramipril \|  \| \| 2.5mg \|  \| mg \|  \|  \| \| **38** \| **Angiotensin receptor blockers** \|  \| \|  \|  \|  \|  \|  \| \| **i** \| Valsartan \|  \| \| 80mg \|  \| mg \|  \|  \| \| **ii** \| Losartan \|  \| \| 50mg \|  \| mg \|  \|  \| \| **iii** \| Candesartan \|  \| \| 8mg \|  \| mg \|  \|  \| \| **iv** \| Irbesartan \|  \| \| 0.15g \|  \| g \|  \|  \| \| **v** \| Telmisartan \|  \| \| 40mg \|  \| mg \|  \|  \| \| **vi** \| Olmesartan \|  \| \| 20mg \|  \| mg \|  \|  \| \| **vii** \| Eprosartan \|  \| \| 0.6g \|  \| g \|  \|  \| \| **39** \| **Beta blockers** \|  \| \|  \|  \|  \|  \|  \| \| **i** \| Metoprolol \|  \| \| 0.15g \|  \| g \|  \|  \| \| **ii** \| Karvedilol \|  \| \| 37.5mg \|  \| mg \|  \|  \| \| **iii** \| Nebivolol \|  \| \| 5mg \|  \| mg \|  \|  \| \| **iv** \| Bisoprolol \|  \| \| 10mg \|  \| mg \|  \|  \| \| **v** \| Atenolol \|  \| \| 75mg \|  \| mg \|  \|  \| \| **vi** \| Pindolol \|  \| \| 15mg \|  \| mg \|  \|  \| \| **vii** \| Acebutolol \|  \| \| 0.4g \|  \| g \|  \|  \| \| **viii** \| Propranolol \|  \| \| 0.16g \|  \| g \|  \|  \| \| **40** \| **Calcium channel blockers** \|  \| \|  \|  \|  \|  \|  \| \| **i** \| Amlodipin \|  \| \| 5mg \|  \| mg \|  \|  \| \| **ii** \| Benidipine \|  \| \| 4mg \|  \| mg \|  \|  \| \| **iii** \| Felodipin \|  \| \| 5mg \|  \| mg \|  \|  \| \| **iv** \| Lacidipine \|  \| \| 4mg \|  \| mg \|  \|  \| \| **v** \| Lercanidipine \|  \| \| 10mg \|  \| mg \|  \|  \| \| **vi** \| Nifedipin \|  \| \| 30mg \|  \| mg \|  \|  \| \| **vii** \| Nimodipin \|  \| \| 0.3g \|  \| g \|  \|  \| \| **viii** \| Diltiazem \|  \| \| 0.24g \|  \| g \|  \|  \| \| **ix** \| Verapamil \|  \| \| 0.24g \|  \| g \|  \|  \| \| **41** \| **Diuretics [FOR HUN, CZ AND GR ONLY]** \|  \| \|  \|  \|  \|  \|  \| \| **i** \| Hydrochlorothiazide \|  \| \| 25mg \|  \| mg \|  \|  \| \| **ii** \| Acetazolamide \|  \| \| 0.75g \|  \| g \|  \|  \| \| **iii** \| Methazolamide \|  \| \| 0.2g \|  \| g \|  \|  \| \| **iv** \| Spironolactone \|  \| \| 75mg \|  \| mg \|  \|  \| \| **v** \| Amiloride \|  \| \| 10mg \|  \| mg \|  \|  \| \| **vi** \| Triamterene \|  \| \| 0.1g \|  \| g \|  \|  \| \| **vii** \| Mannitol \|  \| \| 0.8g \|  \| g \|  \|  \| \| **viii** \| Eplerenone \|  \| \| 50mg \|  \| mg \|  \|  \| \| **ix** \| Indapamide \|  \| \| 2.5mg \|  \| mg \|  \|  \| \| **x** \| Furosemide \|  \| \| 40mg \|  \| mg \|  \|  \| \| **42** \| **Imidazoline-I receptor agonists [FOR HUN AND CZ ONLY]** \|  \| \|  \|  \|  \|  \|  \| \| **i** \| Clonidine \|  \| \| 0.45mg \|  \| mg \|  \|  \| \| **ii** \| Guanfacine \|  \| \| 3mg \|  \| mg \|  \|  \| \| **iii** \| Moxonidine \|  \| \| 0.3mg \|  \| mg \|  \|  \| \| **iv** \| Rilmenidine \|  \| \| 1mg \|  \| mg \|  \|  \| \| **v** \| Tolonidine \|  \| \| 0.75mg \|  \| mg \|  \|  \| \| **43** \| **Sodium-glucose cotransporter inhibitors [FOR ROM ONLY]** \|  \| \|  \|  \|  \|  \|  \| \| **i** \| Canagliflozin (Invokana) \|  \| \| 0.2g \|  \| g \|  \|  \| \| **ii** \| Dapagliflozin (Forxiga) \|  \| \| 10mg \|  \| mg \|  \|  \| \| **iii** \| Empagliflozin (Jardiance) \|  \| \| 17.5mg \|  \| mg \|  \|  \| \|  \| **Dyslipidemia** \|  \| \|  \|  \|  \|  \|  \| \| **44** \| **Statins** \|  \| \|  \|  \|  \|  \|  \| \| **i** \| Atorvastatin \|  \| \| 20mg \|  \| mg \|  \|  \| \| **ii** \| Fluvastatin \|  \| \| 60mg \|  \| mg \|  \|  \| \| **iii** \| Lovastatin \|  \| \| 45mg \|  \| mg \|  \|  \| \| **iv** \| Pitavastatin \|  \| \| 2mg \|  \| mg \|  \|  \| \| **v** \| Pravastatin \|  \| \| 30mg \|  \| mg \|  \|  \| \| **vi** \| Rosuvastatin \|  \| \| 10mg \|  \| mg \|  \|  \| \| **vii** \| Simvastatin \|  \| \| 30mg \|  \| mg \|  \|  \| \| **45** \| **PCSK9 inhibitors** \|  \| \|  \|  \|  \|  \|  \| \| **i** \| Alirocumab (Praluent) \|  \| \| 5.4mg \|  \| mg \|  \|  \| \| **ii** \| Evolocumab (Repatha) \|  \| \| 10mg \|  \| mg \|  \|  \| \| **46** \| **Bile acid binding resins** \|  \| \|  \|  \|  \|  \|  \| \| **i** \| Cholestyramine resin \|  \| \| 14g \|  \| g \|  \|  \| \| **ii** \| Colestipol hydrochloride \|  \| \| 20g \|  \| g \|  \|  \| \| **47** \| **Cholesterol absorption blockers** \|  \| \|  \|  \|  \|  \|  \| \| **i** \| Ezetimibe \|  \| \| 10mg \|  \| mg \|  \|  \| \| **48** \| **Fibric acid derivatives** \|  \| \|  \|  \|  \|  \|  \| \| **i** \| Clofibrate \|  \| \| 2g \|  \| g \|  \|  \| \| **ii** \| Gemfibrozil \|  \| \| 1.2g \|  \| g \|  \|  \| \| **iii** \| Bezafibrate \|  \| \| 0.6g \|  \| g \|  \|  \| \| **iv** \| Ciprofibrate \|  \| \| 0.1g \|  \| g \|  \|  \| \| **v** \| Fenofibrate \|  \| \| 0.2g \|  \| g \|  \|  \| \| **49** \| Probucol (Lorelco) **[N/A FOR HUN AND CZ]** \|  \| \| 500mg \|  \| mg \|  \|  \| \|  \| **Atrial fibrillation** \|  \| \|  \|  \|  \|  \|  \| \| **50** \| Acetylsalicylic acid (Aspirin) \|  \| \| 3g \|  \| g \|  \|  \| \| **51** \| Warfarin \|  \| \| 7.5mg \|  \| mg \|  \|  \| \| **52** \| Heparin \|  \| \| 10TU \|  \| TU \|  \|  \| \| **53** \| **Low Molecular Weight Heparin (LMWH)** \|  \| \|  \|  \|  \|  \|  \| \| **I** \| Enoxaparin \|  \| \| 2TU \|  \| TU \|  \|  \| \| **Ii** \| Dalteparin \|  \| \| 2.5TU \|  \| TU \|  \|  \| \| **54** \| **NOACs/DOACs** \|  \| \|  \|  \|  \|  \|  \| \| **i** \| Rivaroxaban \|  \| \| 20mg \|  \| mg \|  \|  \| \| **ii** \| Apixaban \|  \| \| 10mg \|  \| mg \|  \|  \| \| **iii** \| Edoxaban \|  \| \| 60mg \|  \| mg \|  \|  \| \| **iv** \| Dabigatran \|  \| \| 0.3g \|  \| g \|  \|  \| \| **55** \| **Antiarrhythmics** \|  \| \|  \|  \|  \|  \|  \| \| **I** \| Amiodarone \|  \| \| 0.2g \|  \| g \|  \|  \| \| **Ii** \| Flecainide \|  \| \| 0.2g \|  \| g \|  \|  \| \| **Iii** \| Lidocaine \|  \| \| 3g \|  \| g \|  \|  \| \|  \| **Angina** \|  \| \|  \|  \|  \|  \|  \| \| **56** \| Aspirin \|  \| \| 3g \|  \| g \|  \|  \| \| **57** \| Clopidogrel \|  \| \| 75mg \|  \| mg \|  \|  \| \| **58** \| Ticagrelor \|  \| \| 0.18g \|  \| g \|  \|  \| \| **59** \| **Beta blockers** \|  \| \|  \|  \|  \|  \|  \| \| **i** \| Metoprolol \|  \| \| 0.15g \|  \| g \|  \|  \| \| **ii** \| Karvedilol \|  \| \| 37.5mg \|  \| mg \|  \|  \| \| **iii** \| Nebivolol \|  \| \| 5mg \|  \| mg \|  \|  \| \| **iv** \| Bisoprolol \|  \| \| 10mg \|  \| mg \|  \|  \| \| **v** \| Atenolol \|  \| \| 75mg \|  \| mg \|  \|  \| \| **vi** \| Pindolol \|  \| \| 15mg \|  \| mg \|  \|  \| \| **vii** \| Acebutolol \|  \| \| 0.4g \|  \| g \|  \|  \| \| **viii** \| Propranolol \|  \| \| 0.16g \|  \| g \|  \|  \| \| **60** \| **Angiotensin receptor blocker** \|  \| \|  \|  \|  \|  \|  \| \| **i** \| Valsartan \|  \| \| 80mg \|  \| mg \|  \|  \| \| **ii** \| Losartan \|  \| \| 50mg \|  \| mg \|  \|  \| \| **iii** \| Candesartan \|  \| \| 8mg \|  \| mg \|  \|  \| \| **iv** \| Irbesartan \|  \| \| 0.15g \|  \| g \|  \|  \| \| **v** \| Telmisartan \|  \| \| 40mg \|  \| mg \|  \|  \| \| **vi** \| Olmesartan \|  \| \| 20mg \|  \| mg \|  \|  \| \| **vii** \| Eprosartan \|  \| \| 0.6g \|  \| g \|  \|  \| \| **61** \| **Statins** \|  \| \|  \|  \|  \|  \|  \| \| **i** \| Atorvastatin \|  \| \| 20mg \|  \| mg \|  \|  \| \| **ii** \| Fluvastatin \|  \| \| 60mg \|  \| mg \|  \|  \| \| **iii** \| Lovastatin \|  \| \| 45mg \|  \| mg \|  \|  \| \| **iv** \| Pitavastatin \|  \| \| 2mg \|  \| mg \|  \|  \| \| **v** \| Pravastatin \|  \| \| 30mg \|  \| mg \|  \|  \| \| **vi** \| Rosuvastatin \|  \| \| 10mg \|  \| mg \|  \|  \| \| **vii** \| Simvastatin \|  \| \| 30mg \|  \| mg \|  \|  \| \| **61.1** \| **Angiotensin-converting enzyme inhibitor [FOR GR ONLY]** \|  \| \|  \|  \|  \|  \|  \| \| **i** \| Captopril \|  \| \| 50mg \|  \| mg \|  \|  \| \| **ii** \| Quinapril \|  \| \| 15mg \|  \| mg \|  \|  \| \| **iii** \| Enalapril \|  \| \| 10mg \|  \| mg \|  \|  \| \| **iv** \| Benazepril \|  \| \| 7.5mg \|  \| mg \|  \|  \| \| **v** \| Zofenopril \|  \| \| 30mg \|  \| mg \|  \|  \| \| **vi** \| Fosinopril \|  \| \| 15mg \|  \| mg \|  \|  \| \| **vii** \| Trandolapril \|  \| \| 2mg \|  \| mg \|  \|  \| \| **viii** \| Cilazapril \|  \| \| 2.5mg \|  \| mg \|  \|  \| \| **ix** \| Lisinopril \|  \| \| 10mg \|  \| mg \|  \|  \| \| **x** \| Perindopril \|  \| \| 4mg \|  \| mg \|  \|  \| \| **xi** \| Ramipril \|  \| \| 2.5mg \|  \| mg \|  \|  \| \| **62** \| Trimetazidine **[FOR HUN ONLY]** \|  \| \| 40mg \|  \| mg \|  \|  \| \| **63** \| Ivabradine **[FOR HUN ONLY]** \|  \| \| 10mg \|  \| mg \|  \|  \| \|  \| **Chronic Kidney Disease** \|  \| \|  \|  \|  \|  \|  \| \| **64** \| **Angiotensin-converting enzyme inhibitor** \|  \| \|  \|  \|  \|  \|  \| \| **i** \| Captopril \|  \| \| 50mg \|  \| mg \|  \|  \| \| **ii** \| Kinapril \|  \| \| 15mg \|  \| mg \|  \|  \| \| **iii** \| Enalapril \|  \| \| 10mg \|  \| mg \|  \|  \| \| **iv** \| Benazepril \|  \| \| 7.5mg \|  \| mg \|  \|  \| \| **v** \| Zofenopril \|  \| \| 30mg \|  \| mg \|  \|  \| \| **vi** \| Fosinopril \|  \| \| 15mg \|  \| mg \|  \|  \| \| **vii** \| Trandolapril \|  \| \| 2mg \|  \| mg \|  \|  \| \| **viii** \| Cilazapril \|  \| \| 2.5mg \|  \| mg \|  \|  \| \| **ix** \| Lisinopril \|  \| \| 10mg \|  \| mg \|  \|  \| \| **x** \| Perindopril \|  \| \| 4mg \|  \| mg \|  \|  \| \| **xi** \| Ramipril \|  \| \| 2.5mg \|  \| mg \|  \|  \| \| **65** \| **Angiotensin receptor blocker** \|  \| \|  \|  \|  \|  \|  \| \| **i** \| Valsartan \|  \| \| 80mg \|  \| mg \|  \|  \| \| **ii** \| Losartan \|  \| \| 50mg \|  \| mg \|  \|  \| \| **iii** \| Candesartan \|  \| \| 8mg \|  \| mg \|  \|  \| \| **iv** \| Irbesartan \|  \| \| 0.15g \|  \| g \|  \|  \| \| **v** \| Telmisartan \|  \| \| 40mg \|  \| mg \|  \|  \| \| **vi** \| Olmesartan \|  \| \| 20mg \|  \| mg \|  \|  \| \| **vii** \| Eprosartan \|  \| \| 0.6g \|  \| g \|  \|  \| \| **66** \| **Hypoglycemic agents for glycemic control** \|  \| \|  \|  \|  \|  \|  \| \| **i** \| Repaglinide **[N/A FOR GR]** \|  \| \| 4mg \|  \| mg \|  \|  \| \| **ii** \| Nateglinide **[N/A FOR GR]** \|  \| \| 0.36g \|  \| g \|  \|  \| \| **iii** \| Rosiglitazone **[N/A FOR GR]** \|  \| \| 6mg \|  \| mg \|  \|  \| \| **iv** \| Pioglitazone **[N/A FOR GR]** \|  \| \| 30mg \|  \| mg \|  \|  \| \| **v** \| Sitagliptin (Januvia) \|  \| \| 0.1g \|  \| g \|  \|  \| \| **vi** \| Saxagliptin (Onglyza) \|  \| \| 5mg \|  \| mg \|  \|  \| \| **vii** \| Linagliptin (Trajenta) \|  \| \| 5mg \|  \| mg \|  \|  \| \| **viii** \| Alogliptin (Vipidia) \|  \| \| 25mg \|  \| mg \|  \|  \| \| **ix** \| Vildagliptin (Galvus) \|  \| \| 0.1g \|  \| g \|  \|  \| \| **x** \| Dulaglutide (Trulicity) \|  \| \| 0.16mg \| 0.75 mg  1.5 mg  3.0 mg  4.5 mg \| mg \|  \|  \| \| **xi** \| Exenatide (Byetta/Bydureon) \|  \| \| 15mcg \| 5 mcg  10 mcg \| mcg \|  \|  \| \| **xii** \| Semaglutide (Ozempic) \|  \| \| 0.11mg \| 0.5 mg  1 mg \| mg \|  \|  \| \| **xiii** \| Liraglutide (Victoza) \|  \| \| 1.5mg \| 1.2 mg  1.8 mg \| mg \|  \|  \| \| **xiv** \| Lixisenatide (Lyxumia) \|  \| \| 20mcg \| 10 mcg  20 mcg \| mcg \|  \|  \| \| **xv** \| Canagliflozin (Invokana) \|  \| \| 0.2g \|  \| g \|  \|  \| \| **xvi** \| Dapagliflozin (Forxiga) \|  \| \| 10mg \|  \| mg \|  \|  \| \| **xvii** \| Empagliflozin (Jardiance) \|  \| \| 17.5mg \|  \| mg \|  \|  \| \| **67** \| **Statins** \|  \| \|  \|  \|  \|  \|  \| \| **I** \| Atorvastatin \|  \| \| 20mg \|  \| mg \|  \|  \| \| **Ii** \| Fluvastatin \|  \| \| 60mg \|  \| mg \|  \|  \| \| **Iii** \| Lovastatin \|  \| \| 45mg \|  \| mg \|  \|  \| \| **Iv** \| Pitavastatin \|  \| \| 2mg \|  \| mg \|  \|  \| \| **V** \| Pravastatin \|  \| \| 30mg \|  \| mg \|  \|  \| \| **Vi** \| Rosuvastatin \|  \| \| 10mg \|  \| mg \|  \|  \| \| **Vii** \| Simvastatin \|  \| \| 30mg \|  \| mg \|  \|  \| \| **68** \| Erythropoietin**/Erythropoietin stimulating agents (ESA)** \|  \| \|  \|  \|  \|  \|  \| \| **i** \| Darbepoetin alfa \|  \| \| 4.5mcg \|  \| mcg \|  \|  \| \| **ii** \| Epoetin alfa \|  \| \| 2500 IU \|  \| IU \|  \|  \| \| **iii** \| Epoetin beta \|  \| \| 1500 IU \|  \| IU \|  \|  \| \| **iv** \| Methoxy polyethylene glycol-epoetin beta \|  \| \| 4mcg \|  \| mcg \|  \|  \| \| **v** \| Epoetin zeta \|  \| \| 3000 IU \|  \| IU \|  \|  \| \| **69** \| **Phosphate binders** \|  \| \|  \|  \|  \|  \|  \| \| **i** \| Sucroferric oxyhydroxide \|  \| \| 1.5g \|  \| g \|  \|  \| \| **ii** \| Sevelamer \|  \| \| 6.4g \|  \| g \|  \|  \| \| **iii** \| Ferric citrate \|  \| \| 6g \|  \| g \|  \|  \| \| **iv** \| Lanthanum carbonate \|  \| \| 2.25g \|  \| g \|  \|  \| \| **v** \| Calcium acetate \|  \| \| 6g \|  \| g \|  \|  \| \| **70** \| Hemodialysis \|  \| \| N/A \|  \| N/A \| N/A \| N/A \| \| **71** \| Peritoneal dialysis \|  \| \| N/A \|  \| N/A \| N/A \| N/A \| |
| Q3e | **ASK ALL, RANGE 0-100, TOTAL =100%**  **DYNAMICALLY CALCULATE INPUTS SO THAT TOTAL = 100%**  **PIPE IN SELECTION FROM S1**  **SHOW GRID TABLE**   1. Of your [insert comorbidity from S4] patients receiving treatment for their condition, what proportion receive brand vs. generic treatment?   **Please enter a percentage for each treatment within the stated range**   \|  \|  \| **Percentage** \| \| --- \| --- \| --- \| \| **1** \| Brand treatment \| % \| \| **2** \| Generic treatment \| % \| \|  \|  \| 100% \| |
| Q4 | **PIPE IN SELECTION FROM S1**  **SHOW GRID TABLE WITH PRE-FILLED RANGES, ALLOW RESPONDENTS TO OVERWRITE ON CELLS**  **SHOW PROMPT IF INPUT IS OUT OF RANGE SPECIFIED**  **DO NOT ALLOW part B to be entered if part A was CODED 0 for “% patients”**  **TOTAL SUM FOR COLUMN A => 100 or more**  **SKIP FOR HEART FAILURE, DYSLIPIDEMIA & ANGINA**   1. What proportion of your [insert comorbidity from S4] patients receive each of the following consumables/devices for their condition?   **Please enter a percentage for each within the stated range**   1. Of those [insert comorbidity from S4] patients receiving a consumable/device, how many of each consumable/device does a typical patient use in a year?   **Please enter a number for each within the stated range**   \|  \| **Consumables/devices** \| \| --- \| --- \| \|  \|  \| **% of patients**  **(range in brackets)** \| **Number used in 1 year**  **(range in brackets)** \| \|  \| **T2D** \|  \|  \| \| **1** \| Insulin pumps \| (Range: 0 – 100) \| (Range: 1 – 12) \| \| **2** \| Glucometer \| (Range: 0 – 100) \| (Range: 1 – 12) \| \| **3** \| Glucose strips \| (Range: 0 – 100) \| (Range: 1 – 1500) \| \| **4** \| Flash glucose monitoring device (e.g. Freestyle libre) \| (Range: 0 – 100) \| (Range: 1 – 12) \| \|  \| **Asthma** \|  \|  \| \| **5** \| Pressured metered dose inhaler \| (Range: 0 – 100) \| (Range: 1 – 24) \| \| **6** \| Dry powder inhaler \| (Range: 0 – 100) \| (Range: 1 – 24) \| \| **7** \| Small volume nebulizer \| (Range: 0 – 100) \| (Range: 1 – 12) \| \| **8** \| Metered dose inhaler with spacer \| (Range: 0 – 100) \| (Range: 1 – 24) \| \| **9** \| Spacer \| (Range: 0 – 100) \| (Range: 1 – 12) \| \|  \| **Sleep apnea** \|  \|  \| \| **10** \| Continuous positive airway pressure at fixed pressure device \| (Range: 0 – 100) \| (Range: 1 – 12) \| \| **11** \| Bi-level positive airway pressure device \| (Range: 0 – 100) \| (Range: 1 – 12) \| \| **12** \| Oral appliances \| (Range: 0 – 100) \| (Range: 1 – 12) \| \| **13** \| Dental appliances \| (Range: 0 – 100) \| (Range: 1 – 12) \| \|  \| **Osteoarthritis** \|  \|  \| \| **14** \| Unloader knee braces \| (Range: 0 – 100) \| (Range: 1 – 12) \| \| **15** \| Hand splints \| (Range: 0 – 100) \| (Range: 1 – 12) \| \| **16** \| Soft cervical collars \| (Range: 0 – 100) \| (Range: 1 – 12) \| \| **17** \| Lumbosacral corsets \| (Range: 0 – 100) \| (Range: 1 – 12) \| \|  \| **Hypertension** \|  \|  \| \| **18** \| Sphygmomanometer \| (Range: 0 – 100) \| (Range: 1 – 12) \| \|  \| **Atrial Fibrillation** \|  \|  \| \| **19** \| Sphygmomanometer \| (Range: 0 – 100) \| (Range: 1 – 12) \| \| **20** \| Holter monitor \| (Range: 0 – 100) \| (Range: 1 – 12) \| \| **21** \| Trans-telephonic ECG device \| (Range: 0 – 100) \| (Range: 1 – 12) \| \| **22** \| Internal loop recorder \| (Range: 0 – 100) \| (Range: 1 – 12) \| \| **23** \| External loop recorder \| (Range: 0 – 100) \| (Range: 1 – 12) \| \| **24** \| Insertable cardiac monitor \| (Range: 0 – 100) \| (Range: 1 – 12) \| \|  \| **Chronic Kidney Disease** \|  \|  \| \| **25** \| Portable hemodialysis machine \| (Range: 0 – 100) \| (Range: 1 – 12) \| \| **26** \| Portable peritoneal dialysis machine \| (Range: 0 – 100) \| (Range: 1 – 12) \| \| **27** \| Sphygmomanometer \| (Range: 0 – 100) \| (Range: 1 – 12) \| |
| Q5 | **PIPE IN SELECTION FROM S1**  **SHOW GRID TABLE WITH PRE-FILLED RANGES, ALLOW RESPONDENTS TO OVERWRITE ON CELLS**  **SHOW PROMPT IF INPUT IS OUT OF RANGE SPECIFIED**  **DO NOT ALLOW part B to be entered if part A was CODED 0 for “% patients”**  **TOTAL SUM FOR COLUMN A => 100 or more**   1. What proportion of your patients with [insert comorbidity from S4] undergo each of the following tests as part of routine monitoring of their condition?   **Please enter a percentage for each within the stated range**   1. Of those [insert comorbidity from S4] patients undergoing each of the following tests as part of routine monitoring, how many times will a typical patient undergo each test for routine monitoring in a year?   **Please enter a number for each within the stated range**   \|  \| **Monitoring tests** \| \| --- \| --- \| \|  \|  \| **% patients** \| **Number of tests in 1 year**  **(range in brackets)** \| \|  \| **T2D** \|  \|  \| \| **1** \| Fasting Plasma Glucose \| (Range: 0 – 100) \| (Range: 1 – 1500) \| \| **2** \| Random Plasma Glucose \| (Range: 0 – 100) \| (Range: 1 – 1500) \| \| **3** \| Two-hour Oral Glucose Tolerance Test \| (Range: 0 – 100) \| (Range: 1 – 1500) \| \| **4** \| Glycated hemoglobin (HbA1C) \| (Range: 0 – 100) \| (Range: 1 – 12) \| \| **5** \| Fundoscopy for diabetic neuropathy \| (Range: 0 – 100) \| (Range: 1 – 12) \| \| **6** \| Ultrasound for diabetic neuropathy \| (Range: 0 – 100) \| (Range: 1 – 12) \| \| **7** \| Fundoscopy for diabetic retinopathy **[FOR ROM ONLY]** \| (Range: 0 – 100) \| (Range: 1 – 12) \| \| **8** \| Ultrasound for peripheral artery **[FOR ROM ONLY]** \| (Range: 0 – 100) \| (Range: 1 – 12) \| \| **9** \| Lipid panel **[FOR ROM ONLY]** \| (Range: 0 – 100) \| (Range: 1 – 12) \| \| **10** \| Ankle-brachial pressure index/ankle-brachial index **[FOR ROM ONLY]** \| (Range: 0 – 100) \| (Range: 1 – 12) \| \| **11** \| Albuminuria test **[FOR ROM ONLY]** \| (Range: 0 – 100) \| (Range: 1 – 12) \| \| **11.1** \| UACR measurement for diabetic retinopathy **[FOR GR ONLY]** \| (Range: 0 – 100) \| (Range: 1 – 12) \| \| **11.2** \| Electromyogram for diabetic neuropathy **[FOR GR ONLY]** \| (Range: 0 – 100) \| (Range: 1 – 12) \| \| **11.3** \| Lipid profile for diabetic dyslipidemia **[FOR GR ONLY]** \| (Range: 0 – 100) \| (Range: 1 – 12) \| \| **11.4** \| eGFR **[FOR GR ONLY]** \| (Range: 0 – 100) \| (Range: 1 – 12) \| \|  \| **Asthma** \|  \|  \| \| **12** \| Spirometry \| (Range: 0 – 100) \| (Range: 1 – 12) \| \| **13** \| Chest X-ray \| (Range: 0 – 100) \| (Range: 1 – 4) \| \| **14** \| Peripheral eosinophilia and elevated IgE level \| (Range: 0 – 100) \| (Range: 1 – 12) \| \| **15** \| Exhaled nitric oxide **[N/A FOR HUN]** \| (Range: 0 – 100) \| (Range: 1 – 12) \| \| **16** \| Skin prick and radioallergosorbent test (RAST) \| (Range: 0 – 100) \| (Range: 1 – 12) \| \|  \| **Sleep apnea** \|  \|  \| \| **17** \| Attended, in-laboratory polysomnography \| (Range: 0 – 100) \| (Range: 1 – 12) \| \| **18** \| Portable monitoring (1 day) \| (Range: 0 – 100) \| (Range: 1 – 12) \| \| **19** \| Overnight pulse oximetry (1 night) \| (Range: 0 – 100) \| (Range: 1 – 12) \| \| **20** \| Portable monitoring of cardiopulmonary channels (1 day) \| (Range: 0 – 100) \| (Range: 1 – 12) \| \|  \| **Osteoarthritis** \|  \|  \| \| **21** \| X-ray \| (Range: 0 – 100) \| (Range: 1 – 4) \| \| **22** \| MRI / Magnetic resonance imaging \| (Range: 0 – 100) \| (Range: 1 – 4) \| \| **23** \| Complete blood count \| (Range: 0 – 100) \| (Range: 1 – 12) \| \| **24** \| Erythrocyte sedimentation rate \| (Range: 0 – 100) \| (Range: 1 – 12) \| \| **25** \| Rheumatoid factor \| (Range: 0 – 100) \| (Range: 1 – 12) \| \| **26** \| ANA/ Antinuclear antibodies \| (Range: 0 – 100) \| (Range: 1 – 12) \| \| **27** \| Synovial fluid analysis \| (Range: 0 – 100) \| (Range: 1 – 12) \| \|  \| **Heart failure** \|  \|  \| \| **28** \| ECG / Electrocardiogram \| (Range: 0 – 100) \| (Range: 1 – 12) \| \| **29** \| Bedside echocardiogram \| (Range: 0 – 100) \| (Range: 1 – 12) \| \| **30** \| Chest X-ray \| (Range: 0 – 100) \| (Range: 1 – 4) \| \| **31** \| Complete blood count \| (Range: 0 – 100) \| (Range: 1 – 12) \| \| **32** \| Serum electrolytes \| (Range: 0 – 100) \| (Range: 1 – 12) \| \| **33** \| Renal function test \| (Range: 0 – 100) \| (Range: 1 – 12) \| \| **34** \| Liver function test \| (Range: 0 – 100) \| (Range: 1 – 12) \| \| **35** \| Urinalysis \| (Range: 0 – 100) \| (Range: 1 – 12) \| \| **36** \| Fasting plasma glucose test \| (Range: 0 – 100) \| (Range: 1 – 1500) \| \| **37** \| Oral glucose tolerance test \| (Range: 0 – 100) \| (Range: 1 – 12) \| \| **38** \| Lipid panel \| (Range: 0 – 100) \| (Range: 1 – 12) \| \| **39** \| Thyroid hormone determination \| (Range: 0 – 100) \| (Range: 1 – 12) \| \| **40** \| B-type natriuretic peptide(BNP)/ N-terminal pro B-type natriuretic peptide (NT-proBNP) \| (Range: 0 – 100) \| (Range: 1 – 12) \| \| **41** \| HbA1c **[FOR ROM ONLY]** \| (Range: 0 – 100) \| (Range: 1 – 12) \| \|  \| **Hypertension** \|  \|  \| \| **42** \| Fasting plasma glucose \| (Range: 0 – 100) \| (Range: 1 – 1500) \| \| **43** \| Serum total cholesterol, low-density lipoprotein cholesterol, high-density lipoprotein cholesterol \| (Range: 0 – 100) \| (Range: 1 – 12) \| \| **44** \| Fasting serum triglycerides \| (Range: 0 – 100) \| (Range: 1 – 12) \| \| **45** \| Serum potassium and sodium \| (Range: 0 – 100) \| (Range: 1 – 12) \| \| **46** \| Serum uric acid \| (Range: 0 – 100) \| (Range: 1 – 12) \| \| **47** \| Serum creatinine \| (Range: 0 – 100) \| (Range: 1 – 12) \| \| **48** \| Estimated glomerular filtration rate (eGFR) \| (Range: 0 – 100) \| (Range: 1 – 12) \| \| **49** \| Urine analysis including a test for microalbuminuria \| (Range: 0 – 100) \| (Range: 1 – 12) \| \| **50** \| 12-lead ECG \| (Range: 0 – 100) \| (Range: 1 – 12) \| \|  \| **Dyslipidemia** \|  \|  \| \| **51** \| Lipid panel \| (Range: 0 – 100) \| (Range: 1 – 12) \| \| **52** \| Fasting plasma glucose \| (Range: 0 – 100) \| (Range: 1 – 1500) \| \| **53** \| HbA1C \| (Range: 0 – 100) \| (Range: 1 – 12) \| \| **54** \| Liver enzymes \| (Range: 0 – 100) \| (Range: 1 – 12) \| \| **55** \| BUN, Creatinine, GFR \| (Range: 0 – 100) \| (Range: 1 – 12) \| \| **56** \| Creatine phosphokinase \| (Range: 0 – 100) \| (Range: 1 – 12) \| \| **57** \| ECG / Electrocardiogram \| (Range: 0 – 100) \| (Range: 1 – 12) \| \|  \| **Atrial Fibrillation** \|  \|  \| \| **58** \| 12-L ECG \| (Range: 0 – 100) \| (Range: 1 – 12) \| \| **59** \| Complete blood count \| (Range: 0 – 100) \| (Range: 1 – 12) \| \| **60** \| Metabolic panel \| (Range: 0 – 100) \| (Range: 1 – 12) \| \| **61** \| Thyroid function test \| (Range: 0 – 100) \| (Range: 1 – 12) \| \| **62** \| Chest X-ray \| (Range: 0 – 100) \| (Range: 1 – 12) \| \| **63** \| Spiral CT scan **[N/A FOR HUN & ROM]** \| (Range: 0 – 100) \| (Range: 1 – 12) \| \| **64** \| Transesophageal echocardiogram \| (Range: 0 – 100) \| (Range: 1 – 12) \| \| **65** \| Transthoracic echocardiogram \| (Range: 0 – 100) \| (Range: 1 – 12) \| \| **66** \| Cardiac catheterization **[N/A FOR HUN]** \| (Range: 0 – 100) \| (Range: 1 – 12) \| \| **67** \| Prothrombin test \| (Range: 0 – 100) \| (Range: 1 – 12) \| \|  \| **Angina** \|  \|  \| \| **68** \| Complete blood count \| (Range: 0 – 100) \| (Range: 1 – 12) \| \| **69** \| Metabolic panel \| (Range: 0 – 100) \| (Range: 1 – 12) \| \| **70** \| Urinalysis \| (Range: 0 – 100) \| (Range: 1 – 12) \| \| **71** \| Chest X-ray \| (Range: 0 – 100) \| (Range: 1 – 4) \| \| **72** \| 12-L ECG \| (Range: 0 – 100) \| (Range: 1 – 12) \| \| **73** \| Cardiac biomarker test \| (Range: 0 – 100) \| (Range: 1 – 12) \| \| **74** \| Coronary angiography \| (Range: 0 – 100) \| (Range: 1 – 4) \| \|  \| **Chronic Kidney Disease** \|  \|  \| \| **75** \| Complete blood count \| (Range: 0 – 100) \| (Range: 1 – 12) \| \| **76** \| Metabolic panel \| (Range: 0 – 100) \| (Range: 1 – 12) \| \| **77** \| Lipid panel \| (Range: 0 – 100) \| (Range: 1 – 12) \| \| **78** \| Urinalysis \| (Range: 0 – 100) \| (Range: 1 – 12) \| \| **79** \| Kidney biopsy \| (Range: 0 – 100) \| (Range: 1 – 4) \| \| **80** \| Renal ultrasound \| (Range: 0 – 100) \| (Range: 1 – 4) \| \| **81** \| Serum calcium \| (Range: 0 – 100) \| (Range: 1 – 12) \| |

| **C: ADVERSE EVENT HCRU** |
| --- |

| Q7 | **PIPE IN SELECTION FROM S1**  **SHOW GRID TABLE WITH PRE-FILLED RANGES, ALLOW RESPONDENTS TO OVERWRITE ON CELLS**  **SHOW PROMPT IF INPUT IS OUT OF RANGE SPECIFIED**  **DO NOT ALLOW COLUMN B - F to be entered if COLUMN A was CODED 0 for “% patients”**  **IF THERE IS AN INPUT IN COL A, THERE SHOULD BE INPUT IN EITHER IN COL C OR D OR E OR F**   1. What proportion of your patients with [insert comorbidity from S4] experience each of the following adverse events in relation to treatment for their condition?   **Please enter a percentage for each within the stated range**  **MINIMUM SUM OF TOTAL COLUMN A =1**   1. Of those [insert comorbidity from S4] patients experiencing each of the following adverse events in relation to treatment for their condition, how many times will a typical patient experience each adverse event in a year?   **Please enter a number for each within the stated range**  **MINIMUM SUM OF TOTAL COLUMN Ca, Cb, Cc = 1 IF COLUMN C is coded 1**   1. Of those patients experiencing each of the following adverse events in relation to treatment for their condition, what proportion require off-schedule out-patient visits (excluding ER visits)?   **Please enter a percentage within the stated range**   - 1. How many nurse visits will a typical patient have in a year?   **Please enter a number for each within the stated range [N/A FOR GR]**   - 1. How many GP visits will a typical patient have in a year?   **Please enter a number for each within the stated range**   - 1. How many Specialists visits will a typical patient have in a year?   **Please enter a number for each within the stated range**  **MINIMUM SUM OF TOTAL COLUMN Da, Db = 1 IF COLUMN D is coded 1**   1. Of those patients experiencing each of the following adverse events in relation to treatment for their condition, what proportion require in-patient visits?   **Please enter a percentage within the stated range**   - 1. How many in-patient visits will a typical patient have in a year?   **Please enter a number within the stated range**   - 1. What is the average length of hospital stay (days)?   **Please enter number of days within the stated range**  **MINIMUM SUM OF TOTAL COLUMN Ea, Eb = 1 IF COLUMN E is coded 1**   1. Of those patients experiencing each of the adverse events in relation to treatment for their condition, what proportion require Intensive Care Unit (ICU) visits?   **Please enter a percentage within the stated range**   - 1. How many Intensive Care Unit (ICU) visits will a typical patient have in a year?   **Please enter a number within the stated range**   - 1. What is the average length of ICU stay (days)?   **Please enter number of days within the stated range**  **MINIMUM SUM OF TOTAL COLUMN Fa = 1 IF COLUMN F is coded 1**   1. Of those patients experiencing each of the adverse events in relation to treatment for their condition, what proportion require emergency room visits?   **Please enter a percentage within the stated range**   - 1. How many emergency room visits will a typical patient have in a year?   **Please enter a number within the stated range**   \|  \|  \|  \| \| **Out-patient visits** \| \| \| \| **In-patient visits** \| \| \| **ICU visits** \| \| \| **ER visits** \| \| \| --- \| --- \| --- \| --- \| --- \| --- \| --- \| --- \| --- \| --- \| --- \| --- \| --- \| --- \| --- \| --- \| \|  \| **Adverse events** \| **A**  **% patients**  **(Range in brackets)** \| **B**  **Number in 1 year**  **(Range in brackets)** \| **C**  **% patients**  **(Range in brackets)** \| **C.a**  **Number of Nurse visits**  **(Range in brackets)** \| **C.b**  **Number of GP visits**  **(Range in brackets)** \| **C.c**  **Number of Specialist visits**  **(Range in brackets)** \| **D**  **% patients**  **(Range in brackets)** \| **D.a**  **Number in 1 year**  **(Range in brackets)** \| **D.b**  **Average length of stay (days)**  **(Range in brackets)** \| **E**  **% patients**  **(Range in brackets)** \| **E.a**  **Number in 1 year**  **(Range in brackets)** \| **E.b**  **Average length of stay (days)**  **(Range in brackets)** \| **F**  **% patients**  **(Range in brackets)** \| **F.a**  **Number in 1 year**  **(Range in brackets)** \| \|  \| **Type 2 Diabetes** \|  \|  \|  \|  \|  \|  \|  \|  \|  \|  \|  \|  \|  \|  \| \| **1** \| Hypoglycemia \| (Range: 0 – 100) \| (Range: 1 – 12) \| (Range: 0 – 100) \| (Range: 1 – 12) \| (Range: 1 – 12) \| (Range: 1 – 12) \| (Range: 0 – 100) \| (Range: 1 – 12) \| (Range: 0 – 14) \| (Range: 0 – 100) \| (Range: 1 – 5) \| (Range: 0 – 28) \| (Range: 0 – 100) \| (Range: 1 – 12) \| \| **2** \| Lactic acidosis or ketoacidosis \| (Range: 0 – 100) \| (Range: 1 – 12) \| (Range: 0 – 100) \| (Range: 1 – 12) \| (Range: 1 – 12) \| (Range: 1 – 12) \| (Range: 0 – 100) \| (Range: 1 – 12) \| (Range: 0 – 14) \| (Range: 0 – 100) \| (Range: 1 – 5) \| (Range: 0 – 28) \| (Range: 0 – 100) \| (Range: 1 – 12) \| \| **3** \| Neuropathy \| (Range: 0 – 100) \| (Range: 1 – 12) \| (Range: 0 – 100) \| (Range: 1 – 12) \| (Range: 1 – 12) \| (Range: 1 – 12) \| (Range: 0 – 100) \| (Range: 1 – 12) \| (Range: 0 – 14) \| (Range: 0 – 100) \| (Range: 1 – 5) \| (Range: 0 – 28) \| (Range: 0 – 100) \| (Range: 1 – 12) \| \| **4** \| Congestive heart failure \| (Range: 0 – 100) \| (Range: 1 – 12) \| (Range: 0 – 100) \| (Range: 1 – 12) \| (Range: 1 – 12) \| (Range: 1 – 12) \| (Range: 0 – 100) \| (Range: 1 – 12) \| (Range: 0 – 14) \| (Range: 0 – 100) \| (Range: 1 – 5) \| (Range: 0 – 28) \| (Range: 0 – 100) \| (Range: 1 – 12) \| \| **5** \| Pancreatitis \| (Range: 0 – 100) \| (Range: 1 – 12) \| (Range: 0 – 100) \| (Range: 1 – 12) \| (Range: 1 – 12) \| (Range: 1 – 12) \| (Range: 0 – 100) \| (Range: 1 – 12) \| (Range: 0 – 14) \| (Range: 0 – 100) \| (Range: 1 – 5) \| (Range: 0 – 28) \| (Range: 0 – 100) \| (Range: 1 – 12) \| \| **6** \| Upper respiratory tract infection \| (Range: 0 – 100) \| (Range: 1 – 12) \| (Range: 0 – 100) \| (Range: 1 – 12) \| (Range: 1 – 12) \| (Range: 1 – 12) \| (Range: 0 – 100) \| (Range: 1 – 12) \| (Range: 0 – 14) \| (Range: 0 – 100) \| (Range: 1 – 5) \| (Range: 0 – 28) \| (Range: 0 – 100) \| (Range: 1 – 12) \| \| **7** \| Genital mycosis \| (Range: 0 – 100) \| (Range: 1 – 12) \| (Range: 0 – 100) \| (Range: 1 – 12) \| (Range: 1 – 12) \| (Range: 1 – 12) \| (Range: 0 – 100) \| (Range: 1 – 12) \| (Range: 0 – 14) \| (Range: 0 – 100) \| (Range: 1 – 5) \| (Range: 0 – 28) \| (Range: 0 – 100) \| (Range: 1 – 12) \| \| **8** \| Dyslipidemia \| (Range: 0 – 100) \| (Range: 1 – 12) \| (Range: 0 – 100) \| (Range: 1 – 12) \| (Range: 1 – 12) \| (Range: 1 – 12) \| (Range: 0 – 100) \| (Range: 1 – 12) \| (Range: 0 – 14) \| (Range: 0 – 100) \| (Range: 1 – 5) \| (Range: 0 – 28) \| (Range: 0 – 100) \| (Range: 1 – 12) \| \| **9** \| Osteoporosis \| (Range: 0 – 100) \| (Range: 1 – 12) \| (Range: 0 – 100) \| (Range: 1 – 12) \| (Range: 1 – 12) \| (Range: 1 – 12) \| (Range: 0 – 100) \| (Range: 1 – 12) \| (Range: 0 – 14) \| (Range: 0 – 100) \| (Range: 1 – 5) \| (Range: 0 – 28) \| (Range: 0 – 100) \| (Range: 1 – 12) \| \| **10** \| Injection site reactions \| (Range: 0 – 100) \| (Range: 1 – 12) \| (Range: 0 – 100) \| (Range: 1 – 12) \| (Range: 1 – 12) \| (Range: 1 – 12) \| (Range: 0 – 100) \| (Range: 1 – 12) \| (Range: 0 – 14) \| (Range: 0 – 100) \| (Range: 1 – 5) \| (Range: 0 – 28) \| (Range: 0 – 100) \| (Range: 1 – 12) \| \| **11** \| Weight gain \| (Range: 0 – 100) \| (Range: 1 – 12) \| (Range: 0 – 100) \| (Range: 1 – 12) \| (Range: 1 – 12) \| (Range: 1 – 12) \| (Range: 0 – 100) \| (Range: 1 – 12) \| (Range: 0 – 14) \| (Range: 0 – 100) \| (Range: 1 – 5) \| (Range: 0 – 28) \| (Range: 0 – 100) \| (Range: 1 – 12) \| \| **12** \| Allergic reaction to treatment \| (Range: 0 – 100) \| (Range: 1 – 12) \| (Range: 0 – 100) \| (Range: 1 – 12) \| (Range: 0 – 12) \| (Range: 1 – 12) \| (Range: 0 – 100) \| (Range: 1 – 12) \| (Range: 0 – 14) \| (Range: 0 – 100) \| (Range: 1 – 5) \| (Range: 0 – 28) \| (Range: 0 – 100) \| (Range: 1 – 12) \| \| **13** \| Myocardial infarction **[FOR ROM ONLY]** \| (Range: 0 – 100) \| (Range: 1 – 12) \| (Range: 0 – 100) \| (Range: 1 – 12) \| (Range: 0 – 12) \| (Range: 1 – 12) \| (Range: 0 – 100) \| (Range: 1 – 12) \| (Range: 0 – 14) \| (Range: 0 – 100) \| (Range: 1 – 5) \| (Range: 0 – 28) \| (Range: 0 – 100) \| (Range: 1 – 12) \| \| **13.1** \| Worsening of renal function  **[FOR GR ONLY]** \| (Range: 0 – 100) \| (Range: 1 – 12) \| (Range: 0 – 100) \| (Range: 1 – 12) \| (Range: 0 – 12) \| (Range: 1 – 12) \| (Range: 0 – 100) \| (Range: 1 – 12) \| (Range: 0 – 14) \| (Range: 0 – 100) \| (Range: 1 – 5) \| (Range: 0 – 28) \| (Range: 0 – 100) \| (Range: 1 – 12) \| \|  \| **Asthma** \|  \|  \|  \|  \|  \|  \|  \|  \|  \|  \|  \|  \|  \|  \| \| **14** \| Cardiac stimulation e.g. arrhythmia \| (Range: 0 – 100) \| (Range: 1 – 12) \| (Range: 0 – 100) \| (Range: 1 – 12) \| (Range: 0 – 12) \| (Range: 1 – 12) \| (Range: 0 – 100) \| (Range: 1 – 12) \| (Range: 0 – 14) \| (Range: 0 – 100) \| (Range: 1 – 5) \| (Range: 0 – 28) \| (Range: 0 – 100) \| (Range: 1 – 12) \| \| **15** \| CNS stimulation – e.g. tremors, nervousness, insomnia, seizures, headaches \| (Range: 0 – 100) \| (Range: 1 – 12) \| (Range: 0 – 100) \| (Range: 1 – 12) \| (Range: 0 – 12) \| (Range: 1 – 12) \| (Range: 0 – 100) \| (Range: 1 – 12) \| (Range: 0 – 14) \| (Range: 0 – 100) \| (Range: 1 – 5) \| (Range: 0 – 28) \| (Range: 0 – 100) \| (Range: 1 – 12) \| \| **16** \| Respiratory irritation \| (Range: 0 – 100) \| (Range: 1 – 12) \| (Range: 0 – 100) \| (Range: 1 – 12) \| (Range: 0 – 12) \| (Range: 1 – 12) \| (Range: 0 – 100) \| (Range: 1 – 12) \| (Range: 0 – 14) \| (Range: 0 – 100) \| (Range: 1 – 5) \| (Range: 0 – 28) \| (Range: 0 – 100) \| (Range: 1 – 12) \| \| **17** \| Secondary infections e.g. Nasopharyngitis, fever, oropharyngeal candidiasis \| (Range: 0 – 100) \| (Range: 1 – 12) \| (Range: 0 – 100) \| (Range: 1 – 12) \| (Range: 0 – 12) \| (Range: 1 – 12) \| (Range: 0 – 100) \| (Range: 1 – 12) \| (Range: 0 – 14) \| (Range: 0 – 100) \| (Range: 1 – 5) \| (Range: 0 – 28) \| (Range: 0 – 100) \| (Range: 1 – 12) \| \| **18** \| Adrenal insufficiency \| (Range: 0 – 100) \| (Range: 1 – 12) \| (Range: 0 – 100) \| (Range: 1 – 12) \| (Range: 0 – 12) \| (Range: 1 – 12) \| (Range: 0 – 100) \| (Range: 1 – 12) \| (Range: 0 – 14) \| (Range: 0 – 100) \| (Range: 1 – 5) \| (Range: 0 – 28) \| (Range: 0 – 100) \| (Range: 1 – 12) \| \| **19** \| Ocular events \| (Range: 0 – 100) \| (Range: 1 – 12) \| (Range: 0 – 100) \| (Range: 1 – 12) \| (Range: 0 – 12) \| (Range: 1 – 12) \| (Range: 0 – 100) \| (Range: 1 – 12) \| (Range: 0 – 14) \| (Range: 0 – 100) \| (Range: 1 – 5) \| (Range: 0 – 28) \| (Range: 0 – 100) \| (Range: 1 – 12) \| \| **20** \| Psychiatric events \| (Range: 0 – 100) \| (Range: 1 – 12) \| (Range: 0 – 100) \| (Range: 1 – 12) \| (Range: 0 – 12) \| (Range: 1 – 12) \| (Range: 0 – 100) \| (Range: 1 – 12) \| (Range: 0 – 14) \| (Range: 0 – 100) \| (Range: 1 – 5) \| (Range: 0 – 28) \| (Range: 0 – 100) \| (Range: 1 – 12) \| \|  \| **Sleep apnea** \|  \|  \|  \|  \|  \|  \|  \|  \|  \|  \|  \|  \|  \|  \| \| **21** \| Nasal irritation and congestion \| (Range: 0 – 100) \| (Range: 1 – 12) \| (Range: 0 – 100) \| (Range: 1 – 12) \| (Range: 0 – 12) \| (Range: 1 – 12) \| (Range: 0 – 100) \| (Range: 1 – 12) \| (Range: 0 – 14) \| (Range: 0 – 100) \| (Range: 1 – 5) \| (Range: 0 – 28) \| (Range: 0 – 100) \| (Range: 1 – 12) \| \| **22** \| Skin irritation or sores \| (Range: 0 – 100) \| (Range: 1 – 12) \| (Range: 0 – 100) \| (Range: 1 – 12) \| (Range: 0 – 12) \| (Range: 1 – 12) \| (Range: 0 – 100) \| (Range: 1 – 12) \| (Range: 0 – 14) \| (Range: 0 – 100) \| (Range: 1 – 5) \| (Range: 0 – 28) \| (Range: 0 – 100) \| (Range: 1 – 12) \| \|  \| **Osteoarthritis** \|  \|  \|  \|  \|  \|  \|  \|  \|  \|  \|  \|  \|  \|  \| \| **23** \| Gastrointestinal bleeding \| (Range: 0 – 100) \| (Range: 1 – 12) \| (Range: 0 – 100) \| (Range: 1 – 12) \| (Range: 0 – 12) \| (Range: 1 – 12) \| (Range: 0 – 100) \| (Range: 1 – 12) \| (Range: 0 – 14) \| (Range: 0 – 100) \| (Range: 1 – 5) \| (Range: 0 – 28) \| (Range: 0 – 100) \| (Range: 1 – 12) \| \| **24** \| Hepatotoxicity \| (Range: 0 – 100) \| (Range: 1 – 12) \| (Range: 0 – 100) \| (Range: 1 – 12) \| (Range: 0 – 12) \| (Range: 1 – 12) \| (Range: 0 – 100) \| (Range: 1 – 12) \| (Range: 0 – 14) \| (Range: 0 – 100) \| (Range: 1 – 5) \| (Range: 0 – 28) \| (Range: 0 – 100) \| (Range: 1 – 12) \| \| **25** \| Nephrotoxicity \| (Range: 0 – 100) \| (Range: 1 – 12) \| (Range: 0 – 100) \| (Range: 1 – 12) \| (Range: 0 – 12) \| (Range: 1 – 12) \| (Range: 0 – 100) \| (Range: 1 – 12) \| (Range: 0 – 14) \| (Range: 0 – 100) \| (Range: 1 – 5) \| (Range: 0 – 28) \| (Range: 0 – 100) \| (Range: 1 – 12) \| \| **26** \| Hematologic changes \| (Range: 0 – 100) \| (Range: 1 – 12) \| (Range: 0 – 100) \| (Range: 1 – 12) \| (Range: 0 – 12) \| (Range: 1 – 12) \| (Range: 0 – 100) \| (Range: 1 – 12) \| (Range: 0 – 14) \| (Range: 0 – 100) \| (Range: 1 – 5) \| (Range: 0 – 28) \| (Range: 0 – 100) \| (Range: 1 – 12) \| \| **27** \| Nausea and vomiting \| (Range: 0 – 100) \| (Range: 1 – 12) \| (Range: 0 – 100) \| (Range: 1 – 12) \| (Range: 0 – 12) \| (Range: 1 – 12) \| (Range: 0 – 100) \| (Range: 1 – 12) \| (Range: 0 – 14) \| (Range: 0 – 100) \| (Range: 1 – 5) \| (Range: 0 – 28) \| (Range: 0 – 100) \| (Range: 1 – 12) \| \| **28** \| Hypersensitivity reactions \| (Range: 0 – 100) \| (Range: 1 – 12) \| (Range: 0 – 100) \| (Range: 1 – 12) \| (Range: 0 – 12) \| (Range: 1 – 12) \| (Range: 0 – 100) \| (Range: 1 – 12) \| (Range: 0 – 14) \| (Range: 0 – 100) \| (Range: 1 – 5) \| (Range: 0 – 28) \| (Range: 0 – 100) \| (Range: 1 – 12) \| \|  \| **Heart failure** \|  \|  \|  \|  \|  \|  \|  \|  \|  \|  \|  \|  \|  \|  \| \| **29** \| Hyperkalemia/serum electrolyte imbalance \| (Range: 0 – 100) \| (Range: 1 – 12) \| (Range: 0 – 100) \| (Range: 1 – 12) \| (Range: 0 – 12) \| (Range: 1 – 12) \| (Range: 0 – 100) \| (Range: 1 – 12) \| (Range: 0 – 14) \| (Range: 0 – 100) \| (Range: 1 – 5) \| (Range: 0 – 28) \| (Range: 0 – 100) \| (Range: 1 – 12) \| \| **30** \| Metabolic acidosis/alkalosis \| (Range: 0 – 100) \| (Range: 1 – 12) \| (Range: 0 – 100) \| (Range: 1 – 12) \| (Range: 0 – 12) \| (Range: 1 – 12) \| (Range: 0 – 100) \| (Range: 1 – 12) \| (Range: 0 – 14) \| (Range: 0 – 100) \| (Range: 1 – 5) \| (Range: 0 – 28) \| (Range: 0 – 100) \| (Range: 1 – 12) \| \| **31** \| Worsening of renal function \| (Range: 0 – 100) \| (Range: 1 – 12) \| (Range: 0 – 100) \| (Range: 1 – 12) \| (Range: 0 – 12) \| (Range: 1 – 12) \| (Range: 0 – 100) \| (Range: 1 – 12) \| (Range: 0 – 14) \| (Range: 0 – 100) \| (Range: 1 – 5) \| (Range: 0 – 28) \| (Range: 0 – 100) \| (Range: 1 – 12) \| \| **32** \| Hypotension \| (Range: 0 – 100) \| (Range: 1 – 12) \| (Range: 0 – 100) \| (Range: 1 – 12) \| (Range: 0 – 12) \| (Range: 1 – 12) \| (Range: 0 – 100) \| (Range: 1 – 12) \| (Range: 0 – 14) \| (Range: 0 – 100) \| (Range: 1 – 5) \| (Range: 0 – 28) \| (Range: 0 – 100) \| (Range: 1 – 12) \| \| **33** \| Arrhythmia \| (Range: 0 – 100) \| (Range: 1 – 12) \| (Range: 0 – 100) \| (Range: 1 – 12) \| (Range: 0 – 12) \| (Range: 1 – 12) \| (Range: 0 – 100) \| (Range: 1 – 12) \| (Range: 0 – 14) \| (Range: 0 – 100) \| (Range: 1 – 5) \| (Range: 0 – 28) \| (Range: 0 – 100) \| (Range: 1 – 12) \| \| **34** \| Bronchospasm \| (Range: 0 – 100) \| (Range: 1 – 12) \| (Range: 0 – 100) \| (Range: 1 – 12) \| (Range: 0 – 12) \| (Range: 1 – 12) \| (Range: 0 – 100) \| (Range: 1 – 12) \| (Range: 0 – 14) \| (Range: 0 – 100) \| (Range: 1 – 5) \| (Range: 0 – 28) \| (Range: 0 – 100) \| (Range: 1 – 12) \| \|  \| **Hypertension** \|  \|  \|  \|  \|  \|  \|  \|  \|  \|  \|  \|  \|  \|  \| \| **35** \| Serum electrolyte imbalance (hyper- or hypokalemia) \| (Range: 0 – 100) \| (Range: 1 – 12) \| (Range: 0 – 100) \| (Range: 1 – 12) \| (Range: 0 – 12) \| (Range: 1 – 12) \| (Range: 0 – 100) \| (Range: 1 – 12) \| (Range: 0 – 14) \| (Range: 0 – 100) \| (Range: 1 – 5) \| (Range: 0 – 28) \| (Range: 0 – 100) \| (Range: 1 – 12) \| \| **36** \| Arrhythmia \| (Range: 0 – 100) \| (Range: 1 – 12) \| (Range: 0 – 100) \| (Range: 1 – 12) \| (Range: 0 – 12) \| (Range: 1 – 12) \| (Range: 0 – 100) \| (Range: 1 – 12) \| (Range: 0 – 14) \| (Range: 0 – 100) \| (Range: 1 – 5) \| (Range: 0 – 28) \| (Range: 0 – 100) \| (Range: 1 – 12) \| \| **37** \| Acute kidney injury \| (Range: 0 – 100) \| (Range: 1 – 12) \| (Range: 0 – 100) \| (Range: 1 – 12) \| (Range: 0 – 12) \| (Range: 1 – 12) \| (Range: 0 – 100) \| (Range: 1 – 12) \| (Range: 0 – 14) \| (Range: 0 – 100) \| (Range: 1 – 5) \| (Range: 0 – 28) \| (Range: 0 – 100) \| (Range: 1 – 12) \| \| **38** \| Visual changes \| (Range: 0 – 100) \| (Range: 1 – 12) \| (Range: 0 – 100) \| (Range: 1 – 12) \| (Range: 0 – 12) \| (Range: 1 – 12) \| (Range: 0 – 100) \| (Range: 1 – 12) \| (Range: 0 – 14) \| (Range: 0 – 100) \| (Range: 1 – 5) \| (Range: 0 – 28) \| (Range: 0 – 100) \| (Range: 1 – 12) \| \| **39** \| Metabolic acidosis/alkalosis \| (Range: 0 – 100) \| (Range: 1 – 12) \| (Range: 0 – 100) \| (Range: 1 – 12) \| (Range: 0 – 12) \| (Range: 1 – 12) \| (Range: 0 – 100) \| (Range: 1 – 12) \| (Range: 0 – 14) \| (Range: 0 – 100) \| (Range: 1 – 5) \| (Range: 0 – 28) \| (Range: 0 – 100) \| (Range: 1 – 12) \| \| **40** \| Hypotension \| (Range: 0 – 100) \| (Range: 1 – 12) \| (Range: 0 – 100) \| (Range: 1 – 12) \| (Range: 0 – 12) \| (Range: 1 – 12) \| (Range: 0 – 100) \| (Range: 1 – 12) \| (Range: 0 – 14) \| (Range: 0 – 100) \| (Range: 1 – 5) \| (Range: 0 – 28) \| (Range: 0 – 100) \| (Range: 1 – 12) \| \|  \| **Dyslipidemia** \|  \|  \|  \|  \|  \|  \|  \|  \|  \|  \|  \|  \|  \|  \| \| **41** \| Hepatotoxicity, hepatic dysfunction \| (Range: 0 – 100) \| (Range: 1 – 12) \| (Range: 0 – 100) \| (Range: 1 – 12) \| (Range: 0 – 12) \| (Range: 1 – 12) \| (Range: 0 – 100) \| (Range: 1 – 12) \| (Range: 0 – 14) \| (Range: 0 – 100) \| (Range: 1 – 5) \| (Range: 0 – 28) \| (Range: 0 – 100) \| (Range: 1 – 12) \| \| **42** \| Myopathy \| (Range: 0 – 100) \| (Range: 1 – 12) \| (Range: 0 – 100) \| (Range: 1 – 12) \| (Range: 0 – 12) \| (Range: 1 – 12) \| (Range: 0 – 100) \| (Range: 1 – 12) \| (Range: 0 – 14) \| (Range: 0 – 100) \| (Range: 1 – 5) \| (Range: 0 – 28) \| (Range: 0 – 100) \| (Range: 1 – 12) \| \| **43** \| Rhabdomyolysis \| (Range: 0 – 100) \| (Range: 1 – 12) \| (Range: 0 – 100) \| (Range: 1 – 12) \| (Range: 0 – 12) \| (Range: 1 – 12) \| (Range: 0 – 100) \| (Range: 1 – 12) \| (Range: 0 – 14) \| (Range: 0 – 100) \| (Range: 1 – 5) \| (Range: 0 – 28) \| (Range: 0 – 100) \| (Range: 1 – 12) \| \| **44** \| GI distress \| (Range: 0 – 100) \| (Range: 1 – 12) \| (Range: 0 – 100) \| (Range: 1 – 12) \| (Range: 0 – 12) \| (Range: 1 – 12) \| (Range: 0 – 100) \| (Range: 1 – 12) \| (Range: 0 – 14) \| (Range: 0 – 100) \| (Range: 1 – 5) \| (Range: 0 – 28) \| (Range: 0 – 100) \| (Range: 1 – 12) \| \| **45** \| Cholesterol gallstones \| (Range: 0 – 100) \| (Range: 1 – 12) \| (Range: 0 – 100) \| (Range: 1 – 12) \| (Range: 0 – 12) \| (Range: 1 – 12) \| (Range: 0 – 100) \| (Range: 1 – 12) \| (Range: 0 – 14) \| (Range: 0 – 100) \| (Range: 1 – 5) \| (Range: 0 – 28) \| (Range: 0 – 100) \| (Range: 1 – 12) \| \|  \| **Atrial Fibrillation** \|  \|  \|  \|  \|  \|  \|  \|  \|  \|  \|  \|  \|  \|  \| \| **46** \| Intracranial hemorrhage \| (Range: 0 – 100) \| (Range: 1 – 12) \| (Range: 0 – 100) \| (Range: 1 – 12) \| (Range: 0 – 12) \| (Range: 1 – 12) \| (Range: 0 – 100) \| (Range: 1 – 12) \| (Range: 0 – 14) \| (Range: 0 – 100) \| (Range: 1 – 5) \| (Range: 0 – 28) \| (Range: 0 – 100) \| (Range: 1 – 12) \| \| **47** \| Major non-cerebral bleed (e.g. GI bleed) \| (Range: 0 – 100) \| (Range: 1 – 12) \| (Range: 0 – 100) \| (Range: 1 – 12) \| (Range: 0 – 12) \| (Range: 1 – 12) \| (Range: 0 – 100) \| (Range: 1 – 12) \| (Range: 0 – 14) \| (Range: 0 – 100) \| (Range: 1 – 5) \| (Range: 0 – 28) \| (Range: 0 – 100) \| (Range: 1 – 12) \| \| **48** \| Sinus bradycardia \| (Range: 0 – 100) \| (Range: 1 – 12) \| (Range: 0 – 100) \| (Range: 1 – 12) \| (Range: 0 – 12) \| (Range: 1 – 12) \| (Range: 0 – 100) \| (Range: 1 – 12) \| (Range: 0 – 14) \| (Range: 0 – 100) \| (Range: 1 – 5) \| (Range: 0 – 28) \| (Range: 0 – 100) \| (Range: 1 – 12) \| \|  \| **Angina** \|  \|  \|  \|  \|  \|  \|  \|  \|  \|  \|  \|  \|  \|  \| \| **49** \| Angioedema \| (Range: 0 – 100) \| (Range: 1 – 12) \| (Range: 0 – 100) \| (Range: 1 – 12) \| (Range: 0 – 12) \| (Range: 1 – 12) \| (Range: 0 – 100) \| (Range: 1 – 12) \| (Range: 0 – 14) \| (Range: 0 – 100) \| (Range: 1 – 5) \| (Range: 0 – 28) \| (Range: 0 – 100) \| (Range: 1 – 12) \| \| **50** \| Hyperkalemia \| (Range: 0 – 100) \| (Range: 1 – 12) \| (Range: 0 – 100) \| (Range: 1 – 12) \| (Range: 0 – 12) \| (Range: 1 – 12) \| (Range: 0 – 100) \| (Range: 1 – 12) \| (Range: 0 – 14) \| (Range: 0 – 100) \| (Range: 1 – 5) \| (Range: 0 – 28) \| (Range: 0 – 100) \| (Range: 1 – 12) \| \| **51** \| Hypotension \| (Range: 0 – 100) \| (Range: 1 – 12) \| (Range: 0 – 100) \| (Range: 1 – 12) \| (Range: 0 – 12) \| (Range: 1 – 12) \| (Range: 0 – 100) \| (Range: 1 – 12) \| (Range: 0 – 14) \| (Range: 0 – 100) \| (Range: 1 – 5) \| (Range: 0 – 28) \| (Range: 0 – 100) \| (Range: 1 – 12) \| \|  \| **Chronic Kidney Disease** \|  \|  \|  \|  \|  \|  \|  \|  \|  \|  \|  \|  \|  \|  \| \| **52** \| Hyperkalemia \| (Range: 0 – 100) \| (Range: 1 – 12) \| (Range: 0 – 100) \| (Range: 1 – 12) \| (Range: 0 – 12) \| (Range: 1 – 12) \| (Range: 0 – 100) \| (Range: 1 – 12) \| (Range: 0 – 14) \| (Range: 0 – 100) \| (Range: 1 – 5) \| (Range: 0 – 28) \| (Range: 0 – 100) \| (Range: 1 – 12) \| \| **53** \| Hemodynamic instability \| (Range: 0 – 100) \| (Range: 1 – 12) \| (Range: 0 – 100) \| (Range: 1 – 12) \| (Range: 0 – 12) \| (Range: 1 – 12) \| (Range: 0 – 100) \| (Range: 1 – 12) \| (Range: 0 – 14) \| (Range: 0 – 100) \| (Range: 1 – 5) \| (Range: 0 – 28) \| (Range: 0 – 100) \| (Range: 1 – 12) \| \| **54** \| Hypertension or hypotension \| (Range: 0 – 100) \| (Range: 1 – 12) \| (Range: 0 – 100) \| (Range: 1 – 12) \| (Range: 0 – 12) \| (Range: 1 – 12) \| (Range: 0 – 100) \| (Range: 1 – 12) \| (Range: 0 – 14) \| (Range: 0 – 100) \| (Range: 1 – 5) \| (Range: 0 – 28) \| (Range: 0 – 100) \| (Range: 1 – 12) \| \| **55** \| Thrombosis \| (Range: 0 – 100) \| (Range: 1 – 12) \| (Range: 0 – 100) \| (Range: 1 – 12) \| (Range: 0 – 12) \| (Range: 1 – 12) \| (Range: 0 – 100) \| (Range: 1 – 12) \| (Range: 0 – 14) \| (Range: 0 – 100) \| (Range: 1 – 5) \| (Range: 0 – 28) \| (Range: 0 – 100) \| (Range: 1 – 12) \| |
| --- | --- | --- | --- | --- | --- | --- | --- | --- | --- | --- | --- | --- | --- | --- | --- | --- | --- | --- | --- | --- | --- | --- | --- | --- | --- | --- | --- | --- | --- | --- | --- | --- | --- | --- | --- | --- | --- | --- | --- | --- | --- | --- | --- | --- | --- | --- | --- | --- | --- | --- | --- | --- | --- | --- | --- | --- | --- | --- | --- | --- | --- | --- | --- | --- | --- | --- | --- | --- | --- | --- | --- | --- | --- | --- | --- | --- | --- | --- | --- | --- | --- | --- | --- | --- | --- | --- | --- | --- | --- | --- | --- | --- | --- | --- | --- | --- | --- | --- | --- | --- | --- | --- | --- | --- | --- | --- | --- | --- | --- | --- | --- | --- | --- | --- | --- | --- | --- | --- | --- | --- | --- | --- | --- | --- | --- | --- | --- | --- | --- | --- | --- | --- | --- | --- | --- | --- | --- | --- | --- | --- | --- | --- | --- | --- | --- | --- | --- | --- | --- | --- | --- | --- | --- | --- | --- | --- | --- | --- | --- | --- | --- | --- | --- | --- | --- | --- | --- | --- | --- | --- | --- | --- | --- | --- | --- | --- | --- | --- | --- | --- | --- | --- | --- | --- | --- | --- | --- | --- | --- | --- | --- | --- | --- | --- | --- | --- | --- | --- | --- | --- | --- | --- | --- | --- | --- | --- | --- | --- | --- | --- | --- | --- | --- | --- | --- | --- | --- | --- | --- | --- | --- | --- | --- | --- | --- | --- | --- | --- | --- | --- | --- | --- | --- | --- | --- | --- | --- | --- | --- | --- | --- | --- | --- | --- | --- | --- | --- | --- | --- | --- | --- | --- | --- | --- | --- | --- | --- | --- | --- | --- | --- | --- | --- | --- | --- | --- | --- | --- | --- | --- | --- | --- | --- | --- | --- | --- | --- | --- | --- | --- | --- | --- | --- | --- | --- | --- | --- | --- | --- | --- | --- | --- | --- | --- | --- | --- | --- | --- | --- | --- | --- | --- | --- | --- | --- | --- | --- | --- | --- | --- | --- | --- | --- | --- | --- | --- | --- | --- | --- | --- | --- | --- | --- | --- | --- | --- | --- | --- | --- | --- | --- | --- | --- | --- | --- | --- | --- | --- | --- | --- | --- | --- | --- | --- | --- | --- | --- | --- | --- | --- | --- | --- | --- | --- | --- | --- | --- | --- | --- | --- | --- | --- | --- | --- | --- | --- | --- | --- | --- | --- | --- | --- | --- | --- | --- | --- | --- | --- | --- | --- | --- | --- | --- | --- | --- | --- | --- | --- | --- | --- | --- | --- | --- | --- | --- | --- | --- | --- | --- | --- | --- | --- | --- | --- | --- | --- | --- | --- | --- | --- | --- | --- | --- | --- | --- | --- | --- | --- | --- | --- | --- | --- | --- | --- | --- | --- | --- | --- | --- | --- | --- | --- | --- | --- | --- | --- | --- | --- | --- | --- | --- | --- | --- | --- | --- | --- | --- | --- | --- | --- | --- | --- | --- | --- | --- | --- | --- | --- | --- | --- | --- | --- | --- | --- | --- | --- | --- | --- | --- | --- | --- | --- | --- | --- | --- | --- | --- | --- | --- | --- | --- | --- | --- | --- | --- | --- | --- | --- | --- | --- | --- | --- | --- | --- | --- | --- | --- | --- | --- | --- | --- | --- | --- | --- | --- | --- | --- | --- | --- | --- | --- | --- | --- | --- | --- | --- | --- | --- | --- | --- | --- | --- | --- | --- | --- | --- | --- | --- | --- | --- | --- | --- | --- | --- | --- | --- | --- | --- | --- | --- | --- | --- | --- | --- | --- | --- | --- | --- | --- | --- | --- | --- | --- | --- | --- | --- | --- | --- | --- | --- | --- | --- | --- | --- | --- | --- | --- | --- | --- | --- | --- | --- | --- | --- | --- | --- | --- | --- | --- | --- | --- | --- | --- | --- | --- | --- | --- | --- | --- | --- | --- | --- | --- | --- | --- | --- | --- | --- | --- | --- | --- | --- | --- | --- | --- | --- | --- | --- | --- | --- | --- | --- | --- | --- | --- | --- | --- | --- | --- | --- | --- | --- | --- | --- | --- | --- | --- | --- | --- | --- | --- | --- | --- | --- | --- | --- | --- | --- | --- | --- | --- | --- | --- | --- | --- | --- | --- | --- | --- | --- | --- | --- | --- | --- | --- | --- | --- | --- | --- | --- | --- | --- | --- | --- | --- | --- | --- | --- | --- | --- | --- | --- | --- | --- | --- | --- | --- | --- | --- | --- | --- | --- | --- | --- | --- | --- | --- | --- | --- | --- | --- | --- | --- | --- | --- | --- | --- | --- | --- | --- | --- | --- | --- | --- | --- | --- | --- | --- | --- | --- | --- | --- | --- | --- | --- | --- | --- | --- | --- | --- | --- | --- | --- | --- | --- | --- | --- | --- | --- | --- | --- | --- | --- | --- | --- | --- | --- | --- | --- | --- | --- | --- | --- | --- | --- | --- | --- | --- | --- | --- | --- | --- | --- | --- | --- | --- | --- | --- | --- | --- | --- | --- | --- | --- | --- | --- | --- | --- | --- | --- | --- | --- | --- | --- | --- | --- | --- | --- | --- | --- | --- | --- | --- | --- | --- | --- | --- | --- | --- | --- | --- | --- | --- | --- | --- | --- | --- | --- | --- | --- | --- | --- | --- | --- | --- | --- | --- | --- | --- | --- | --- | --- | --- | --- | --- | --- | --- | --- | --- | --- | --- | --- | --- | --- | --- | --- | --- | --- | --- | --- | --- | --- | --- | --- | --- | --- | --- | --- | --- | --- | --- | --- | --- | --- | --- | --- | --- | --- | --- | --- | --- | --- | --- | --- | --- | --- | --- | --- | --- | --- | --- | --- | --- | --- | --- | --- | --- | --- | --- | --- | --- | --- | --- | --- | --- | --- | --- | --- | --- | --- | --- | --- | --- | --- | --- | --- | --- | --- | --- | --- | --- | --- | --- | --- | --- | --- | --- | --- | --- | --- | --- | --- | --- | --- | --- | --- | --- | --- | --- | --- | --- | --- | --- | --- | --- | --- | --- | --- | --- | --- | --- | --- | --- | --- | --- | --- | --- | --- | --- | --- | --- | --- | --- | --- | --- | --- | --- | --- | --- | --- | --- | --- | --- | --- | --- | --- | --- | --- | --- | --- | --- | --- | --- | --- | --- | --- | --- | --- | --- | --- | --- | --- | --- | --- | --- | --- | --- | --- | --- | --- | --- | --- | --- | --- | --- | --- | --- | --- | --- | --- | --- | --- | --- | --- | --- | --- | --- | --- | --- | --- | --- | --- | --- | --- | --- | --- | --- | --- | --- | --- | --- | --- | --- | --- | --- | --- | --- | --- | --- | --- | --- | --- | --- | --- | --- | --- | --- | --- | --- | --- | --- | --- | --- | --- | --- | --- | --- | --- | --- | --- | --- | --- | --- | --- | --- | --- | --- | --- | --- | --- | --- | --- | --- | --- | --- | --- | --- | --- | --- | --- | --- | --- | --- | --- | --- | --- | --- | --- | --- | --- | --- | --- | --- | --- | --- | --- | --- | --- | --- | --- | --- | --- | --- | --- | --- | --- | --- | --- | --- | --- | --- | --- | --- | --- | --- | --- | --- | --- | --- |

| **COMPLICATIONS HCRU** |
| --- |

| Q8 | **PIPE IN SELECTION FROM S1**  **SHOW GRID TABLE WITH PRE-FILLED RANGES, ALLOW RESPONDENTS TO OVERWRITE ON CELLS**  **SHOW PROMPT IF INPUT IS OUT OF RANGE SPECIFIED**  **DO NOT ALLOW part B - F to be entered if part A was CODED 0 for “% patients”**  **IF Q8a > 0, THEN MINIMUM SUM OF Q8C, Q8D, Q8E AND Q8F = 1**  **MINIMUM SUM OF TOTAL COLUMN A =1**   1. What proportion of your patients with [insert comorbidity from S4] experience each of the following complications in relation to their condition (not treatment)?   **Please enter a percentage for each within the stated range**   1. Of those [insert comorbidity from S4] patients experiencing each of the following complications in relation to their condition (not treatment), how many times will a typical patient experience each complication in relation to their condition in a year?   **Please enter a number for each within the stated range**  **MINIMUM SUM OF TOTAL COLUMN Ca, Cb, Cc = 1 IF COLUMN C is coded 1**   1. Of those patients experiencing each of the following complications in relation to their condition (not treatment), what proportion require off-schedule out-patient visits (excluding ER visits)?   **Please enter a percentage within the stated range**     1. How many nurse visits will a typical patient have in a year?   **Please enter a number for each within the stated range [N/A FOR GR]**   1. How many GP visits will a typical patient have in a year?   **Please enter a number for each within the stated range**   1. How many Specialists visits will a typical patient have in a year?   **Please enter a number for each within the stated range**  **MINIMUM SUM OF TOTAL COLUMN Da, Db, Dc = 1 IF COLUMN D is coded 1**   1. Of those patients experiencing each of the following complications in relation to their condition (not treatment), what proportion require in-patient visits?   **Please enter a percentage within the stated range**   1. How many in-patient visits will a typical patient have in a year?   **Please enter a number within the stated range**   1. What is the average length of hospital stay (days)?   **Please enter number of days within the stated range**  **MINIMUM SUM OF TOTAL COLUMN Ea, Eb = 1 IF COLUMN E is coded 1**   1. Of those patients experiencing each of the following complications in relation to their condition (not treatment), what proportion require Intensive Care Unit (ICU) visits?   **Please enter a percentage within the stated range**   1. How many Intensive Care Unit (ICU) visits will a typical patient have in a year?   **Please enter a number within the stated range**   1. What is the average length of ICU stay (days)?   **Please enter number of days within the stated range**  **MINIMUM SUM OF TOTAL COLUMN Fa = 1 IF COLUMN F is coded 1**   1. Of those patients experiencing each of the following complications in relation to their condition (not treatment), what proportion require emergency room visits?   **Please enter a percentage within the stated range**   1. How many emergency room visits will a typical patient have in a year?   **Please enter a number within the stated range**   \|  \|  \|  \| \| **Out-patient visits** \| \| \| \| **In-patient visits** \| \| \| **ICU visits** \| \| \| **ER visits** \| \| \| --- \| --- \| --- \| --- \| --- \| --- \| --- \| --- \| --- \| --- \| --- \| --- \| --- \| --- \| --- \| --- \| \|  \| **Complications** \| **A**  **% patients**  **(Range in brackets)** \| **B**  **Number in 1 year**  **(Range in brackets)** \| **C**  **% patients**  **(Range in brackets)** \| **C.a**  **Number of Nurse visits**  **(Range in brackets)** \| **C.B**  **Number of GP visits**  **(Range in brackets)** \| **C.C**  **Number of Specialist visits**  **(Range in brackets)** \| **D**  **% patients**  **(Range in brackets)** \| **D.a**  **Number in 1 year**  **(Range in brackets)** \| **D.b**  **Average length of stay (days)**  **(Range in brackets)** \| **E**  **% patients**  **(Range in brackets)** \| **E.a**  **Number in 1 year**  **(Range in brackets)** \| **E.b**  **Average length of stay (days)**  **(Range in brackets)** \| **F**  **% patients**  **(Range in brackets)** \| **F.a**  **Number in 1 year**  **(Range in brackets)** \| \|  \| **T2D** \|  \|  \|  \|  \|  \|  \|  \|  \|  \|  \|  \|  \|  \|  \| \| **1** \| Neuropathy \| (Range: 0 – 100) \| (Range: 1 – 12) \| (Range: 0 – 100) \| (Range: 1 – 12) \| (Range: 1 – 12) \| (Range: 1 – 12) \| (Range: 0 – 100) \| (Range: 1 – 12) \| (Range: 1 – 14) \| (Range: 0 – 100) \| (Range: 1 – 5) \| (Range: 1 – 28) \| (Range: 0 – 100) \| (Range: 1 – 12) \| \| **2** \| Retinopathy \| (Range: 0 – 100) \| (Range: 1 – 12) \| (Range: 0 – 100) \| (Range: 1 – 12) \| (Range: 1 – 12) \| (Range: 1 – 12) \| (Range: 0 – 100) \| (Range: 1 – 12) \| (Range: 1 – 14) \| (Range: 0 – 100) \| (Range: 1 – 5) \| (Range: 1 – 28) \| (Range: 0 – 100) \| (Range: 1 – 12) \| \| **3** \| Diabetic foot \| (Range: 0 – 100) \| (Range: 1 – 12) \| (Range: 0 – 100) \| (Range: 1 – 12) \| (Range: 1 – 12) \| (Range: 1 – 12) \| (Range: 0 – 100) \| (Range: 1 – 12) \| (Range: 1 – 14) \| (Range: 0 – 100) \| (Range: 1 – 5) \| (Range: 1 – 28) \| (Range: 0 – 100) \| (Range: 1 – 12) \| \| **4** \| Cardiovascular disease \| (Range: 0 – 100) \| (Range: 1 – 12) \| (Range: 0 – 100) \| (Range: 1 – 12) \| (Range: 1 – 12) \| (Range: 1 – 12) \| (Range: 0 – 100) \| (Range: 1 – 12) \| (Range: 1 – 14) \| (Range: 0 – 100) \| (Range: 1 – 5) \| (Range: 1 – 28) \| (Range: 0 – 100) \| (Range: 1 – 12) \| \| **5** \| Renal failure \| (Range: 0 – 100) \| (Range: 1 – 12) \| (Range: 0 – 100) \| (Range: 1 – 12) \| (Range: 1 – 12) \| (Range: 1 – 12) \| (Range: 0 – 100) \| (Range: 1 – 12) \| (Range: 1 – 14) \| (Range: 0 – 100) \| (Range: 1 – 5) \| (Range: 1 – 28) \| (Range: 0 – 100) \| (Range: 1 – 12) \| \| **5.1** \| Severe hyperglycemia/ketoacidosis **[FOR GR ONLY]** \| (Range: 0 – 100) \| (Range: 1 – 12) \| (Range: 0 – 100) \| (Range: 1 – 12) \| (Range: 1 – 12) \| (Range: 1 – 12) \| (Range: 0 – 100) \| (Range: 1 – 12) \| (Range: 1 – 14) \| (Range: 0 – 100) \| (Range: 1 – 5) \| (Range: 1 – 28) \| (Range: 0 – 100) \| (Range: 1 – 12) \| \|  \| **Asthma** \|  \|  \|  \|  \|  \|  \|  \|  \|  \|  \|  \|  \|  \|  \| \| **6** \| Asthma attack \| (Range: 0 – 100) \| (Range: 1 – 12) \| (Range: 0 – 100) \| (Range: 1 – 12) \| (Range: 1 – 12) \| (Range: 1 – 12) \| (Range: 0 – 100) \| (Range: 1 – 12) \| (Range: 1 – 14) \| (Range: 0 – 100) \| (Range: 1 – 5) \| (Range: 1 – 28) \| (Range: 0 – 100) \| (Range: 1 – 12) \| \| **6.1** \| Pulmonary infections  **[FOR GR ONLY]** \| (Range: 0 – 100) \| (Range: 1 – 12) \| (Range: 0 – 100) \| (Range: 1 – 12) \| (Range: 1 – 12) \| (Range: 1 – 12) \| (Range: 0 – 100) \| (Range: 1 – 12) \| (Range: 1 – 14) \| (Range: 0 – 100) \| (Range: 1 – 5) \| (Range: 1 – 28) \| (Range: 0 – 100) \| (Range: 1 – 12) \| \|  \| **Sleep apnea** \|  \|  \|  \|  \|  \|  \|  \|  \|  \|  \|  \|  \|  \|  \| \| **7** \| Cardiovascular complications (stroke, congestive heart failure, hypertension, coronary artery disease) \| (Range: 0 – 100) \| (Range: 1 – 12) \| (Range: 0 – 100) \| (Range: 1 – 12) \| (Range: 1 – 12) \| (Range: 1 – 12) \| (Range: 0 – 100) \| (Range: 1 – 12) \| (Range: 1 – 14) \| (Range: 0 – 100) \| (Range: 1 – 5) \| (Range: 1 – 28) \| (Range: 0 – 100) \| (Range: 1 – 12) \| \| **8** \| Metabolic syndrome, NAFLD or NASH \| (Range: 0 – 100) \| (Range: 1 – 12) \| (Range: 0 – 100) \| (Range: 1 – 12) \| (Range: 1 – 12) \| (Range: 1 – 12) \| (Range: 0 – 100) \| (Range: 1 – 12) \| (Range: 1 – 14) \| (Range: 0 – 100) \| (Range: 1 – 5) \| (Range: 1 – 28) \| (Range: 0 – 100) \| (Range: 1 – 12) \| \|  \| **Osteoarthritis** \|  \|  \|  \|  \|  \|  \|  \|  \|  \|  \|  \|  \|  \|  \| \| **9** \| Falls and fractures \| (Range: 0 – 100) \| (Range: 1 – 12) \| (Range: 0 – 100) \| (Range: 1 – 12) \| (Range: 1 – 12) \| (Range: 1 – 12) \| (Range: 0 – 100) \| (Range: 1 – 12) \| (Range: 1 – 14) \| (Range: 0 – 100) \| (Range: 1 – 5) \| (Range: 1 – 28) \| (Range: 0 – 100) \| (Range: 1 – 12) \| \| **10** \| Radiculopathies \| (Range: 0 – 100) \| (Range: 1 – 12) \| (Range: 0 – 100) \| (Range: 1 – 12) \| (Range: 1 – 12) \| (Range: 1 – 12) \| (Range: 0 – 100) \| (Range: 1 – 12) \| (Range: 1 – 14) \| (Range: 0 – 100) \| (Range: 1 – 5) \| (Range: 1 – 28) \| (Range: 0 – 100) \| (Range: 1 – 12) \| \| **11** \| Chronic pain, joint misalignment \| (Range: 0 – 100) \| (Range: 1 – 12) \| (Range: 0 – 100) \| (Range: 1 – 12) \| (Range: 1 – 12) \| (Range: 1 – 12) \| (Range: 0 – 100) \| (Range: 1 – 12) \| (Range: 1 – 14) \| (Range: 0 – 100) \| (Range: 1 – 5) \| (Range: 1 – 28) \| (Range: 0 – 100) \| (Range: 1 – 12) \| \|  \| **Heart failure** \|  \|  \|  \|  \|  \|  \|  \|  \|  \|  \|  \|  \|  \|  \| \| **12** \| Arrhythmia, thromboembolic events \| (Range: 0 – 100) \| (Range: 1 – 12) \| (Range: 0 – 100) \| (Range: 1 – 12) \| (Range: 1 – 12) \| (Range: 1 – 12) \| (Range: 0 – 100) \| (Range: 1 – 12) \| (Range: 1 – 14) \| (Range: 0 – 100) \| (Range: 1 – 5) \| (Range: 1 – 28) \| (Range: 0 – 100) \| (Range: 1 – 12) \| \| **13** \| Hepatic dysfunction, liver disease \| (Range: 0 – 100) \| (Range: 1 – 12) \| (Range: 0 – 100) \| (Range: 1 – 12) \| (Range: 1 – 12) \| (Range: 1 – 12) \| (Range: 0 – 100) \| (Range: 1 – 12) \| (Range: 1 – 14) \| (Range: 0 – 100) \| (Range: 1 – 5) \| (Range: 1 – 28) \| (Range: 0 – 100) \| (Range: 1 – 12) \| \| **14** \| Pulmonary edema \| (Range: 0 – 100) \| (Range: 1 – 12) \| (Range: 0 – 100) \| (Range: 1 – 12) \| (Range: 1 – 12) \| (Range: 1 – 12) \| (Range: 0 – 100) \| (Range: 1 – 12) \| (Range: 1 – 14) \| (Range: 0 – 100) \| (Range: 1 – 5) \| (Range: 1 – 28) \| (Range: 0 – 100) \| (Range: 1 – 12) \| \| **15** \| End stage renal disease \| (Range: 0 – 100) \| (Range: 1 – 12) \| (Range: 0 – 100) \| (Range: 1 – 12) \| (Range: 1 – 12) \| (Range: 1 – 12) \| (Range: 0 – 100) \| (Range: 1 – 12) \| (Range: 1 – 14) \| (Range: 0 – 100) \| (Range: 1 – 5) \| (Range: 1 – 28) \| (Range: 0 – 100) \| (Range: 1 – 12) \| \|  \| **Hypertension** \|  \|  \|  \|  \|  \|  \|  \|  \|  \|  \|  \|  \|  \|  \| \| **16** \| Central retinal artery occlusion, ophthalmologic complications \| (Range: 0 – 100) \| (Range: 1 – 12) \| (Range: 0 – 100) \| (Range: 1 – 12) \| (Range: 1 – 12) \| (Range: 1 – 12) \| (Range: 0 – 100) \| (Range: 1 – 12) \| (Range: 1 – 14) \| (Range: 0 – 100) \| (Range: 1 – 5) \| (Range: 1 – 28) \| (Range: 0 – 100) \| (Range: 1 – 12) \| \| **17** \| Atrial Fibrillation, thromboembolic events \| (Range: 0 – 100) \| (Range: 1 – 12) \| (Range: 0 – 100) \| (Range: 1 – 12) \| (Range: 1 – 12) \| (Range: 1 – 12) \| (Range: 0 – 100) \| (Range: 1 – 12) \| (Range: 1 – 14) \| (Range: 0 – 100) \| (Range: 1 – 5) \| (Range: 1 – 28) \| (Range: 0 – 100) \| (Range: 1 – 12) \| \| **18** \| MI, stroke, heart failure \| (Range: 0 – 100) \| (Range: 1 – 12) \| (Range: 0 – 100) \| (Range: 1 – 12) \| (Range: 1 – 12) \| (Range: 1 – 12) \| (Range: 0 – 100) \| (Range: 1 – 12) \| (Range: 1 – 14) \| (Range: 0 – 100) \| (Range: 1 – 5) \| (Range: 1 – 28) \| (Range: 0 – 100) \| (Range: 1 – 12) \| \| **19** \| Hepatic congestion and dysfunction \| (Range: 0 – 100) \| (Range: 1 – 12) \| (Range: 0 – 100) \| (Range: 1 – 12) \| (Range: 1 – 12) \| (Range: 1 – 12) \| (Range: 0 – 100) \| (Range: 1 – 12) \| (Range: 1 – 14) \| (Range: 0 – 100) \| (Range: 1 – 5) \| (Range: 1 – 28) \| (Range: 0 – 100) \| (Range: 1 – 12) \| \|  \| **Dyslipidemia** \|  \|  \|  \|  \|  \|  \|  \|  \|  \|  \|  \|  \|  \|  \| \| **20** \| Atherosclerosis \| (Range: 0 – 100) \| (Range: 1 – 12) \| (Range: 0 – 100) \| (Range: 1 – 12) \| (Range: 1 – 12) \| (Range: 1 – 12) \| (Range: 0 – 100) \| (Range: 1 – 12) \| (Range: 1 – 14) \| (Range: 0 – 100) \| (Range: 1 – 5) \| (Range: 1 – 28) \| (Range: 0 – 100) \| (Range: 1 – 12) \| \| **21** \| Peripheral arterial disease \| (Range: 0 – 100) \| (Range: 1 – 12) \| (Range: 0 – 100) \| (Range: 1 – 12) \| (Range: 1 – 12) \| (Range: 1 – 12) \| (Range: 0 – 100) \| (Range: 1 – 12) \| (Range: 1 – 14) \| (Range: 0 – 100) \| (Range: 1 – 5) \| (Range: 1 – 28) \| (Range: 0 – 100) \| (Range: 1 – 12) \| \| **22** \| Coronary artery disease \| (Range: 0 – 100) \| (Range: 1 – 12) \| (Range: 0 – 100) \| (Range: 1 – 12) \| (Range: 1 – 12) \| (Range: 1 – 12) \| (Range: 0 – 100) \| (Range: 1 – 12) \| (Range: 1 – 14) \| (Range: 0 – 100) \| (Range: 1 – 5) \| (Range: 1 – 28) \| (Range: 0 – 100) \| (Range: 1 – 12) \| \| **23** \| Myocardial infarction \| (Range: 0 – 100) \| (Range: 1 – 12) \| (Range: 0 – 100) \| (Range: 1 – 12) \| (Range: 1 – 12) \| (Range: 1 – 12) \| (Range: 0 – 100) \| (Range: 1 – 12) \| (Range: 1 – 14) \| (Range: 0 – 100) \| (Range: 1 – 5) \| (Range: 1 – 28) \| (Range: 0 – 100) \| (Range: 1 – 12) \| \| **24** \| Stroke \| (Range: 0 – 100) \| (Range: 1 – 12) \| (Range: 0 – 100) \| (Range: 1 – 12) \| (Range: 1 – 12) \| (Range: 1 – 12) \| (Range: 0 – 100) \| (Range: 1 – 12) \| (Range: 1 – 14) \| (Range: 0 – 100) \| (Range: 1 – 5) \| (Range: 1 – 28) \| (Range: 0 – 100) \| (Range: 1 – 12) \| \|  \| **Atrial Fibrillation** \|  \|  \|  \|  \|  \|  \|  \|  \|  \|  \|  \|  \|  \|  \| \| **25** \| Stroke \| (Range: 0 – 100) \| (Range: 1 – 12) \| (Range: 0 – 100) \| (Range: 1 – 12) \| (Range: 1 – 12) \| (Range: 1 – 12) \| (Range: 0 – 100) \| (Range: 1 – 12) \| (Range: 1 – 14) \| (Range: 0 – 100) \| (Range: 1 – 5) \| (Range: 1 – 28) \| (Range: 0 – 100) \| (Range: 1 – 12) \| \| **26** \| Myocardial infarction \| (Range: 0 – 100) \| (Range: 1 – 12) \| (Range: 0 – 100) \| (Range: 1 – 12) \| (Range: 1 – 12) \| (Range: 1 – 12) \| (Range: 0 – 100) \| (Range: 1 – 12) \| (Range: 1 – 14) \| (Range: 0 – 100) \| (Range: 1 – 5) \| (Range: 1 – 28) \| (Range: 0 – 100) \| (Range: 1 – 12) \| \| **27** \| Venous thromboembolism (DVT, PE) \| (Range: 0 – 100) \| (Range: 1 – 12) \| (Range: 0 – 100) \| (Range: 1 – 12) \| (Range: 1 – 12) \| (Range: 1 – 12) \| (Range: 0 – 100) \| (Range: 1 – 12) \| (Range: 1 – 14) \| (Range: 0 – 100) \| (Range: 1 – 5) \| (Range: 1 – 28) \| (Range: 0 – 100) \| (Range: 1 – 12) \| \|  \| **Angina** \|  \|  \|  \|  \|  \|  \|  \|  \|  \|  \|  \|  \|  \|  \| \| **28** \| MI \| (Range: 0 – 100) \| (Range: 1 – 12) \| (Range: 0 – 100) \| (Range: 1 – 12) \| (Range: 1 – 12) \| (Range: 1 – 12) \| (Range: 0 – 100) \| (Range: 1 – 12) \| (Range: 1 – 14) \| (Range: 0 – 100) \| (Range: 1 – 5) \| (Range: 1 – 28) \| (Range: 0 – 100) \| (Range: 1 – 12) \| \| **29** \| Pulmonary edema \| (Range: 0 – 100) \| (Range: 1 – 12) \| (Range: 0 – 100) \| (Range: 1 – 12) \| (Range: 1 – 12) \| (Range: 1 – 12) \| (Range: 0 – 100) \| (Range: 1 – 12) \| (Range: 1 – 14) \| (Range: 0 – 100) \| (Range: 1 – 5) \| (Range: 1 – 28) \| (Range: 0 – 100) \| (Range: 1 – 12) \| \| **30** \| Stroke \| (Range: 0 – 100) \| (Range: 1 – 12) \| (Range: 0 – 100) \| (Range: 1 – 12) \| (Range: 1 – 12) \| (Range: 1 – 12) \| (Range: 0 – 100) \| (Range: 1 – 12) \| (Range: 1 – 14) \| (Range: 0 – 100) \| (Range: 1 – 5) \| (Range: 1 – 28) \| (Range: 0 – 100) \| (Range: 1 – 12) \| \|  \| **Chronic Kidney Disease** \|  \|  \|  \|  \|  \|  \|  \|  \|  \|  \|  \|  \|  \|  \| \| **31** \| Metabolic bone disease \| (Range: 0 – 100) \| (Range: 1 – 12) \| (Range: 0 – 100) \| (Range: 1 – 12) \| (Range: 1 – 12) \| (Range: 1 – 12) \| (Range: 0 – 100) \| (Range: 1 – 12) \| (Range: 1 – 14) \| (Range: 0 – 100) \| (Range: 1 – 5) \| (Range: 1 – 28) \| (Range: 0 – 100) \| (Range: 1 – 12) \| \| **32** \| Gout \| (Range: 0 – 100) \| (Range: 1 – 12) \| (Range: 0 – 100) \| (Range: 1 – 12) \| (Range: 1 – 12) \| (Range: 1 – 12) \| (Range: 0 – 100) \| (Range: 1 – 12) \| (Range: 1 – 14) \| (Range: 0 – 100) \| (Range: 1 – 5) \| (Range: 1 – 28) \| (Range: 0 – 100) \| (Range: 1 – 12) \| \| **33** \| Dialysis equilibrium syndrome \| (Range: 0 – 100) \| (Range: 1 – 12) \| (Range: 0 – 100) \| (Range: 1 – 12) \| (Range: 1 – 12) \| (Range: 1 – 12) \| (Range: 0 – 100) \| (Range: 1 – 12) \| (Range: 1 – 14) \| (Range: 0 – 100) \| (Range: 1 – 5) \| (Range: 1 – 28) \| (Range: 0 – 100) \| (Range: 1 – 12) \| \| **34** \| All-cause CVD (CHF, AFib, stroke, coronary heart disease, PAD) \| (Range: 0 – 100) \| (Range: 1 – 12) \| (Range: 0 – 100) \| (Range: 1 – 12) \| (Range: 1 – 12) \| (Range: 1 – 12) \| (Range: 0 – 100) \| (Range: 1 – 12) \| (Range: 1 – 14) \| (Range: 0 – 100) \| (Range: 1 – 5) \| (Range: 1 – 28) \| (Range: 0 – 100) \| (Range: 1 – 12) \| \| **35** \| Hypertension \| (Range: 0 – 100) \| (Range: 1 – 12) \| (Range: 0 – 100) \| (Range: 1 – 12) \| (Range: 1 – 12) \| (Range: 1 – 12) \| (Range: 0 – 100) \| (Range: 1 – 12) \| (Range: 1 – 14) \| (Range: 0 – 100) \| (Range: 1 – 5) \| (Range: 1 – 28) \| (Range: 0 – 100) \| (Range: 1 – 12) \| \| **36** \| Anemia \| (Range: 0 – 100) \| (Range: 1 – 12) \| (Range: 0 – 100) \| (Range: 1 – 12) \| (Range: 1 – 12) \| (Range: 1 – 12) \| (Range: 0 – 100) \| (Range: 1 – 12) \| (Range: 1 – 14) \| (Range: 0 – 100) \| (Range: 1 – 5) \| (Range: 1 – 28) \| (Range: 0 – 100) \| (Range: 1 – 12) \| |
| --- | --- | --- | --- | --- | --- | --- | --- | --- | --- | --- | --- | --- | --- | --- | --- | --- | --- | --- | --- | --- | --- | --- | --- | --- | --- | --- | --- | --- | --- | --- | --- | --- | --- | --- | --- | --- | --- | --- | --- | --- | --- | --- | --- | --- | --- | --- | --- | --- | --- | --- | --- | --- | --- | --- | --- | --- | --- | --- | --- | --- | --- | --- | --- | --- | --- | --- | --- | --- | --- | --- | --- | --- | --- | --- | --- | --- | --- | --- | --- | --- | --- | --- | --- | --- | --- | --- | --- | --- | --- | --- | --- | --- | --- | --- | --- | --- | --- | --- | --- | --- | --- | --- | --- | --- | --- | --- | --- | --- | --- | --- | --- | --- | --- | --- | --- | --- | --- | --- | --- | --- | --- | --- | --- | --- | --- | --- | --- | --- | --- | --- | --- | --- | --- | --- | --- | --- | --- | --- | --- | --- | --- | --- | --- | --- | --- | --- | --- | --- | --- | --- | --- | --- | --- | --- | --- | --- | --- | --- | --- | --- | --- | --- | --- | --- | --- | --- | --- | --- | --- | --- | --- | --- | --- | --- | --- | --- | --- | --- | --- | --- | --- | --- | --- | --- | --- | --- | --- | --- | --- | --- | --- | --- | --- | --- | --- | --- | --- | --- | --- | --- | --- | --- | --- | --- | --- | --- | --- | --- | --- | --- | --- | --- | --- | --- | --- | --- | --- | --- | --- | --- | --- | --- | --- | --- | --- | --- | --- | --- | --- | --- | --- | --- | --- | --- | --- | --- | --- | --- | --- | --- | --- | --- | --- | --- | --- | --- | --- | --- | --- | --- | --- | --- | --- | --- | --- | --- | --- | --- | --- | --- | --- | --- | --- | --- | --- | --- | --- | --- | --- | --- | --- | --- | --- | --- | --- | --- | --- | --- | --- | --- | --- | --- | --- | --- | --- | --- | --- | --- | --- | --- | --- | --- | --- | --- | --- | --- | --- | --- | --- | --- | --- | --- | --- | --- | --- | --- | --- | --- | --- | --- | --- | --- | --- | --- | --- | --- | --- | --- | --- | --- | --- | --- | --- | --- | --- | --- | --- | --- | --- | --- | --- | --- | --- | --- | --- | --- | --- | --- | --- | --- | --- | --- | --- | --- | --- | --- | --- | --- | --- | --- | --- | --- | --- | --- | --- | --- | --- | --- | --- | --- | --- | --- | --- | --- | --- | --- | --- | --- | --- | --- | --- | --- | --- | --- | --- | --- | --- | --- | --- | --- | --- | --- | --- | --- | --- | --- | --- | --- | --- | --- | --- | --- | --- | --- | --- | --- | --- | --- | --- | --- | --- | --- | --- | --- | --- | --- | --- | --- | --- | --- | --- | --- | --- | --- | --- | --- | --- | --- | --- | --- | --- | --- | --- | --- | --- | --- | --- | --- | --- | --- | --- | --- | --- | --- | --- | --- | --- | --- | --- | --- | --- | --- | --- | --- | --- | --- | --- | --- | --- | --- | --- | --- | --- | --- | --- | --- | --- | --- | --- | --- | --- | --- | --- | --- | --- | --- | --- | --- | --- | --- | --- | --- | --- | --- | --- | --- | --- | --- | --- | --- | --- | --- | --- | --- | --- | --- | --- | --- | --- | --- | --- | --- | --- | --- | --- | --- | --- | --- | --- | --- | --- | --- | --- | --- | --- | --- | --- | --- | --- | --- | --- | --- | --- | --- | --- | --- | --- | --- | --- | --- | --- | --- | --- | --- | --- | --- | --- | --- | --- | --- | --- | --- | --- | --- | --- | --- | --- | --- | --- | --- | --- | --- | --- | --- | --- | --- | --- | --- | --- | --- | --- | --- | --- | --- | --- | --- | --- | --- | --- | --- | --- | --- | --- | --- | --- | --- | --- | --- | --- | --- | --- | --- | --- | --- | --- | --- | --- | --- | --- | --- | --- | --- | --- | --- | --- | --- | --- | --- | --- | --- | --- | --- | --- | --- | --- | --- | --- | --- | --- | --- | --- | --- | --- | --- | --- | --- | --- | --- | --- | --- | --- | --- | --- | --- | --- | --- | --- | --- | --- | --- | --- | --- | --- | --- | --- | --- | --- | --- | --- | --- | --- | --- | --- | --- | --- | --- | --- | --- | --- | --- | --- | --- | --- | --- | --- | --- | --- | --- | --- | --- | --- | --- | --- | --- | --- | --- | --- | --- | --- | --- | --- | --- | --- | --- | --- | --- | --- | --- | --- | --- | --- | --- | --- | --- | --- | --- | --- | --- | --- | --- | --- | --- | --- | --- | --- | --- | --- | --- | --- | --- | --- | --- | --- | --- | --- | --- | --- | --- | --- | --- | --- | --- | --- | --- | --- | --- | --- | --- | --- | --- | --- | --- | --- | --- | --- | --- | --- | --- | --- | --- | --- | --- | --- | --- | --- | --- | --- | --- | --- | --- | --- | --- | --- | --- | --- | --- | --- | --- | --- | --- | --- | --- | --- | --- | --- | --- | --- | --- | --- | --- | --- | --- | --- | --- | --- | --- | --- | --- | --- | --- | --- | --- | --- | --- | --- | --- | --- | --- | --- | --- | --- | --- | --- | --- | --- | --- | --- | --- | --- | --- | --- | --- | --- | --- | --- | --- | --- | --- | --- | --- | --- | --- | --- | --- | --- | --- | --- | --- | --- | --- | --- |
| Q9 | **PIPE IN SELECTION FROM S1**  **SHOW GRID TABLE WITH PRE-FILLED RANGES, ALLOW RESPONDENTS TO OVERWRITE ON CELLS**  **SHOW PROMPT IF INPUT IS OUT OF RANGE SPECIFIED**  **DO NOT ALLOW part B to be entered if part A was CODED 0 for “% patients”**  **SKIP FOR ASTHMA AND HYPERTENSION**   1. Of those [insert comorbidity from S4] patients experiencing a complication related their condition (not treatment), what proportion require each of the following in-patient procedures?   **Please enter a percentage within the stated range**   1. Of those [insert comorbidity from S4] patients experiencing a complication in relation to their condition (not treatment), how many times will a typical patient require each of the following in-patient procedures in a year?   **Please enter a number within the stated range**   \|  \| **In-patient procedures** \| \| --- \| --- \| \|  \|  \| **% patients**  **(range in brackets)** \| **Number in 1 year**  **(range in brackets)** \| \|  \| **T2D** \|  \|  \| \| **1** \| Amputation of diabetic foot/leg \| (Range: 0 – 100) \| (Range: 1 – 2) \| \| **2** \| Cataract surgery \| (Range: 0 – 100) \| (Range: 1 – 2) \| \| **3** \| Dialysis \| (Range: 0 – 100) \| (Range: 1 – 365) \| \| **4** \| Kidney transplant \| (Range: 0 – 100) \| (Range: 1 – 2) \| \| **5** \| Implantation of stents **[FOR ROM ONLY]** \| (Range: 0 – 100) \| (Range: 1 – 2) \| \|  \| **Sleep apnea** \|  \|  \| \| **6** \| Tracheotomy \| (Range: 0 – 100) \| (Range: 1 – 3) \| \| **7** \| Bypass procedure e.g. septoplasty, functional rhinoplasty, nasal valve surgery, nasal polypectomy \| (Range: 0 – 100) \| (Range: 1 – 3) \| \| **8** \| Oral & nasal procedures e.g. Uvulopalatopharyngoplasty, palatal advancement, pharyngotonsillectomy, tonsillectomy, and excision of tori mandibularis \| (Range: 0 – 100) \| (Range: 1 – 3) \| \| **9** \| Hypopharyngeal procedures e.g. Tongue reduction, partial glossectomy, lingual tonsillectomy, and mandibular advancement \| (Range: 0 – 100) \| (Range: 1 – 3) \| \|  \| **Osteoarthritis** \|  \|  \| \| **10** \| Arthroscopic procedures \| (Range: 0 – 100) \| (Range: 1 – 3) \| \| **11** \| Decompressive procedures \| (Range: 0 – 100) \| (Range: 1 – 3) \| \| **12** \| Arthrodesis \| (Range: 0 – 100) \| (Range: 1 – 3) \| \| **13** \| Osteotomy \| (Range: 0 – 100) \| (Range: 1 – 3) \| \| **14** \| Joint replacement \| (Range: 0 – 100) \| (Range: 1 – 3) \| \|  \| **Heart failure** \|  \|  \| \| **15** \| Coronary artery bypass surgery \| (Range: 0 – 100) \| (Range: 1 – 3) \| \| **16** \| Heart valve repair or replacement \| (Range: 0 – 100) \| (Range: 1 – 3) \| \| **17** \| Cardioverter-defibrillator implantation \| (Range: 0 – 100) \| (Range: 1 – 3) \| \| **18** \| Biventricular pacemaker implantation \| (Range: 0 – 100) \| (Range: 1 – 3) \| \| **19** \| Ventricular assist device implantation \| (Range: 0 – 100) \| (Range: 1 – 3) \| \| **20** \| Heart transplant \| (Range: 0 – 100) \| (Range: 1 – 2) \| \|  \| **Dyslipidemia** \|  \|  \| \| **21** \| LDL apheresis **[N/A FOR ROM]** \| (Range: 0 – 100) \| (Range: 1 – 3) \| \| **22** \| Ileal bypass **[N/A FOR ROM & GR]** \| (Range: 0 – 100) \| (Range: 1 – 3) \| \| **23** \| Liver transplantation \| (Range: 0 – 100) \| (Range: 1 – 2) \| \|  \| **Atrial Fibrillation** \|  \|  \| \| **24** \| Cardioversion \| (Range: 0 – 100) \| (Range: 1 – 3) \| \| **25** \| AV junction ablation \| (Range: 0 – 100) \| (Range: 1 – 3) \| \| **26** \| Pacemaker implantation \| (Range: 0 – 100) \| (Range: 1 – 3) \| \| **27** \| Atrial flutter ablation \| (Range: 0 – 100) \| (Range: 1 – 3) \| \|  \| **Angina** \|  \|  \| \| **28** \| Percutaneous coronary intervention \| (Range: 0 – 100) \| (Range: 1 – 3) \| \| **29** \| Coronary artery bypass surgery \| (Range: 0 – 100) \| (Range: 1 – 3) \| \|  \| **Chronic Kidney Disease** \|  \|  \| \| **30** \| Blood transfusion \| (Range: 0 – 100) \| (Range: 1 – 24) \| \| **31** \| Kidney transplantation \| (Range: 0 – 100) \| (Range: 1 – 2) \| |

**PROGRAMMER, FOR CARDIOLOGISTS ONLY THEN PLEASE STATE THE FOLLOWING:**

We would now like you to complete a survey for a different comorbidity.

**PROGRAMMER, FOR CARDIOLOGISTS ONLY PLEASE repeat the survey five times until all 5 comorbidities have been asked in turn. please rotate the order the comorbidities are shown in each survey.**

**OTHERWISE, THANK AND CLOSE.**
