# Supplementary material for: The economic burden of obesity in 4 south-eastern European countries associated with obesity-related co-morbidities
Source: BMC Health Serv Res. 2024 Mar 19;24:354. doi: 10.1186/s12913-024-10840-4 (PMC10953276; doi:10.1186/s12913-024-10840-4)
Supplement: Supplementary file 2 — Supplementary Material 2 [file 12913_2024_10840_MOESM2_ESM.docx]

**Additional File 2 – Payer Questionnaire**

| **A: SCREENER** |
| --- |

| S0 | **ASK ALL, SINGLE CHOICE (S/C)**  Please indicate your country?  **Please select one only**   \| Hungary (HUN) \| **1** \| **CONTINUE** \| \| --- \| --- \| --- \| \| Romania (ROM) \| **2** \| \| Greece (GR) \| **3** \| \| Other, please specify \| **99** \| **TERMINATE** \| |
| --- | --- | --- | --- | --- | --- | --- | --- | --- | --- | --- | --- |
| S1 | **ASK ALL, S/C**  Please indicate your current role in your hospital/center.  **Please select one only**   \| Administrator \| **1** \| **CONTINUE** \| \| --- \| --- \| --- \| \| Procurement \| **2** \| \| Other, please specify \| **99** \| **TERMINATE** \| |
| S2 | **ASK ALL, RANGE 0-99**  How many years have you been in your current role?  **Please enter a number**   \| __________ Years \| **1** \| **TERMINATE IF <3, OR >30** \| \| --- \| --- \| --- \| |
| S3 | **ASK ALL, S/C**  In which of the following hospital settings do you work?  **Please select one only**  **FOR ROMANIA**   \| Public Municipal Hospital \| **1** \| **TERMINATE IF SELECT CODE 4 or 99** \| \| --- \| --- \| --- \| \| Public County Emergency Hospital \| **2** \| \| Public Town hospital \| **3** \| \| Private hospital \| **4** \| \| Other, please specify \| **99** \|     **FOR HUNGARY**   \| Clinical centres \| **1** \| **TERMINATE IF SELECT CODE 8 or 99** \| \| --- \| --- \| --- \| \| Municipal (city) hospital \| **2** \| \| County hospital \| **3** \| \| National specialist institute \| **4** \| \| Specialist hospital/clinics \| **5** \| \| University/teaching hospital \| **6** \| \| Military/homeland defence hospital \| **7** \| \| Private hospital/clinic \| **8** \| \| Other, please specify \| **99** \|   **FOR GREECE**   \| Private hospitals \| **1** \| **TERMINATE IF SELECT CODE 99**  **Note: Only approach PRIVATE hospital setting for payer survey in Greece but ask for PUBLIC tariffs**  **INDICATIVE LIST FOR PRIVATE HOSPITALS: Metropolitan Hospital, Ygeia Hospital, Athens Medical Group (Iatriko)**  **INDICATIVE LIST FOR PRIVATE DIAGNOSTIC CENTRES: Bioaitriki, Affidea** \| \| --- \| --- \| --- \| \| Private diagnostic centres \| **2** \| \| Others \| **99** \| |
| S4 | **ASK ALL, S/C**  How would you rate your knowledge about costs of each of the following in your hospital?  **Please rate on a scale of 1-7 where 1 is no knowledge and 7 is highly knowledgeable.**   \|  \| \| No knowledge  of costs \| \| \|  \| Highly knowledgeable about costs \| \| \| \| --- \| --- \| --- \| --- \| --- \| --- \| --- \| --- \| --- \| \| 1 \| 2 \| 3 \| 4 \| 5 \| 6 \| 7 \| \| Medical consultations \| **A** \| ○ \| ○ \| ○ \| ○ \| ○ \| ○ \| ○ \| \| Health education programmes \| **B** \| ○ \| ○ \| ○ \| ○ \| ○ \| ○ \| ○ \| \| Medical tests \| **C** \| ○ \| ○ \| ○ \| ○ \| ○ \| ○ \| ○ \| \| Drugs, treatments, and medical devices \| **D** \| ○ \| ○ \| ○ \| ○ \| ○ \| ○ \| ○ \| \| Hospital stays \| **E** \| ○ \| ○ \| ○ \| ○ \| ○ \| ○ \| ○ \| \| Medical procedures \| **F** \| ○ \| ○ \| ○ \| ○ \| ○ \| ○ \| ○ \|     **IF RESPONDENT SELECTS <4 FOR ANY CODES A-F THEN TERMINATE, OTHERWISE CONTINUE** |

**END OF SCREENER**

| **B: MAIN QUESTIONNAIRE** |
| --- |

This survey is focused on public cost/price of managing and treating obesity related comorbidities**.** We would therefore like you to provide each of these costs /prices as accurately as possible. If you do not know the actual cost/price, please provide your best reasonable estimate.

| Q1 | **ASK IF SELECTING >4 AT S4 CODE A**  **SHOW GRID TABLE WITH PRE-FILLED RANGES, ALLOW RESPONDENTS TO OVERWRITE ON CELLS**  **SHOW PROMPT IF INPUT IS OUT OF RANGE SPECIFIED**  **SHOW PROMPT FOR COMMENT IF VALUE IS CHANGED**   1. What is the unit **[**cost **– if selecting S3 codes 1-6 /** price **– if selecting S3 code 7]** of each of the following medical consultations in your hospital?  **RANGE: 0.5x to 5x pre-populated value**   The values pre-populated below are estimates obtained from the following sources:  **NEAK (without valid social security card) [FOR HUN ONLY]**  **Ministry of Health, Greece (2012 values) [FOR GR ONLY]**  **Ministry of Health, Romania (**[**http://legislatie.just.ro/Public/DetaliiDocumentAfis/247076**](http://legislatie.just.ro/Public/DetaliiDocumentAfis/247076)**) [FOR ROM ONLY]**  **Please change the values if they are incorrect or inaccurate or suggest an appropriate value for missing costs.**   \|  \| **Consultations** \| **Unit cost** **/ price**  **(Range: 0.5x to 5x value shown)** \| \| --- \| --- \| --- \| \| **1** \| Cardiologist consultation \| [Insert pre-populated value here] \| \| **2** \| Chiropractor consultation **[N/A FOR GR]** \| [Insert pre-populated value here] \| \| **3** \| Diabetologist consultation \| [Insert pre-populated value here] \| \| **4** \| Endocrinologist consultation \| [Insert pre-populated value here] \| \| **5** \| ENT/Otolaryngologist consultation \| [Insert pre-populated value here] \| \| **6** \| Primary care physician/GP consultation \| [Insert pre-populated value here] \| \| **7** \| Internist consultation \| [Insert pre-populated value here] \| \| **8** \| Nephrologist consultation \| [Insert pre-populated value here] \| \| **9** \| Nurse consultation **[N/A FOR GR]** \| [Insert pre-populated value here] \| \| **10** \| Orthopedist consultation \| [Insert pre-populated value here] \| \| **11** \| Osteopath consultation **[N/A FOR GR]** \| [Insert pre-populated value here] \| \| **12** \| Physiotherapist consultation \| [Insert pre-populated value here] \| \| **13** \| Pulmonologist consultation \| [Insert pre-populated value here] \| |
| --- | --- | --- | --- | --- | --- | --- | --- | --- | --- | --- | --- | --- | --- | --- | --- | --- | --- | --- | --- | --- | --- | --- | --- | --- | --- | --- | --- | --- | --- | --- | --- | --- | --- | --- | --- | --- | --- | --- | --- | --- | --- | --- | --- |
| Q2 | **ASK IF SELECTING >4 AT S4 CODE B**  **SHOW GRID TABLE WITH PRE-FILLED RANGES, ALLOW RESPONDENTS TO OVERWRITE ON CELLS**  **SHOW PROMPT IF INPUT IS OUT OF RANGE SPECIFIED**  **N/A FOR GREECE, SKIP Q2**   1. What is the unit cost/price for attending each of the following health education programmes in your hospital? **RANGE: 0.5x to 5x pre-populated value**   The values pre-populated below are estimates obtained from the following sources  **[INSERT COUNTRY SPECIFIC SOURCE HERE FROM SECONDARY RESEARCH]**:  **Please change the values if they are incorrect or inaccurate or suggest an appropriate value for missing costs.**   \|  \| **Health education programmes** \| **Unit cost/price**  **(Range: 0.5x to 5x value shown)** \| \| --- \| --- \| --- \| \| **1** \| Hypertension disease education \| [Insert pre-populated value here] \| \| **2** \| Smoking cessation \| [Insert pre-populated value here] \| \| **3** \| T2D disease education \| [Insert pre-populated value here] \| \| **4** \| Weight loss \| [Insert pre-populated value here] \| |
| Q3 | **ASK IF SELECTING >4 AT S4 CODE C**  **SHOW GRID TABLE WITH PRE-FILLED RANGES, ALLOW RESPONDENTS TO OVERWRITE ON CELLS**  **SHOW PROMPT IF INPUT IS OUT OF RANGE SPECIFIED**   1. What is the unit cost/price of each of the following medical tests in your hospital?   **RANGE: 0.5x to 5x pre-populated value**  The values pre-populated below are estimates obtained from the following sources:  NEAK (without valid social security card) **[FOR HUN ONLY]**  Ministry of Health, Romania (<http://legislatie.just.ro/Public/DetaliiDocumentAfis/247076>) **[FOR ROM ONLY]**  **Please change the values if they are incorrect or inaccurate or suggest an appropriate value for missing costs.**   \|  \| **Medical tests** \| **Unit cost/price**  **(Range: 0.5x to 5x value shown)** \| \| --- \| --- \| --- \| \| **1** \| 12-L ECG \| [Insert pre-populated value here] \| \| **2** \| ANA/ Antinuclear antibodies \| [Insert pre-populated value here] \| \| **3** \| Ankle-brachial pressure index/ankle-brachial index **[FOR RO only]** \| [Insert pre-populated value here] \| \| **4** \| Attended, in-laboratory polysomnography \| [Insert pre-populated value here] \| \| **5** \| Bedside echocardiogram \| [Insert pre-populated value here] \| \| **6** \| B-type natriuretic peptide(BNP)/ N-terminal pro B-type natriuretic peptide (NT-proBNP) \| [Insert pre-populated value here] \| \| **7** \| BUN, Creatinine, GFR \| [Insert pre-populated value here] \| \| **8** \| Cardiac biomarker test \| [Insert pre-populated value here] \| \| **9** \| Cardiac catheterization **[N/A for HUN]** \| [Insert pre-populated value here] \| \| **10** \| Chest X-ray \| [Insert pre-populated value here] \| \| **11** \| Complete blood count \| [Insert pre-populated value here] \| \| **12** \| Coronary angiography \| [Insert pre-populated value here] \| \| **13** \| Creatine phosphokinase \| [Insert pre-populated value here] \| \| **14** \| ECG / Electrocardiogram \| [Insert pre-populated value here] \| \| **14.1** \| Electromyogram for diabetic neuropathy **[FOR GR ONLY]** \| [Insert pre-populated value here] \| \| **15** \| Erythrocyte sedimentation rate \| [Insert pre-populated value here] \| \| **16** \| Estimated glomerular filtration rate (eGFR) \| [Insert pre-populated value here] \| \| **17** \| Exhaled nitric oxide **[N/A for HUN]** \| [Insert pre-populated value here] \| \| **18** \| Fasting Plasma Glucose \| [Insert pre-populated value here] \| \| **19** \| Fasting serum triglycerides \| [Insert pre-populated value here] \| \| **20** \| Fundoscopy for diabetic neuropathy \| [Insert pre-populated value here] \| \| **21** \| Fundoscopy for diabetic retinopathy **[FOR RO only]** \| [Insert pre-populated value here] \| \| **22** \| Glycated hemoglobin (HbA1C) \| [Insert pre-populated value here] \| \| **23** \| Kidney biopsy \| [Insert pre-populated value here] \| \| **24** \| Lipid panel \| [Insert pre-populated value here] \| \| **24.1** \| Lipid profile for diabetic dyslipidemia **[FOR GR ONLY]** \| [Insert pre-populated value here] \| \| **25** \| Liver enzymes \| [Insert pre-populated value here] \| \| **26** \| Liver function test \| [Insert pre-populated value here] \| \| **27** \| Metabolic panel \| [Insert pre-populated value here] \| \| **28** \| MRI / Magnetic resonance imaging \| [Insert pre-populated value here] \| \| **29** \| Oral glucose tolerance test \| [Insert pre-populated value here] \| \| **30** \| Overnight pulse oximetry (1 night) \| [Insert pre-populated value here] \| \| **31** \| Peripheral eosinophilia and elevated IgE level \| [Insert pre-populated value here] \| \| **32** \| Portable monitoring (sleep apnea - 1 day) \| [Insert pre-populated value here] \| \| **33** \| Portable monitoring of cardiopulmonary channels (1 day) \| [Insert pre-populated value here] \| \| **34** \| Prothrombin test \| [Insert pre-populated value here] \| \| **35** \| Random Plasma Glucose \| [Insert pre-populated value here] \| \| **36** \| Renal function test \| [Insert pre-populated value here] \| \| **37** \| Renal ultrasound \| [Insert pre-populated value here] \| \| **38** \| Rheumatoid factor \| [Insert pre-populated value here] \| \| **39** \| Serum creatinine \| [Insert pre-populated value here] \| \| **40** \| Serum electrolytes \| [Insert pre-populated value here] \| \| **41** \| Serum total cholesterol, low-density lipoprotein cholesterol, high-density lipoprotein cholesterol \| [Insert pre-populated value here] \| \| **42** \| Serum uric acid \| [Insert pre-populated value here] \| \| **43** \| Skin prick and radioallergosorbent test (RAST) \| [Insert pre-populated value here] \| \| **44** \| Spiral CT scan **[N/A for HUN AND ROM]** \| [Insert pre-populated value here] \| \| **45** \| Spirometry \| [Insert pre-populated value here] \| \| **46** \| Synovial fluid analysis \| [Insert pre-populated value here] \| \| **47** \| Thyroid function test \| [Insert pre-populated value here] \| \| **48** \| Transesophageal echocardiogram \| [Insert pre-populated value here] \| \| **49** \| Transthoracic echocardiogram \| [Insert pre-populated value here] \| \| **50** \| Two-hour Oral Glucose Tolerance Test \| [Insert pre-populated value here] \| \| **50.1** \| UACR measurement for diabetic retinopathy **[FOR GR ONLY]** \| [Insert pre-populated value here] \| \| **51** \| Ultrasound for diabetic neuropathy \| [Insert pre-populated value here] \| \| **52** \| Ultrasound for peripheral artery **[FOR RO only]** \| [Insert pre-populated value here] \| \| **53** \| Urinalysis \| [Insert pre-populated value here] \| \| **54** \| Urinalysis, including test for microalbuminuria \| [Insert pre-populated value here] \| \| **55** \| X-ray \| [Insert pre-populated value here] \| |
| Q4 | **ASK IF SELECTING >4 AT S4 CODE D**  **SHOW GRID TABLE WITH PRE-FILLED RANGES, ALLOW RESPONDENTS TO OVERWRITE ON CELLS**  **SHOW PROMPT IF INPUT IS OUT OF RANGE SPECIFIED**  **N/A FOR GREECE, SKIP Q4**   1. What is the cost/price each of each of the following drugs in your hospital? **RANGE: 0.5x to 5x pre-populated value**   The values pre-populated below are estimates obtained from the following sources:  Nemzeti Egészségbiztosítási Alapkezelő - Végleges PUPHA (gov.hu) **[FOR HUN ONLY]**  Ministry of Health, Romania (<http://legislatie.just.ro/Public/DetaliiDocumentAfis/247076>) **[FOR ROM ONLY]**  <http://cas.cnas.ro/casmb/media/pageFiles/20211005_ORDIN%20Nr.%20887%20din%2027.09.2021-metodologiei%20de%20stabilire%20a%20preturilor%20de%20referinta.pdf> **[FOR ROM ONLY]**  **Please change the values if they are incorrect or inaccurate or suggest an appropriate value for missing costs.**   1. Please indicate if the cost/price mentioned is for a branded or generic drug  \|  \| **Treatments** \| **Pack strength** \| **Pack size** \| **[Cost** **– if selecting S3 codes 1-6 / Price** **– if selecting S3 code 7]** \| **Brand**  **S/C** \| **Generic**  **S/C** \| \| --- \| --- \| --- \| --- \| --- \| --- \| --- \| \| **1** \| Acetaminophen/ paracetamol \| [Insert pre-populated value here] \| [Insert pre-populated value here] \| [Insert pre-populated value here] \|  \|  \| \| **2** \| **Aldosterone antagonist** \|  \|  \|  \|  \|  \| \| **i** \| Spironolactone \| [Insert pre-populated value here] \| [Insert pre-populated value here] \| [Insert pre-populated value here] \|  \|  \| \| **ii** \| Eplerenone \| [Insert pre-populated value here] \| [Insert pre-populated value here] \| [Insert pre-populated value here] \|  \|  \| \| **3** \| **Alpha blockers e.g** \|  \|  \|  \|  \|  \| \| **i** \| Doxazosin \| [Insert pre-populated value here] \| [Insert pre-populated value here] \| [Insert pre-populated value here] \|  \|  \| \| **ii** \| Prazosin \| [Insert pre-populated value here] \| [Insert pre-populated value here] \| [Insert pre-populated value here] \|  \|  \| \| **iii** \| Terazosin \| [Insert pre-populated value here] \| [Insert pre-populated value here] \| [Insert pre-populated value here] \|  \|  \| \| **4** \| **Angiotensin converting enzyme inhibitors** \|  \|  \|  \|  \|  \| \| **i** \| Captopril \| [Insert pre-populated value here] \| [Insert pre-populated value here] \| [Insert pre-populated value here] \|  \|  \| \| **ii** \| Kinapril \| [Insert pre-populated value here] \| [Insert pre-populated value here] \| [Insert pre-populated value here] \|  \|  \| \| **iii** \| Enalapril \| [Insert pre-populated value here] \| [Insert pre-populated value here] \| [Insert pre-populated value here] \|  \|  \| \| **iv** \| Benazepril \| [Insert pre-populated value here] \| [Insert pre-populated value here] \| [Insert pre-populated value here] \|  \|  \| \| **v** \| Zofenopril \| [Insert pre-populated value here] \| [Insert pre-populated value here] \| [Insert pre-populated value here] \|  \|  \| \| **vi** \| Fosinopril \| [Insert pre-populated value here] \| [Insert pre-populated value here] \| [Insert pre-populated value here] \|  \|  \| \| **vii** \| Trandolapril \| [Insert pre-populated value here] \| [Insert pre-populated value here] \| [Insert pre-populated value here] \|  \|  \| \| **viii** \| Cilazapril \| [Insert pre-populated value here] \| [Insert pre-populated value here] \| [Insert pre-populated value here] \|  \|  \| \| **ix** \| Lisinopril \| [Insert pre-populated value here] \| [Insert pre-populated value here] \| [Insert pre-populated value here] \|  \|  \| \| **x** \| Perindopril \| [Insert pre-populated value here] \| [Insert pre-populated value here] \| [Insert pre-populated value here] \|  \|  \| \| **xi** \| Ramipril \| [Insert pre-populated value here] \| [Insert pre-populated value here] \| [Insert pre-populated value here] \|  \|  \| \| **5** \| **Angiotensin receptor blockers** \|  \|  \|  \|  \|  \| \| **i** \| Valsartan \| [Insert pre-populated value here] \| [Insert pre-populated value here] \| [Insert pre-populated value here] \|  \|  \| \| **ii** \| Losartan \| [Insert pre-populated value here] \| [Insert pre-populated value here] \| [Insert pre-populated value here] \|  \|  \| \| **iii** \| Candesartan \| [Insert pre-populated value here] \| [Insert pre-populated value here] \| [Insert pre-populated value here] \|  \|  \| \| **iv** \| Irbesartan \| [Insert pre-populated value here] \| [Insert pre-populated value here] \| [Insert pre-populated value here] \|  \|  \| \| **v** \| Telmisartan \| [Insert pre-populated value here] \| [Insert pre-populated value here] \| [Insert pre-populated value here] \|  \|  \| \| **vi** \| Olmesartan \| [Insert pre-populated value here] \| [Insert pre-populated value here] \| [Insert pre-populated value here] \|  \|  \| \| **vii** \| Eprosartan \| [Insert pre-populated value here] \| [Insert pre-populated value here] \| [Insert pre-populated value here] \|  \|  \| \| **6** \| **Antiarrhythmics** \|  \|  \|  \|  \|  \| \| **i** \| Amiodarone \| [Insert pre-populated value here] \| [Insert pre-populated value here] \| [Insert pre-populated value here] \|  \|  \| \| **ii** \| Flecainide \| [Insert pre-populated value here] \| [Insert pre-populated value here] \| [Insert pre-populated value here] \|  \|  \| \| **iii** \| Lidocaine \| [Insert pre-populated value here] \| [Insert pre-populated value here] \| [Insert pre-populated value here] \|  \|  \| \| **7** \| Acetylsalicylic acid (Aspirin) \| [Insert pre-populated value here] \| [Insert pre-populated value here] \| [Insert pre-populated value here] \|  \|  \| \| **8** \| **Asthma biologics** \|  \|  \|  \|  \|  \| \| **i** \| Omalizumab (Xolair) \| [Insert pre-populated value here] \| [Insert pre-populated value here] \| [Insert pre-populated value here] \|  \|  \| \| **ii** \| Mepolizumab (Nucala) \| [Insert pre-populated value here] \| [Insert pre-populated value here] \| [Insert pre-populated value here] \|  \|  \| \| **iii** \| Reslizumab (Cinqair) \| [Insert pre-populated value here] \| [Insert pre-populated value here] \| [Insert pre-populated value here] \|  \|  \| \| **iv** \| Benralizumab (Fasenra) \| [Insert pre-populated value here] \| [Insert pre-populated value here] \| [Insert pre-populated value here] \|  \|  \| \| **v** \| Dupilumab (Dupixent) \| [Insert pre-populated value here] \| [Insert pre-populated value here] \| [Insert pre-populated value here] \|  \|  \| \| **9** \| **Beta blockers** \|  \|  \|  \|  \|  \| \| **i** \| Metoprolol \| [Insert pre-populated value here] \| [Insert pre-populated value here] \| [Insert pre-populated value here] \|  \|  \| \| **ii** \| Karvedilol \| [Insert pre-populated value here] \| [Insert pre-populated value here] \| [Insert pre-populated value here] \|  \|  \| \| **iii** \| Nebivolol \| [Insert pre-populated value here] \| [Insert pre-populated value here] \| [Insert pre-populated value here] \|  \|  \| \| **iv** \| Bisoprolol \| [Insert pre-populated value here] \| [Insert pre-populated value here] \| [Insert pre-populated value here] \|  \|  \| \| **v** \| Atenolol \| [Insert pre-populated value here] \| [Insert pre-populated value here] \| [Insert pre-populated value here] \|  \|  \| \| **vi** \| Pindolol \| [Insert pre-populated value here] \| [Insert pre-populated value here] \| [Insert pre-populated value here] \|  \|  \| \| **vii** \| Acebutolol \| [Insert pre-populated value here] \| [Insert pre-populated value here] \| [Insert pre-populated value here] \|  \|  \| \| **viii** \| Propranolol \| [Insert pre-populated value here] \| [Insert pre-populated value here] \| [Insert pre-populated value here] \|  \|  \| \| **10** \| **Bile acid binding resins** \|  \|  \|  \|  \|  \| \| **i** \| Cholestyramine resin \| [Insert pre-populated value here] \| [Insert pre-populated value here] \| [Insert pre-populated value here] \|  \|  \| \| **ii** \| Colestipol hydrochloride \| [Insert pre-populated value here] \| [Insert pre-populated value here] \| [Insert pre-populated value here] \|  \|  \| \| **11** \| **B-selective agonist (long-acting, for asthma)** \|  \|  \|  \|  \|  \| \| **i** \| Salmeterol \| [Insert pre-populated value here] \| [Insert pre-populated value here] \| [Insert pre-populated value here] \|  \|  \| \| **ii** \| Formoterol \| [Insert pre-populated value here] \| [Insert pre-populated value here] \| [Insert pre-populated value here] \|  \|  \| \| **12** \| **B-selective agonist (short-acting, for asthma)** \|  \|  \|  \|  \|  \| \| **i** \| Albuterol/Salbutamol \| [Insert pre-populated value here] \| [Insert pre-populated value here] \| [Insert pre-populated value here] \|  \|  \| \| **ii** \| Levalbuterol/Levosalbutamol \| [Insert pre-populated value here] \| [Insert pre-populated value here] \| [Insert pre-populated value here] \|  \|  \| \| **13** \| **Calcium channel blockers** \|  \|  \|  \|  \|  \| \| **i** \| Amlodipin \| [Insert pre-populated value here] \| [Insert pre-populated value here] \| [Insert pre-populated value here] \|  \|  \| \| **ii** \| Benidipin \| [Insert pre-populated value here] \| [Insert pre-populated value here] \| [Insert pre-populated value here] \|  \|  \| \| **iii** \| Felodipin \| [Insert pre-populated value here] \| [Insert pre-populated value here] \| [Insert pre-populated value here] \|  \|  \| \| **iv** \| Lacidipine \| [Insert pre-populated value here] \| [Insert pre-populated value here] \| [Insert pre-populated value here] \|  \|  \| \| **v** \| Lercanidipine \| [Insert pre-populated value here] \| [Insert pre-populated value here] \| [Insert pre-populated value here] \|  \|  \| \| **vi** \| Nifedipin \| [Insert pre-populated value here] \| [Insert pre-populated value here] \| [Insert pre-populated value here] \|  \|  \| \| **vii** \| Nimodipin \| [Insert pre-populated value here] \| [Insert pre-populated value here] \| [Insert pre-populated value here] \|  \|  \| \| **viii** \| Diltiazem \| [Insert pre-populated value here] \| [Insert pre-populated value here] \| [Insert pre-populated value here] \|  \|  \| \| **ix** \| Verapamil \| [Insert pre-populated value here] \| [Insert pre-populated value here] \| [Insert pre-populated value here] \|  \|  \| \| **14** \| **Cholesterol absorption blockers** \|  \|  \|  \|  \|  \| \| **i** \| Ezetimibe \| [Insert pre-populated value here] \| [Insert pre-populated value here] \| [Insert pre-populated value here] \|  \|  \| \| **ii** \| Clopidogrel \| [Insert pre-populated value here] \| [Insert pre-populated value here] \| [Insert pre-populated value here] \|  \|  \| \| **15** \| **Corticosteroids (for asthma)** \|  \|  \|  \|  \|  \| \| **i** \| Beclomethasone \| [Insert pre-populated value here] \| [Insert pre-populated value here] \| [Insert pre-populated value here] \|  \|  \| \| **ii** \| Budesonide \| [Insert pre-populated value here] \| [Insert pre-populated value here] \| [Insert pre-populated value here] \|  \|  \| \| **iii** \| Ciclesonide \| [Insert pre-populated value here] \| [Insert pre-populated value here] \| [Insert pre-populated value here] \|  \|  \| \| **iv** \| Fluticasone \| [Insert pre-populated value here] \| [Insert pre-populated value here] \| [Insert pre-populated value here] \|  \|  \| \| **v** \| Mometasone \| [Insert pre-populated value here] \| [Insert pre-populated value here] \| [Insert pre-populated value here] \|  \|  \| \| **16** \| **Dipeptidyl peptidase 4 (DPPIV Inhibitors)** \|  \|  \|  \|  \|  \| \| **i** \| Sitagliptin (Januvia) \| [Insert pre-populated value here] \| [Insert pre-populated value here] \| [Insert pre-populated value here] \|  \|  \| \| **ii** \| Saxagliptin (Onglyza) \| [Insert pre-populated value here] \| [Insert pre-populated value here] \| [Insert pre-populated value here] \|  \|  \| \| **iii** \| Linagliptin (Tradjenta) \| [Insert pre-populated value here] \| [Insert pre-populated value here] \| [Insert pre-populated value here] \|  \|  \| \| **iv** \| Alogliptin (Nesina/Vipidia) \| [Insert pre-populated value here] \| [Insert pre-populated value here] \| [Insert pre-populated value here] \|  \|  \| \| **v** \| Vildagliptin (Galvus) \| [Insert pre-populated value here] \| [Insert pre-populated value here] \| [Insert pre-populated value here] \|  \|  \| \| **17** \| **Diuretics** \|  \|  \|  \|  \|  \| \| **i** \| Hydrochlorothiazide \| [Insert pre-populated value here] \| [Insert pre-populated value here] \| [Insert pre-populated value here] \|  \|  \| \| **ii** \| Acetazolamide \| [Insert pre-populated value here] \| [Insert pre-populated value here] \| [Insert pre-populated value here] \|  \|  \| \| **iii** \| Methazolamide \| [Insert pre-populated value here] \| [Insert pre-populated value here] \| [Insert pre-populated value here] \|  \|  \| \| **iv** \| Spironolactone \| [Insert pre-populated value here] \| [Insert pre-populated value here] \| [Insert pre-populated value here] \|  \|  \| \| **v** \| Amiloride \| [Insert pre-populated value here] \| [Insert pre-populated value here] \| [Insert pre-populated value here] \|  \|  \| \| **vi** \| Triamterene \| [Insert pre-populated value here] \| [Insert pre-populated value here] \| [Insert pre-populated value here] \|  \|  \| \| **vii** \| Mannitol \| [Insert pre-populated value here] \| [Insert pre-populated value here] \| [Insert pre-populated value here] \|  \|  \| \| **viii** \| Eplerenone \| [Insert pre-populated value here] \| [Insert pre-populated value here] \| [Insert pre-populated value here] \|  \|  \| \| **ix** \| Indapamide \| [Insert pre-populated value here] \| [Insert pre-populated value here] \| [Insert pre-populated value here] \|  \|  \| \| **x** \| Furosemide \| [Insert pre-populated value here] \| [Insert pre-populated value here] \| [Insert pre-populated value here] \|  \|  \| \| **18** \| Duloxetine \| [Insert pre-populated value here] \| [Insert pre-populated value here] \| [Insert pre-populated value here] \|  \|  \| \| **19** \| **Erythropoietin/Erythropoietin stimulating agents** \|  \|  \|  \|  \|  \| \| **i** \| Darbepoetin alfa \| [Insert pre-populated value here] \| [Insert pre-populated value here] \| [Insert pre-populated value here] \|  \|  \| \| **Ii** \| Epoetin alfa \| [Insert pre-populated value here] \| [Insert pre-populated value here] \| [Insert pre-populated value here] \|  \|  \| \| **Iii** \| Epoetin beta \| [Insert pre-populated value here] \| [Insert pre-populated value here] \| [Insert pre-populated value here] \|  \|  \| \| **Iv** \| Methoxy polyethylene glycol-epoetin beta \| [Insert pre-populated value here] \| [Insert pre-populated value here] \| [Insert pre-populated value here] \|  \|  \| \| **v** \| Epoetin zeta \| [Insert pre-populated value here] \| [Insert pre-populated value here] \| [Insert pre-populated value here] \|  \|  \| \| **20** \| **Fibric acid derivatives** \|  \|  \|  \|  \|  \| \| **i** \| Clofibrate \| [Insert pre-populated value here] \| [Insert pre-populated value here] \| [Insert pre-populated value here] \|  \|  \| \| **ii** \| Gemfibrozil \| [Insert pre-populated value here] \| [Insert pre-populated value here] \| [Insert pre-populated value here] \|  \|  \| \| **iii** \| Bezafibrate \| [Insert pre-populated value here] \| [Insert pre-populated value here] \| [Insert pre-populated value here] \|  \|  \| \| **iv** \| Ciprofibrate \| [Insert pre-populated value here] \| [Insert pre-populated value here] \| [Insert pre-populated value here] \|  \|  \| \| **v** \| Fenofibrate \| [Insert pre-populated value here] \| [Insert pre-populated value here] \| [Insert pre-populated value here] \|  \|  \| \| **21** \| **GLP-1 agonists** \|  \|  \|  \|  \|  \| \| **i** \| Dulaglutide (Trulicity) \| [Insert pre-populated value here] \| [Insert pre-populated value here] \| [Insert pre-populated value here] \|  \|  \| \| **ii** \| Exenatide (Byetta/Bydureon) \| [Insert pre-populated value here] \| [Insert pre-populated value here] \| [Insert pre-populated value here] \|  \|  \| \| **iii** \| Semaglutide (Ozempic) \| [Insert pre-populated value here] \| [Insert pre-populated value here] \| [Insert pre-populated value here] \|  \|  \| \| **iv** \| Liraglutide (Victoza) \| [Insert pre-populated value here] \| [Insert pre-populated value here] \| [Insert pre-populated value here] \|  \|  \| \| **v** \| Lixisenatide (Lyxumia) \| [Insert pre-populated value here] \| [Insert pre-populated value here] \| [Insert pre-populated value here] \|  \|  \| \| **22** \| **Glucocorticoids** \|  \|  \|  \|  \|  \| \| **i** \| Prednisolone \| [Insert pre-populated value here] \| [Insert pre-populated value here] \| [Insert pre-populated value here] \|  \|  \| \| **ii** \| Methylprednisolone \| [Insert pre-populated value here] \| [Insert pre-populated value here] \| [Insert pre-populated value here] \|  \|  \| \| **iii** \| Hydrocortisone \| [Insert pre-populated value here] \| [Insert pre-populated value here] \| [Insert pre-populated value here] \|  \|  \| \| **iv** \| Betamethasone \| [Insert pre-populated value here] \| [Insert pre-populated value here] \| [Insert pre-populated value here] \|  \|  \| \| **v** \| Dexamethasone \| [Insert pre-populated value here] \| [Insert pre-populated value here] \| [Insert pre-populated value here] \|  \|  \| \| **vi** \| Deflasacorte \| [Insert pre-populated value here] \| [Insert pre-populated value here] \| [Insert pre-populated value here] \|  \|  \| \| **vii** \| Fluocortolon \| [Insert pre-populated value here] \| [Insert pre-populated value here] \| [Insert pre-populated value here] \|  \|  \| \| **23** \| Heparin \| [Insert pre-populated value here] \| [Insert pre-populated value here] \| [Insert pre-populated value here] \|  \|  \| \| **24** \| Hyaluronic acid \| [Insert pre-populated value here] \| [Insert pre-populated value here] \| [Insert pre-populated value here] \|  \|  \| \| **25** \| **Hypoglycemic agents for glycemic control in CKD** \|  \|  \|  \|  \|  \| \| **i** \| Repaglinide \| [Insert pre-populated value here] \| [Insert pre-populated value here] \| [Insert pre-populated value here] \|  \|  \| \| **ii** \| Nateglinide \| [Insert pre-populated value here] \| [Insert pre-populated value here] \| [Insert pre-populated value here] \|  \|  \| \| **iii** \| Rosiglitazone \| [Insert pre-populated value here] \| [Insert pre-populated value here] \| [Insert pre-populated value here] \|  \|  \| \| **iv** \| Pioglitazone \| [Insert pre-populated value here] \| [Insert pre-populated value here] \| [Insert pre-populated value here] \|  \|  \| \| **26** \| **Imidazoline-I receptor agonists [FOR HUN ONLY]** \|  \|  \|  \|  \|  \| \| **i** \| Clonidine \| [Insert pre-populated value here] \| [Insert pre-populated value here] \| [Insert pre-populated value here] \|  \|  \| \| **ii** \| Guanfacine \| [Insert pre-populated value here] \| [Insert pre-populated value here] \| [Insert pre-populated value here] \|  \|  \| \| **iii** \| Moxonidine \| [Insert pre-populated value here] \| [Insert pre-populated value here] \| [Insert pre-populated value here] \|  \|  \| \| **iv** \| Rilmenidine \| [Insert pre-populated value here] \| [Insert pre-populated value here] \| [Insert pre-populated value here] \|  \|  \| \| **v** \| Tolonidine \| [Insert pre-populated value here] \| [Insert pre-populated value here] \| [Insert pre-populated value here] \|  \|  \| \| **27** \| Ivabradine **[FOR HUN ONLY]** \| [Insert pre-populated value here] \| [Insert pre-populated value here] \| [Insert pre-populated value here] \|  \|  \| \| **28** \| **Low Molecular Weight Heparin (LMWH)** \|  \|  \|  \|  \|  \| \| **I** \| Enoxaparin \| [Insert pre-populated value here] \| [Insert pre-populated value here] \| [Insert pre-populated value here] \|  \|  \| \| **Ii** \| Dalteparin \| [Insert pre-populated value here] \| [Insert pre-populated value here] \| [Insert pre-populated value here] \|  \|  \| \| **29** \| **Leukotriene receptor antagonist** \|  \|  \|  \|  \|  \| \| **I** \| Zafirlukast \| [Insert pre-populated value here] \| [Insert pre-populated value here] \| [Insert pre-populated value here] \|  \|  \| \| **Ii** \| Montelukast \| [Insert pre-populated value here] \| [Insert pre-populated value here] \| [Insert pre-populated value here] \|  \|  \| \| **30** \| **Leukotriene synthesis inhibitors** \|  \|  \|  \|  \|  \| \| **I** \| Zileuton (Zyflo) \| [Insert pre-populated value here] \| [Insert pre-populated value here] \| [Insert pre-populated value here] \|  \|  \| \| **31** \| **Long-acting insulins** \|  \|  \|  \|  \|  \| \| **i** \| Glargine (Lantus/Basaglar,Semglee) \| [Insert pre-populated value here] \| [Insert pre-populated value here] \| [Insert pre-populated value here] \|  \|  \| \| **ii** \| Glargine (Toujeo) \| [Insert pre-populated value here] \| [Insert pre-populated value here] \| [Insert pre-populated value here] \|  \|  \| \| **iii** \| Detemir (Levemir) \| [Insert pre-populated value here] \| [Insert pre-populated value here] \| [Insert pre-populated value here] \|  \|  \| \| **iv** \| Degludec (Tresiba) \| [Insert pre-populated value here] \| [Insert pre-populated value here] \| [Insert pre-populated value here] \|  \|  \| \| **32** \| **Mast cell stabilizers** \|  \|  \|  \|  \|  \| \| **I** \| Cromolyn sodium \| [Insert pre-populated value here] \| [Insert pre-populated value here] \| [Insert pre-populated value here] \|  \|  \| \| **33** \| Metformin/biguanides \| [Insert pre-populated value here] \| [Insert pre-populated value here] \| [Insert pre-populated value here] \|  \|  \| \| **34** \| **Methylxanthine** \|  \|  \|  \|  \|  \| \| **i** \| Teofillin \| [Insert pre-populated value here] \| [Insert pre-populated value here] \| [Insert pre-populated value here] \|  \|  \| \| **35** \| **Muscarinic receptor agonist (for asthma)** \|  \|  \|  \|  \|  \| \| **I** \| Acetylcholine \| [Insert pre-populated value here] \| [Insert pre-populated value here] \| [Insert pre-populated value here] \|  \|  \| \| **36** \| **NOACs/DOACs** \|  \|  \|  \|  \|  \| \| **i** \| Rivaroxaban \| [Insert pre-populated value here] \| [Insert pre-populated value here] \| [Insert pre-populated value here] \|  \|  \| \| **ii** \| Apixaban \| [Insert pre-populated value here] \| [Insert pre-populated value here] \| [Insert pre-populated value here] \|  \|  \| \| **iii** \| Edoxaban \| [Insert pre-populated value here] \| [Insert pre-populated value here] \| [Insert pre-populated value here] \|  \|  \| \| **iv** \| Dabigatran \| [Insert pre-populated value here] \| [Insert pre-populated value here] \| [Insert pre-populated value here] \|  \|  \| \| **37** \| **Nonsteroidal Anti-inflammatory Drugs (NSAIDS)** \|  \|  \|  \|  \|  \| \| **i** \| - Naproxen \| [Insert pre-populated value here] \| [Insert pre-populated value here] \| [Insert pre-populated value here] \|  \|  \| \| **ii** \| - Diclofenac \| [Insert pre-populated value here] \| [Insert pre-populated value here] \| [Insert pre-populated value here] \|  \|  \| \| **iii** \| - Celecoxib \| [Insert pre-populated value here] \| [Insert pre-populated value here] \| [Insert pre-populated value here] \|  \|  \| \| **iv** \| - Mefenamic acid \| [Insert pre-populated value here] \| [Insert pre-populated value here] \| [Insert pre-populated value here] \|  \|  \| \| **v** \| - Etoricoxib \| [Insert pre-populated value here] \| [Insert pre-populated value here] \| [Insert pre-populated value here] \|  \|  \| \| **vi** \| - Indomethacin \| [Insert pre-populated value here] \| [Insert pre-populated value here] \| [Insert pre-populated value here] \|  \|  \| \| **vii** \| - Rofecoxib \| [Insert pre-populated value here] \| [Insert pre-populated value here] \| [Insert pre-populated value here] \|  \|  \| \| **viii** \| - Parecoxib \| [Insert pre-populated value here] \| [Insert pre-populated value here] \| [Insert pre-populated value here] \|  \|  \| \| **ix** \| - Ibuprofen \| [Insert pre-populated value here] \| [Insert pre-populated value here] \| [Insert pre-populated value here] \|  \|  \| \| **38** \| **Opioids** \|  \|  \|  \|  \|  \| \| **i** \| Codeine \| [Insert pre-populated value here] \| [Insert pre-populated value here] \| [Insert pre-populated value here] \|  \|  \| \| **ii** \| Hydromorphone \| [Insert pre-populated value here] \| [Insert pre-populated value here] \| [Insert pre-populated value here] \|  \|  \| \| **iii** \| Oxycodone \| [Insert pre-populated value here] \| [Insert pre-populated value here] \| [Insert pre-populated value here] \|  \|  \| \| **iv** \| Morphine \| [Insert pre-populated value here] \| [Insert pre-populated value here] \| [Insert pre-populated value here] \|  \|  \| \| **v** \| Meperidine/Pethidine \| [Insert pre-populated value here] \| [Insert pre-populated value here] \| [Insert pre-populated value here] \|  \|  \| \| **vi** \| Tramadol \| [Insert pre-populated value here] \| [Insert pre-populated value here] \| [Insert pre-populated value here] \|  \|  \| \| **39** \| **PCSK9 inhibitors** \|  \|  \|  \|  \|  \| \| **I** \| Alirocumab (Praluent) \| [Insert pre-populated value here] \| [Insert pre-populated value here] \| [Insert pre-populated value here] \|  \|  \| \| **Ii** \| Evolocumab (Repatha) \| [Insert pre-populated value here] \| [Insert pre-populated value here] \| [Insert pre-populated value here] \|  \|  \| \| **40** \| **Phosphate binders** \|  \|  \|  \|  \|  \| \| **i** \| Sucroferric oxyhydroxide \| [Insert pre-populated value here] \| [Insert pre-populated value here] \| [Insert pre-populated value here] \|  \|  \| \| **ii** \| Sevelamer \| [Insert pre-populated value here] \| [Insert pre-populated value here] \| [Insert pre-populated value here] \|  \|  \| \| **iii** \| Ferric citrate \| [Insert pre-populated value here] \| [Insert pre-populated value here] \| [Insert pre-populated value here] \|  \|  \| \| **iv** \| Lanthanum carbonate \| [Insert pre-populated value here] \| [Insert pre-populated value here] \| [Insert pre-populated value here] \|  \|  \| \| **v** \| Calcium acetate \| [Insert pre-populated value here] \| [Insert pre-populated value here] \| [Insert pre-populated value here] \|  \|  \| \| **41** \| Probucol (Lorelco) **[N/A for HUN]** \| [Insert pre-populated value here] \| [Insert pre-populated value here] \| [Insert pre-populated value here] \|  \|  \| \| **42** \| **Neutral protamine Hagedorn insulins** \|  \|  \|  \|  \|  \| \| **I** \| Isophane \| [Insert pre-populated value here] \| [Insert pre-populated value here] \| [Insert pre-populated value here] \|  \|  \| \| **43** \| **Sodium-glucose cotransporter inhibitors** \|  \|  \|  \|  \|  \| \| **I** \| Canagliflozin (Invokana) \| [Insert pre-populated value here] \| [Insert pre-populated value here] \| [Insert pre-populated value here] \|  \|  \| \| **Ii** \| Dapagliflozin (Forxiga) \| [Insert pre-populated value here] \| [Insert pre-populated value here] \| [Insert pre-populated value here] \|  \|  \| \| **Iii** \| Empagliflozin (Jardiance) \| [Insert pre-populated value here] \| [Insert pre-populated value here] \| [Insert pre-populated value here] \|  \|  \| \| **44** \| **Short-acting insulins** \|  \|  \|  \|  \|  \| \| **i** \| Aspart (NovoRapid/Novolog/Fiasp) \| [Insert pre-populated value here] \| [Insert pre-populated value here] \| [Insert pre-populated value here] \|  \|  \| \| **ii** \| Lispro (Humalog) \| [Insert pre-populated value here] \| [Insert pre-populated value here] \| [Insert pre-populated value here] \|  \|  \| \| **45** \| **Statins** \|  \|  \|  \|  \|  \| \| **i** \| Atorvastatin \| [Insert pre-populated value here] \| [Insert pre-populated value here] \| [Insert pre-populated value here] \|  \|  \| \| **ii** \| Fluvastatin \| [Insert pre-populated value here] \| [Insert pre-populated value here] \| [Insert pre-populated value here] \|  \|  \| \| **iii** \| Lovastatin \| [Insert pre-populated value here] \| [Insert pre-populated value here] \| [Insert pre-populated value here] \|  \|  \| \| **iv** \| Pitavastatin \| [Insert pre-populated value here] \| [Insert pre-populated value here] \| [Insert pre-populated value here] \|  \|  \| \| **v** \| Pravastatin \| [Insert pre-populated value here] \| [Insert pre-populated value here] \| [Insert pre-populated value here] \|  \|  \| \| **vi** \| Rosuvastatin \| [Insert pre-populated value here] \| [Insert pre-populated value here] \| [Insert pre-populated value here] \|  \|  \| \| **vii** \| Simvastatin \| [Insert pre-populated value here] \| [Insert pre-populated value here] \| [Insert pre-populated value here] \|  \|  \| \| **46** \| **Sulfonylureas** \|  \|  \|  \|  \|  \| \| **i** \| Gliclazide \| [Insert pre-populated value here] \| [Insert pre-populated value here] \| [Insert pre-populated value here] \|  \|  \| \| **ii** \| Glimepiride \| [Insert pre-populated value here] \| [Insert pre-populated value here] \| [Insert pre-populated value here] \|  \|  \| \| **iii** \| Glipizide \| [Insert pre-populated value here] \| [Insert pre-populated value here] \| [Insert pre-populated value here] \|  \|  \| \| **iv** \| Tolbutamide \| [Insert pre-populated value here] \| [Insert pre-populated value here] \| [Insert pre-populated value here] \|  \|  \| \| **47** \| Trimetazidine **[FOR HUN ONLY]** \| [Insert pre-populated value here] \| [Insert pre-populated value here] \| [Insert pre-populated value here] \|  \|  \| \| **48** \| **Thiazolidinedione** \|  \|  \|  \|  \|  \| \| **i** \| Pioglitazone \| [Insert pre-populated value here] \| [Insert pre-populated value here] \| [Insert pre-populated value here] \|  \|  \| \| **ii** \| Rosiglitazone \| [Insert pre-populated value here] \| [Insert pre-populated value here] \| [Insert pre-populated value here] \|  \|  \| \| **49** \| Ticagrelor \| [Insert pre-populated value here] \| [Insert pre-populated value here] \| [Insert pre-populated value here] \|  \|  \| \| **50** \| Warfarin \| [Insert pre-populated value here] \| [Insert pre-populated value here] \| [Insert pre-populated value here] \|  \|  \| \| **51** \| **Pre-mixed insulin** \|  \|  \|  \|  \|  \| \| **i** \| Aspart & Protamine (Novomix) \| [Insert pre-populated value here] \| [Insert pre-populated value here] \| [Insert pre-populated value here] \|  \|  \| \| **ii** \| Degludec & Aspart (Ryzodeg) \| [Insert pre-populated value here] \| [Insert pre-populated value here] \| [Insert pre-populated value here] \|  \|  \| \| **52** \| **Basal and GLP-1 fixed ratio combinations** \|  \|  \|  \|  \|  \| \| **i** \| Degludec & Liraglutide (Xultophy) \| [Insert pre-populated value here] \| [Insert pre-populated value here] \| [Insert pre-populated value here] \|  \|  \| \| **ii** \| Glargine & Lixisenatide (Soliqua) \| 33 mcg/ml \| [Insert pre-populated value here] \| [Insert pre-populated value here] \|  \|  \| \| **iii** \| Glargine & Lixisenatide (Soliqua) \| 50 mcg/ml \| [Insert pre-populated value here] \| [Insert pre-populated value here] \|  \|  \| |
| Q4b | **ASK IF SELECTING >4 AT S4 CODE D**  **N/A FOR GREECE, SKIP Q4**   1. What do you estimate to be typical % cost/price increase for branded treatments vs. generics?   **Please enter a percentage increase for branded treatments**   \| **1** \| Branded treatment \| % more expensive \| \| --- \| --- \| --- \| |
| Q5 | **ASK IF SELECTING >4 AT S4 CODE D**  **SHOW GRID TABLE WITH PRE-FILLED RANGES, ALLOW RESPONDENTS TO OVERWRITE ON CELLS**  **SHOW PROMPT IF INPUT IS OUT OF RANGE SPECIFIED**   1. What is the cost/price of each of each of the following consumables/devices in your hospital? **RANGE: 0.5x to 5x pre-populated value**   The values pre-populated below are estimates obtained from the following sources:  NEAK (without valid social security card) **[FOR HUN ONLY]**  Ministry of Health, Romania (<http://legislatie.just.ro/Public/DetaliiDocumentAfis/247076>) **[FOR ROM ONLY]**  <http://cas.cnas.ro/casmb/media/pageFiles/20211005_ORDIN%20Nr.%20887%20din%2027.09.2021-metodologiei%20de%20stabilire%20a%20preturilor%20de%20referinta.pdf> **[FOR ROM ONLY]**  <https://cnas.ro/programe-nationale-de-sanatate-curative/> **[FOR ROM ONLY]**  <https://cnas.ro/programe-nationale-de-sanatate-curative/> **[FOR ROM ONLY]**  **Please change the values if they are incorrect or inaccurate or suggest an appropriate value for missing costs.**   \|  \| **Consumables/devices** \| **Pack size/Unit** \| **[Cost/price]**  **(Range: 0.5x to 5x pre-populated value)** \| \| --- \| --- \| --- \| --- \| \| **1** \| Bi-level positive airway pressure device \| [Insert pre-populated value here] \| [Insert pre-populated value here] \| \| **2** \| Continuous positive airway pressure at fixed pressure device \| [Insert pre-populated value here] \| [Insert pre-populated value here] \| \| **3** \| Dental appliances for sleep apnea \| [Insert pre-populated value here] \| [Insert pre-populated value here] \| \| **4** \| Dry powder inhaler \| [Insert pre-populated value here] \| [Insert pre-populated value here] \| \| **5** \| External loop recorder \| [Insert pre-populated value here] \| [Insert pre-populated value here] \| \| **6** \| Flash glucose monitoring devices (e.g. Freestyle Libre) \| [Insert pre-populated value here] \| [Insert pre-populated value here] \| \| **7** \| Glucometer \| [Insert pre-populated value here] \| [Insert pre-populated value here] \| \| **8** \| Glucose strip \| [Insert pre-populated value here] \| [Insert pre-populated value here] \| \| **9** \| Hand splints \| [Insert pre-populated value here] \| [Insert pre-populated value here] \| \| **10** \| Holter monitor \| [Insert pre-populated value here] \| [Insert pre-populated value here] \| \| **11** \| Insertable cardiac monitor \| [Insert pre-populated value here] \| [Insert pre-populated value here] \| \| **12** \| Insulin pump \| [Insert pre-populated value here] \| [Insert pre-populated value here] \| \| **13** \| Internal loop recorder \| [Insert pre-populated value here] \| [Insert pre-populated value here] \| \| **14** \| Lumbosacral corsets \| [Insert pre-populated value here] \| [Insert pre-populated value here] \| \| **15** \| Metered dose inhaler with spacer \| [Insert pre-populated value here] \| [Insert pre-populated value here] \| \| **16** \| Oral appliances for sleep apnea \| [Insert pre-populated value here] \| [Insert pre-populated value here] \| \| **17** \| Portable hemodialysis machine \| [Insert pre-populated value here] \| [Insert pre-populated value here] \| \| **18** \| Portable peritoneal dialysis machine \| [Insert pre-populated value here] \| [Insert pre-populated value here] \| \| **19** \| Pressured metered dose inhaler \| [Insert pre-populated value here] \| [Insert pre-populated value here] \| \| **20** \| Small volume nebulizer \| [Insert pre-populated value here] \| [Insert pre-populated value here] \| \| **21** \| Soft cervical collars \| [Insert pre-populated value here] \| [Insert pre-populated value here] \| \| **22** \| Spacer \| [Insert pre-populated value here] \| [Insert pre-populated value here] \| \| **23** \| Sphygmomanometer \| [Insert pre-populated value here] \| [Insert pre-populated value here] \| \| **24** \| Trans-telephonic ECG device \| [Insert pre-populated value here] \| [Insert pre-populated value here] \| \| **25** \| Unloader knee braces \| [Insert pre-populated value here] \| [Insert pre-populated value here] \| |
| Q6 | **ASK IF SELECTING >4 AT S4 CODE E**  **SHOW GRID TABLE WITH PRE-FILLED RANGES, ALLOW RESPONDENTS TO OVERWRITE ON CELLS**  **SHOW PROMPT IF INPUT IS OUT OF RANGE SPECIFIED**   1. What is the cost/price per day for each of the following admission types in your hospital? **RANGE: 0.5x to 5x pre-populated value**   The values pre-populated below are estimates obtained from the following sources:  NEAK (without valid social security card) **[FOR HUN ONLY]**  Ministry of Health, Greece (2012 values) **[FOR GR ONLY]**  <http://cas.cnas.ro/casmb/media/pageFiles/20211005_ORDIN%20Nr.%20887%20din%2027.09.2021-metodologiei%20de%20stabilire%20a%20preturilor%20de%20referinta.pdf> **[FOR ROM ONLY]**  <https://cnas.ro/programe-nationale-de-sanatate-curative/> **[FOR ROM ONLY]**  <https://cnas.ro/programe-nationale-de-sanatate-curative/> **[FOR ROM ONLY]**  **Please change the values if they are incorrect or inaccurate or suggest an appropriate value for missing costs.**   \|  \| **Hospital admissions** \| **Cost/price**  **(Range: 0.5x to 5x pre-populated value)** \| \| --- \| --- \| --- \| \| **1** \| In-patient bed cost (per day) \| [Insert pre-populated value here] \| \| **2** \| ICU bed cost (per day) \| [Insert pre-populated value here] \| \| **3** \| ER visit (per day) \| [Insert pre-populated value here] \| |
| Q7 | **ASK IF SELECTING >4 AT S4 CODE F**  **SHOW GRID TABLE WITH PRE-FILLED RANGES, ALLOW RESPONDENTS TO OVERWRITE ON CELLS**  **SHOW PROMPT IF INPUT IS OUT OF RANGE SPECIFIED**   1. What is the cost/price of each of the following medical procedures in your hospital? **RANGE: 0.5x to 5x pre-populated value**   The values pre-populated below are estimates obtained from the following sources:  NEAK (without valid social security card) **[FOR HUN ONLY]**  Ministry of Health, Greece (2012 values) **[FOR GR ONLY]**  **Please change the values if they are incorrect or inaccurate, or suggest an appropriate value for missing costs.**   1. **Please also suggest appropriate references/sources (e.g. DRG codes) [FOR GR ONLY]**  \|  \| **Medical procedures** \| **Cost/price**  **(Range: 0.5x to 5x pre-populated value)** \| **Source/reference (e.g. specific DRG codes used, URLs)** \| \| --- \| --- \| --- \| --- \| \| **1** \| Amputation of diabetic foot/leg \| [Insert pre-populated value here] \|  \| \| **2** \| Arthrodesis \| [Insert pre-populated value here] \|  \| \| **3** \| Arthroscopic procedures \| [Insert pre-populated value here] \|  \| \| **4** \| Atrial flutter ablation \| [Insert pre-populated value here] \|  \| \| **5** \| AV junction ablation \| [Insert pre-populated value here] \|  \| \| **6** \| Biventricular pacemaker implantation \| [Insert pre-populated value here] \|  \| \| **7** \| Blood transfusion \| [Insert pre-populated value here] \|  \| \| **8** \| Bypass procedure e.g. septoplasty, functional rhinoplasty, nasal valve surgery, nasal polypectomy \| [Insert pre-populated value here] \|  \| \| **9** \| Cardioversion \| [Insert pre-populated value here] \|  \| \| **10** \| Cardioverter-defibrillator implantation \| [Insert pre-populated value here] \|  \| \| **11** \| Cataract surgery \| [Insert pre-populated value here] \|  \| \| **12** \| Coronary artery bypass surgery \| [Insert pre-populated value here] \|  \| \| **13** \| Decompressive procedures \| [Insert pre-populated value here] \|  \| \| **14** \| Dialysis \| [Insert pre-populated value here] \|  \| \| **15** \| Dyslipidemia \| [Insert pre-populated value here] \|  \| \| **16** \| Heart transplant \| [Insert pre-populated value here] \|  \| \| **17** \| Heart valve repair or replacement \| [Insert pre-populated value here] \|  \| \| **18** \| Hypopharyngeal procedures e.g. Tongue reduction, partial glossectomy, lingual tonsillectomy, and mandibular advancement \| [Insert pre-populated value here] \|  \| \| **19** \| Ileal bypass **[N/A for RO & GR]** \| [Insert pre-populated value here] \|  \| \| **19.1** \| Implantation of stents **[FOR RO ONLY]** \| [Insert pre-populated value here] \|  \| \| **20** \| Joint replacement (e.g. TKA) \| [Insert pre-populated value here] \|  \| \| **21** \| Kidney transplant \| [Insert pre-populated value here] \|  \| \| **22** \| LDL apheresis **[N/A for RO]** \| [Insert pre-populated value here] \|  \| \| **23** \| Liver transplantation \| [Insert pre-populated value here] \|  \| \| **24** \| Oral & nasal procedures e.g. Uvulopalatopharyngoplasty, palatal advancement, pharyngotonsillectomy, tonsillectomy, and excision of tori mandibularis \| [Insert pre-populated value here] \|  \| \| **25** \| Osteotomy \| [Insert pre-populated value here] \|  \| \| **26** \| Pacemaker implantation \| [Insert pre-populated value here] \|  \| \| **27** \| Percutaneous coronary intervention \| [Insert pre-populated value here] \|  \| \| **28** \| Tracheotomy \| [Insert pre-populated value here] \|  \| \| **29** \| Ventricular assist device implantation \| [Insert pre-populated value here] \|  \| |

**THANK AND CLOSE.**
